# Synthesis of the 8,19‐Epoxysteroid Eurysterol A

**DOI:** 10.1002/chem.202000585

**Published:** 2020-03-09

**Authors:** Ömer Taspinar, Tobias Wilczek, Julian Erver, Martin Breugst, Jörg‐Martin Neudörfl, Hans‐Günther Schmalz

**Affiliations:** ^1^ Department of Chemistry University of Cologne Greinstraße 4 50939 Köln Germany

**Keywords:** C−H activation, natural products, oxa-michael addition, oxidation, remote functionalization, steroids, sulfation

## Abstract

We report the first chemical synthesis of eurysterol A, a cytotoxic and antifungal marine steroidal sulfate with a unique C8−C19 oxy‐bridged cholestane skeleton. After C19 hydroxylation of cholesteryl acetate, used as an inexpensive commercial starting material, the challenging oxidative functionalization of ring B was achieved by two different routes to set up a 5α‐hydroxy‐7‐en‐6‐one moiety. As a key step, an intramolecular oxa‐Michael addition was exploited to close the oxy‐bridge (8β,19‐epoxy unit). DFT calculations show this reversible transformation being exergonic by about −30 kJ mol^−1^. Along the optimized (scalable) synthetic sequence, the target natural product was obtained in only 11 steps in 5 % overall yield. In addition, an access to (isomeric) 7β,19‐epoxy steroids with a previously unknown pentacyclic ring system was discovered.

## Graphical abstract

**From an animal sterol to a bioactive marine natural product**: The first synthesis of eurysterol A, a cytotoxic and antifungal sulfated sterol with a unique 8,19‐epoxy bridge spanning ring B, was elaborated exploiting an intramolecular oxa‐Michael reaction as a key step.

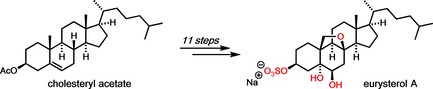

Marine organisms represent a rich source of structurally novel natural products with interesting pharmacological activities.^1^ An example are eurysterols A (**1**) and B (**2**), two mono‐sulfated steroids^2^ isolated in 2007 from a sponge of the genus *Euryspongia* collected in Palau.^3^ These compounds were found to display cytotoxicity against HCT‐116 human carcinoma cells as well as antifungal properties against amphotericin B‐resistant strains of *Candida albicans*.^3^ Structurally, the eurysterols are characterized by an unusual 8,19‐epoxy cholestane skeleton with a sodium sulfate group at C3 and a 5α,6β‐diol moiety (Figure 1). The only known natural product with the same pentacyclic core structure is abscisterol D (**3**), a metabolite produced by the fungus *Cryptosporiopsis abietina*.^4^

**Figure 1:**
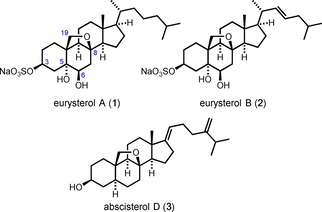
Structures of eurysterols A and B and abscisterol D.

Due to their unique and synthetically unscaled ring skeleton, their interesting biological properties, and their limited availability from natural sources, the eurysterols represent attractive and challenging target molecules for chemical synthesis. We here disclose the results of a study which has culminated in the elaboration of a first (and even scalable) synthesis of eurysterol A.

Our retrosynthetic analysis (Scheme 1) started with the consideration that the sodium sulfate group should be installed at a late stage of the synthesis since it renders the molecule water‐soluble. We thus selected the dihydroxyketone **4** as a pre‐target molecule which could be selectively sulfated at the secondary OH group followed by diastereoselective reduction of the keto function. As a key step, we envisioned to exploit an oxa‐Michael addition^5^ to close the oxy‐bridge between C8 and C19.

**Scheme 1:**
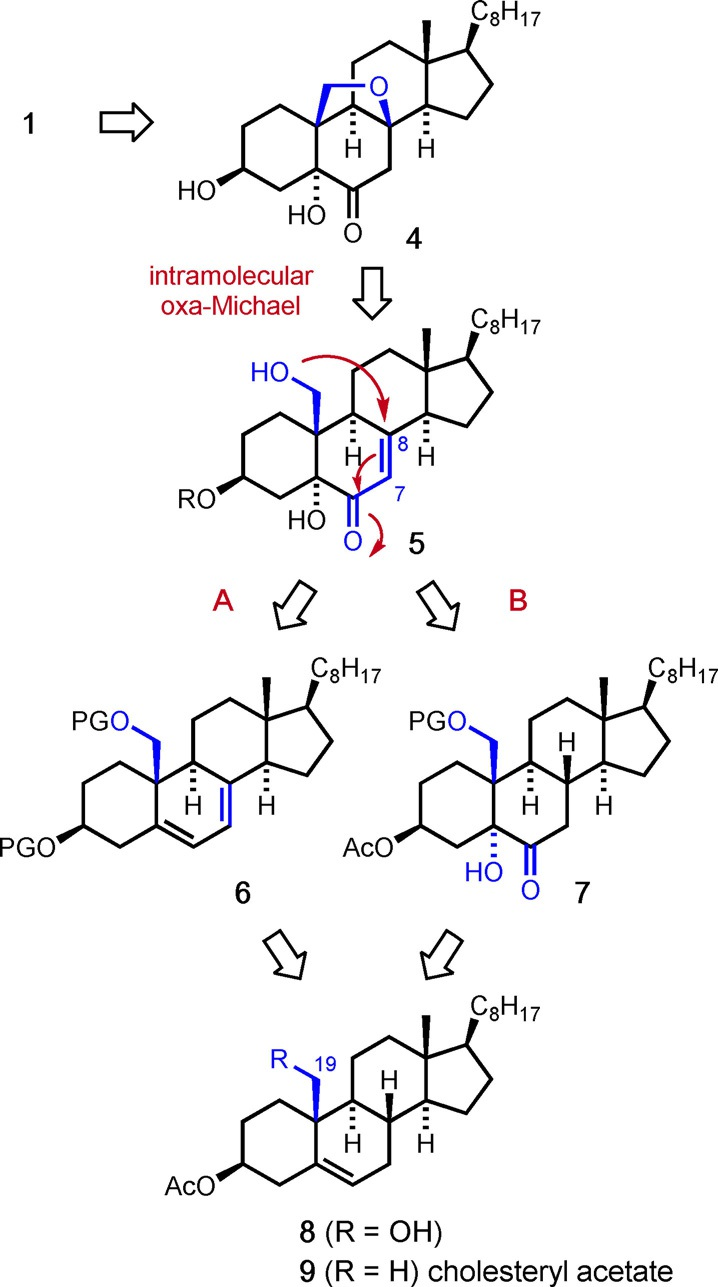
Retrosynthetic analysis of eurysterol A (**1**).

It appeared feasible to us to prepare the required enone of type **5** by semi‐synthesis from commercial cholesteryl acetate **9** through the known 19‐hydroxylated derivative **8**.^6^ However, a crucial aspect of the synthetic plan was the oxidative functionalization of ring B, that is, the conversion of **8** into **5**. This task ought to be achieved by two different approaches. As a first option (route A), we considered a regioselective oxidation of the Δ^5^‐double bond of a 7,8‐dehydro‐steroid of type **6**. Alternatively (route B), the double bond in **9** could first be oxidized to give a ketol intermediate of type **7** which then would have to be converted into the enone **5** by α,β‐dehydrogenation of the ketone.

As a first task, we converted cholesteryl acetate **9** into the 19‐hydroxy derivative **8** following the method of Heusler and Kalvoda (Scheme 2).^6^, ^7^ This method exploits the 1,3‐diaxial vicinity of the 6β‐OH group to the angular C19‐methyl group in the bromohydrin intermediate **10** to achieve a remote functionalization by radical hydrogen atom transfer. In contrast to the original protocol, we performed the (photo‐mediated) hypoiodite reaction employing (diacetoxyiodo)benzene (DIB) in cyclohexane^8^ instead of toxic lead tetraacetate in benzene. After treatment of the resulting 6,19‐epoxy compound **11** with zinc in AcOH the desired alcohol **8** was obtained in 43 % overall yield on a 25 gram scale and with a single chromatographic purification at the very end of the sequence.

**Scheme 2:**
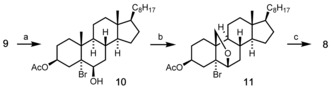
Preparation of 19‐hydroxy‐cholesteryl acetate (**8**). Reagents and conditions: a) NBA (1.5 equiv), HClO_4_ (0.1 N), dioxane, 0 °C to RT, 2 h; b) DIB (1.5 equiv), I_2_ (1.2 equiv), *hv*, *c*‐Hex, reflux, 1 h; c) Zn (5 equiv), AcOH (14 equiv), *i*PrOH, reflux, 3 h, 43 % (over 3 steps) on a 25‐gram scale. NBA=*N*‐bromo acetamide, DIB=(diacetoxyiodo)benzene.

According to strategy A (Scheme 1), we next investigated the preparation and oxidation of cholesta‐5,7‐dien‐3,19‐diol derivatives of type **6**. After protecting the free alcohol group of **8** as a TBS ether (**12**) the O‐acetyl group at C3 was replaced by a MOM group to give **13** (Scheme 3). This was necessary to insure compatibility with the conditions of the later Bamford–Stevens elimination.^9^ Originally, the allylic oxidation of the alkene **13** to the enone **14** was performed using the Collins–Ratcliffe reagent (CrO_3_ **⋅**2 py, 77 %; see the Supporting Information for details).^10^ However, to avoid the use of stoichiometric amounts of toxic chromium(VI) we also tested other methods and found that the transformation of **13** to **14** could also be achieved in comparable yield (71 %) with *tert*‐butyl hydroperoxide as the main oxidant in the presence of catalytic amounts (0.7 mol %) of RuCl_3_.^11^ The keto group of **14** was then converted into the corresponding tosylhydrazone (*syn*/*anti* mixture) from which the Δ^5, 7^‐diene **15** was obtained in high yield upon treatment with LiH in refluxing toluene.^12^ Initial attempts to regioselectively oxidize the Δ^5^‐double bond of **15** employing different Cr^VI^ reagents^13^ only gave low yields. Moreover, the desired ketol product obtained from **15** using in situ generated RuO_4_ 14 did not yield any of the desired 8,19‐epoxy product **18** upon TBAF‐mediated deprotection of the TBS ether (see Supporting Information for details).^15^ Therefore, we replaced the TBS by an acetyl protecting group and examined the oxidation of the resulting diene **16** which proved to be particularly difficult. All attempts to achieve this reaction by OsO_4_‐catalyzed dihydroxylation^16^ or by methyltrioxorhenium‐catalyzed reaction with urea‐H_2_O_2_ 17 failed. Only the protocol of Plietker (using NaIO_4_ in the presence of CeCl_3_ and catalytic amounts of RuCl_3_)^18^ afforded the desired α‐diol, albeit in only 21 % yield (61 % based on recovered **16**). Nevertheless, oxidation of the allylic OH group at C6 with MnO_2_ afforded the desired ketol **17** which could be used to study the planned key step of the synthesis, that is, the construction of the 8,19‐epoxy bridge through intramolecular oxa‐Michael addition.

**Scheme 3:**
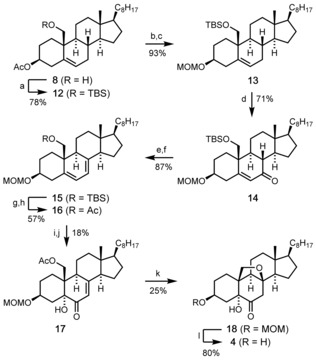
Synthesis of the pre‐target molecule **4** following route A. Reagents and conditions: a) TBSCl (2 equiv), imidazole (1.5 equiv), CH_2_Cl_2_, RT, 16 h, 78 %; b) K_2_CO_3_ (1.5 equiv), MeOH/THF/H_2_O (4:2:1), RT, 6 h; c) MOMCl (1.5 equiv), DIPEA (2.4 equiv), CH_2_Cl_2_, RT, 12 h, 93 % (over 2 steps); d) RuCl_3_ **⋅** *x* H_2_O (0.7 mol %), TBHP (20 equiv), *c*‐Hex, RT, 24 h, 71 %; e) TsNHNH_2_, (5 equiv), EtOH, reflux, 16 h, quant.; f) LiH (60 equiv), toluene, reflux, 5 h, 87 %; g) TBAF**⋅**THF (5 equiv), THF, RT, 16 h; h) Ac_2_O (11 equiv), DMAP (0.03 equiv), pyridine, RT, 12 h, 57 % (over 2 steps); i) RuCl_3_ **⋅** *x* H_2_O (5 mol %), NaIO_4_ (1.5 equiv), CeCl_3_ **⋅**7 H_2_O (0.2 equiv), EtOAc/MeCN/H_2_O (3:3:1), 0 °C to RT, 1 h, 21 % (61 % brsm); j) MnO_2_ (10 equiv), CH_2_Cl_2_, RT, 12 h, 29 %; k) K_2_CO_3_ (12 equiv), MeOH, RT, 12 h, 25 %; l) ZnBr_2_ (2.4 equiv), *n*‐PrSH (4 equiv), CH_2_Cl_2_, 0 °C to RT, 2 h, 80 %. TBSCl=*tert*‐butyldimethylsilyl chloride, MOMCl=chloromethyl methyl ether, DIPEA=*N*,*N*‐diisopropylethylamine, TsNHNH_2_=*para*‐toluenesulfonyl hydrazide, TBAF=tetra‐*n‐butyl*‐ammonium fluoride.

Much to our satisfaction, the desired cyclization product **18** was indeed formed upon treatment of **17** with K_2_CO_3_ in MeOH. Subsequent removal of the MOM protecting group under mild conditions^19^ finally afforded the anticipated pentacyclic pre‐target compound **4**, the structure of which was unambiguously confirmed by X‐ray crystallography (Figure 2).^20^

**Figure 2:**
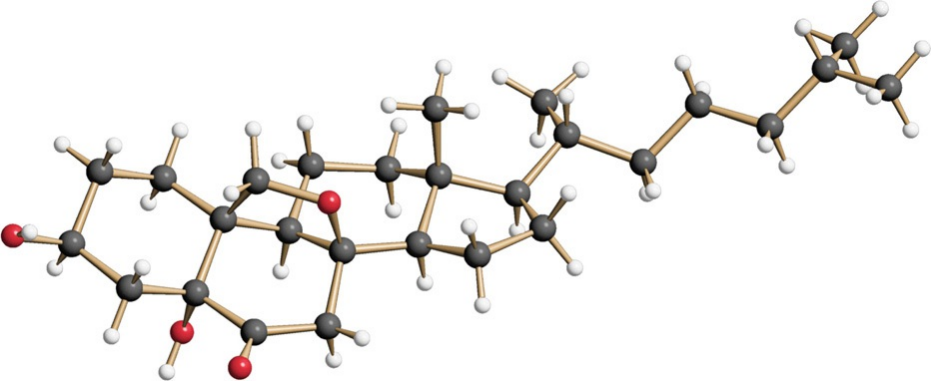
Structure of the pentacyclic compound **4** in the crystalline state.

Having thus demonstrated the general feasibility of our synthetic strategy, the unsatisfying efficiency of the developed sequence (Scheme 3) prompted us to also investigate route B (compare Scheme 1). After considerable experimentation (testing different combinations of protecting groups), we came up with the improved sequence outlined in Scheme 4. In this case, the OH group of **8** was protected by acetylation and the resulting diacetate **19** was converted to the ketol **20** by Os‐catalyzed dihydroxylation of the Δ^5^‐double bond in presence of citric acid^21^ and subsequent Dess–Martin oxidation.^22^ The installation of the Δ^7^‐double bond by direct dehydrogenation of **20** could not be achieved under the conditions (IBX in DMSO) of Nicolaou.^23^ However, we succeeded in achieving the desired α,β‐dehydrogenation through an α‐bromination/elimination sequence.^24^ Thus, reaction of ketol **20** with bromine in the presence of a catalytic amount of HBr in acetic acid yielded the α‐brominated intermediate which upon treatment with LiBr and Li_2_CO_3_ in refluxing DMF afforded the enone **21** in satisfying yield.

**Scheme 4:**
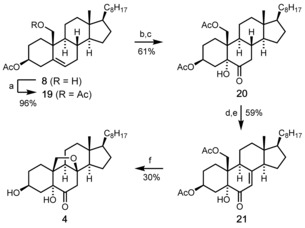
Improved synthesis of the pre‐target molecule **4** following route B. Reagents and conditions: a) Ac_2_O (10 equiv), DMAP (0.03 equiv), pyridine, RT, 3 h, 96 %; b) OsO_4_ (10 mol %), NMO (2 equiv), citric acid (2 equiv), acetone/*t*BuOH/H_2_O (1:1:1), RT, 24 h, 62 %; c) DMP (1.2 equiv), CH_2_Cl_2_, RT, 10 h, 98 %; d) Br_2_ (3 equiv), HBr (cat), AcOH, 50 °C, 1 h, 90 %; e) Li_2_CO_3_ (10 equiv), LiBr (3 equiv), DMF, 150 °C, 4 h, 66 %; f) K_2_CO_3_ (12 equiv), MeOH, RT, 14 h, 30 %. DMAP=4‐dimethylaminopyridine, NMO=*N*‐morpholine *N*‐oxide, DMP=Dess–Martin periodinane.

Reaction of enone **21** with K_2_CO_3_ in methanol at room temperature for 14 hours not only resulted in the cleavage of both acetoxy groups but also (again) in a spontaneous cyclization (intramolecular oxa‐Michael reaction) to furnish the 8β,19‐epoxy steroid **4** in 30 % isolated yield. Notably, despite the full conversion of the starting material **21**, we were unable to isolate any side product.

To shed some light on the thermodynamics of the (reversible) oxa‐Michael reaction **21**→**4** (Scheme 3), we calculated the relative Gibbs free energies for the model systems depicted in Scheme 5 at the DLPNO‐CCSD(T)/def2‐TZVPPD/SMD(MeOH)//TPSS‐D3BJ/6–31+G(d,p)/SMD(MeOH) level of theory.^25^ Based on our calculations, we can conclude that the intramolecular cyclizations of both the neutral system **A** as well as the anionic system **A’** are exergonic reactions und clearly favor the cyclized products **B** and **B**’ by approx. 30 kJ mol^−1^.

**Scheme 5:**
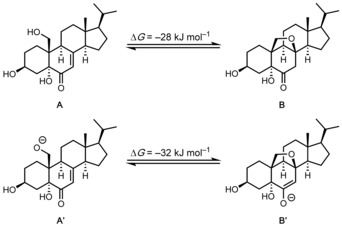
Calculated reaction free energies (Δ*G*) for the intramolecular oxa‐Michael reactions of **A** and **A’**.

Although attempts to improve the yield of the cyclization step by variation of the reaction conditions were not successful so far, the improved route (Scheme 4) afforded comfortable amounts of the pre‐target compound **4** enabling us to tackle the end game of the synthesis (Scheme 6). After considerable experimentation we found that sulfonation of **4** proceeds smoothly using chlorosulfuric acid in pyridine to afford the water‐soluble compound **22** in quantitative yield after simple removal of all volatiles. Finally, the reduction of the C6‐keto group with NaBH_4_ cleanly gave rise to the sodium salt of eurysterol A (**1**) as a white crystalline solid, also in virtually quantitative yield. Remarkably, these last two steps did not require extractive work‐up and the target product **1** was isolated in isomerically pure form after simple chromatography.

**Scheme 6:**
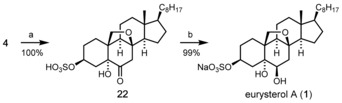
Completion of the synthesis of eurysterol A (**1**). Reagents and conditions: a) HSO_3_Cl (2 equiv), pyridine, −10 °C, 30 min, quant.; b) NaBH_4_, MeOH, 0 °C to RT, 1 h, 99 %.

The comparison of the spectroscopic data of our synthetic product with those reported for natural eurysterol A (**1**) confirmed the identity of both samples (see Supporting Information). Moreover, we succeeded in growing crystals of eurysterol A (**1**) what allowed us to determine its precise structure by means of X‐ray crystallography. While the constitutional and configurational assignments were confirmed, the structure also revealed an intramolecular hydrogen bridge between the axial OH group at C6 and the epoxy bridge (Figure 3).

**Figure 3:**
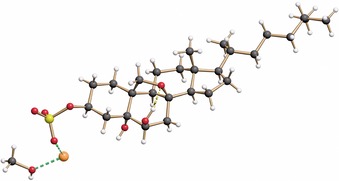
Structure of eurysterol A (**1**) in the crystalline state.

As an additional outcome of the synthetic endeavor described herein, we by chance also discovered a synthetic access to *iso*‐**4**, which is a constitutional isomer of **4** and a first representative of the so far completely undescribed class of 7,19‐epoxy steroids. As shown in Scheme 7, we oxidized the double bond of MOM‐protected 19‐hydroxy‐cholesteryl acetate to the corresponding ketol related to **20**. However, in this case, the α‐bromination of the ketone went along with the cleavage of the MOM group to give the hemiacetal **23**. To our surprise, the envisaged elimination then did not take place upon heating of **23** with Li_2_CO_3_/LiBr in DMF. Instead, the 7,19‐epoxy bridge was formed, probably by S_N_2 reaction of the anionic intermediate **23’** to give *iso*‐**4** after methanolytic cleavage of the acetate protecting group in high yield (Scheme 7).

**Scheme 7:**
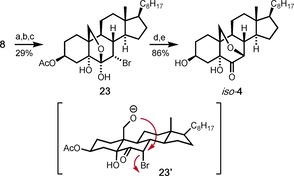
Synthesis of the 7,19‐epoxysteroid *iso*‐**4**. Reagents and conditions: a) MOMCl (1.5 equiv), DIPEA (2.5 equiv), CH_2_Cl_2_, RT, 18 h, 82 %; b) *m*CPBA (1.7 equiv), CH_2_Cl_2_, RT, 1 h; then CrO_3_ (5.4 equiv), acetone/H_2_O (4:1), 0 °C to RT, 2 h, 63 %; c) Br_2_ (3.5 equiv), HBr (cat), AcOH, 60 °C, 33 h, 56 %; d) Li_2_CO_3_ (4.6 equiv), LiBr (3 equiv), DMF, 100 °C, 2 h, 94 %; e) K_2_CO_3_ (1.5 equiv), MeOH, RT, 1 h, 92 %.

In conclusion, we have elaborated an efficient semi‐synthesis of eurysterol A (**1**) starting from inexpensive cholesteryl acetate. The synthetic sequence (11 steps; 5 % overall yield), which opens an entry into the class of 8,19‐epoxy steroids for the first time, is scalable, requires only a single protection step, and exploits an intramolecular oxa‐Michael addition as a key step to close the oxy‐bridge between C8 and C19. Importantly, a novel, practical and highly efficient protocol for the final sulfation step was introduced as well. The developed route allows the production of substantial amounts of the target sterol (150 mg prepared). In addition, we also discovered an efficient entry towards 7β,19‐epoxy steroids, a previously unknown class of compounds with a slightly different (isomeric) pentacyclic ring system.

Thus, this work paves the way for the future exploration of the eurysterols and related epoxy steroids as potential bioactive compounds. Considering the ongoing interest in the synthesis of steroids with unusual oxidation and ring patterns^26^ we are convinced that the developed protocols for the B‐ring functionalization of 19‐oxygenated steroids will prove of value also for other researchers in the future.

## Conflict of interest

The authors declare no conflict of interest.

## Supporting information

As a service to our authors and readers, this journal provides supporting information supplied by the authors. Such materials are peer reviewed and may be re‐organized for online delivery, but are not copy‐edited or typeset. Technical support issues arising from supporting information (other than missing files) should be addressed to the authors.

Supplementary
