# Supplementary material for: Synthesis of the 8,19‐Epoxysteroid Eurysterol A
Source: Chemistry. 2020 Mar 9;26(19):4256–60. doi: 10.1002/chem.202000585 (PMC7187428; doi:10.1002/chem.202000585)
Supplement: Supplementary file 1 — Supplementary [file CHEM-26-4256-s001.pdf]

CHEMISTRY
A European Journal

Supporting Information

# Synthesis of the 8,19-Epoxysteroid Eurysterol A

Ömer Taspinar, Tobias Wilczek, Julian Erver, Martin Breugst, Jörg-Martin Neudörfl, and Hans-Günther Schmalz*[a]

chem_202000585_sm_miscellaneous_information.pdf

**Table of Contents**


1. **Overview Schemes (Synthetic Pathways)** ........ 2
2. **General Experimental Information** ........ 4
3. **Experimental Procedures and Data** ........ 5
4. **$^1$H and $^{13}$C NMR Spectra** ........ 40
5. **Comparison of Synthetic Eurysterol A with Natural Isolate** ........ 66
6. **X-ray Crystallographic Data** ........ 70
7. **Computational Investigation of the Key Step** ........ 73
8. **References** ........ 79

# 1. Overview Schemes (Synthetic Pathways)

## Synthesis of 19-hydroxy cholesteryl acetate

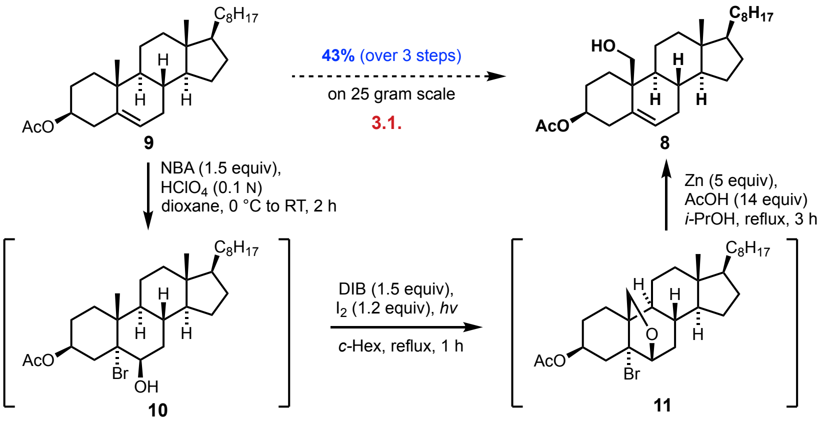


## Conversion of 19-hydroxy cholesteryl acetate (8) into the pre-target compound 4 according to route A

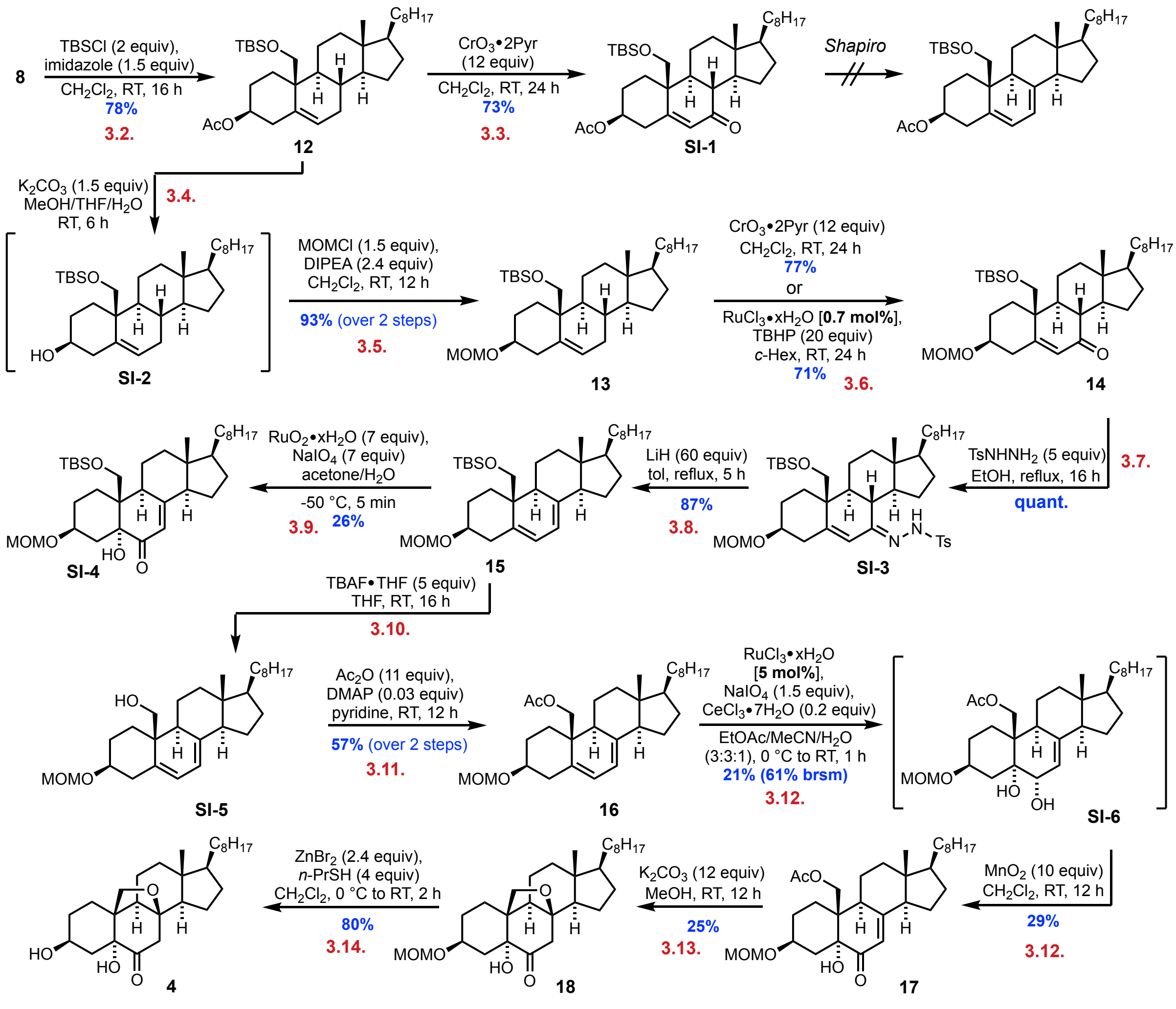


**Scheme SI-1.** Overview on experiments **3.1.** to **3.14.**

## Conversion of 19-hydroxy cholesteryl acetate (8) into the pre-target compound 4 according to route B

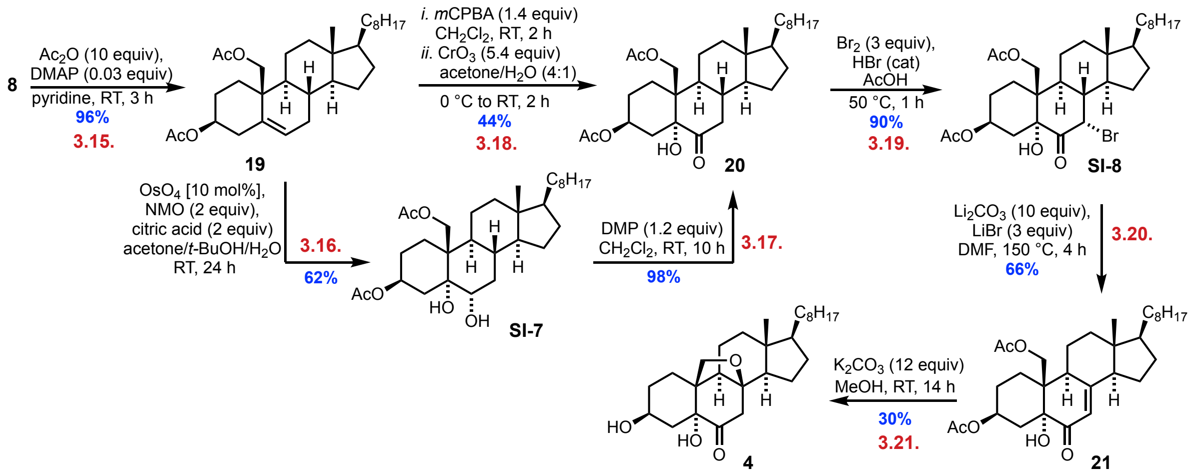


## Conversion of the pre-target compound 4 into eurysterol A

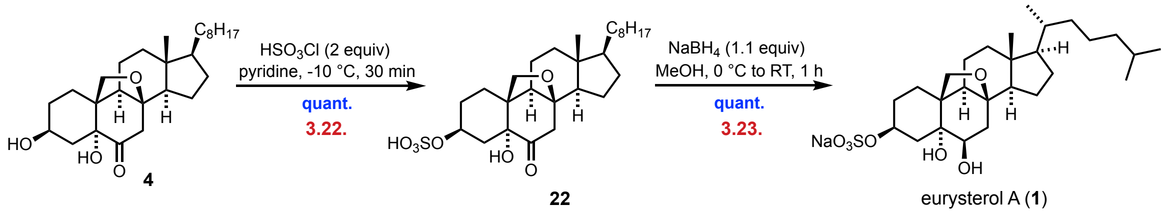


## Conversion of 19-hydroxy cholesteryl acetate (8) into the isomeric pre-target compound *iso*-4

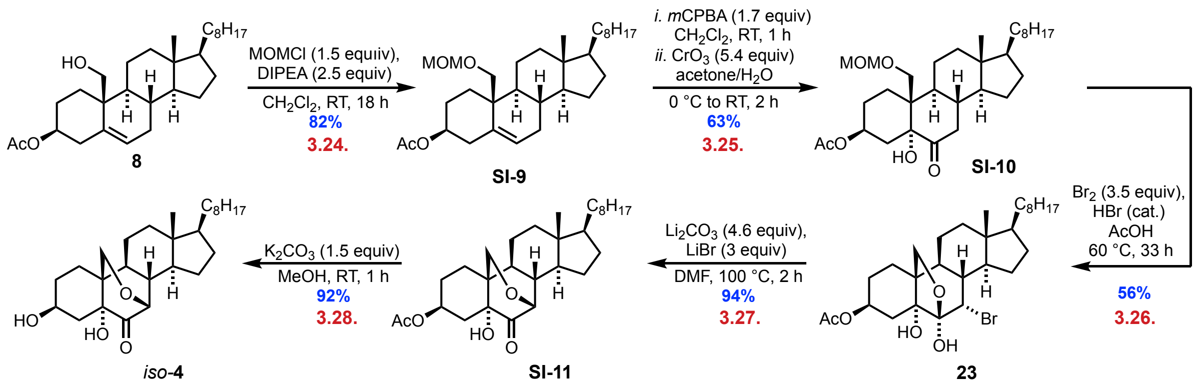


**Scheme SI-2.** Overview on experiments **3.15.** to **3.28.**

## 2. General Experimental Information

### 2.1 Analytical Methods

**2.1.1 Nuclear magnetic resonance spectroscopy (NMR)** $^{1}$H and $^{13}$C NMR spectra were obtained in $CDCl_3$ or $CD_3OD$ at ambient temperature on a *Bruker* Avance II 500 (500 MHz), *Bruker* Avance III 500 (500 MHz) or Avance II+ 600 spectrometer (600 MHz). Chemical shifts for the $^{1}$H and $^{13}$C NMR spectra were recorded in parts per million (ppm) on δ scale from an internal standard of residual tetramethylsilane (TMS, 0 ppm). Signals are reported in relative to the residual signal of the non-deuterated solvent ($^{1}$H: 7.26 ppm and $^{13}$C: 77.0 for $CDCl_3$; $^{1}$H: 3.31 ppm and $^{13}$C: 49.0 ppm for $CD_3OD$). Abbreviations of fine structure of $^{1}$H NMR spectra used: s = singlet, d = doublet, dd = doublet of doublets, ddd = doublet of doublets of doublets, td = triplet of doublets, qd = quartet of doublets, t = triplet, dt = doublet of triplets, tt = triplet of triplets, m = multiplet, br = broad. Scalar coupling constants (*J*) are given in hertz (Hz). All $^{13}$C NMR spectra (except for eurysterol A) were recorded using APT sequence with complete proton decoupling. Abbreviations of fine structure of $^{13}$C NMR spectra used: s for quaternary C, d for CH, t for $CH_2$ and q for $CH_3$. The non-trivial assignments were determined by $^{1}$H,$^{1}$H-COSY, $^{1}$H,$^{1}$H-NOESY, $^{1}$H,$^{13}$C-HSQCed/HMQC and $^{1}$H,$^{13}$C-HMBC spectra.

**2.1.2 Fourier transform infrared spectroscopy (FT-IR)** Infrared (IR) spectra were obtained in 4000 – 500 $cm^{-1}$ range using a *PerkinElmer* Spectrum Two FT-IR spectrometer using ATR technique. Absorption bands are reported in $cm^{-1}$. Abbreviations of fine structure of spectra used: w = weak, m = middle, strong, s = strong, vs = very strong, bw = broad weak.

**2.1.3 High resolution electrospray and electron impact ionization-mass spectrometry (HR-MS)** High resolution mass spectra were performed on a *Thermo Scientific* LTQ Orbitrap XL spectrometer (ESI). The spray voltage (3.4 kV), capillary voltage (3.0 V), tube lens voltage (3.0 V) and capillary temperature of 275 °C were applied as ESI conditions. To generate a stable electrospray sheath gas and sweep gas were used (Nitrogen 5.0, Linde, ≥ 99.999%).

**2.1.4 Specific optical rotation ($[a]_{\lambda}^{T}$)** Optical rotation values were determined with an *Anton Paar* MCP 200 Polarimeter. The measurements were performered at ambient temperature in chloroform or methanol. The concentration c of the measured solution (g/100 mL) are preceded in squared brackets with optical rotation values. Letter D represents the Fraunhofer d-lines at 589 nm.

### 2.2. Chromatographic Methods

**2.2.1 Thin-layer chromatography (TLC)** Reactions were monitored on TLC on silica gel 60 $F_{254}$ (layer thickness 0.25 mm, *Merck*) visualized with a solution of cerium ammonium molybdate (CAM) (2 g of molybdophosphoric acid, 1 g of cerium(IV) sulfate tetrahydrate in 10 mL of sulfuric acid and 90 mL of water) and subsequent heating.

**1.2.2 Column chromatography** Flash column chromatography was performed on silica gel 60 Å (230-400 mesh, *Acros*).

## 2.3 Miscellaneous Information

**2.3.1 Melting-point apparatus** Melting points were determined with a *Büchi* Melting Point B-545 apparatus in open capillary tubes and are uncorrected.

## 2.4 Solvents and Reagents

All sensitive reactions were carried out under argon atmosphere in flame-dried glassware unless otherwise noted. Dichloromethane was distilled from calcium hydride under argon atmosphere. Diethyl ether, tetrahydrofuran and toluene were freshly distilled under argon atmosphere from sodium and benzophenone. All reagents were used as received from commercial sources without further purification unless otherwise noted. The solvents were distilled before use.

# 3. Experimental Procedures and Data

## 3.1 Synthesis of 3*β*-Acetoxy-cholest-$\Delta^5$-en-19*β*-ol (8)[1]

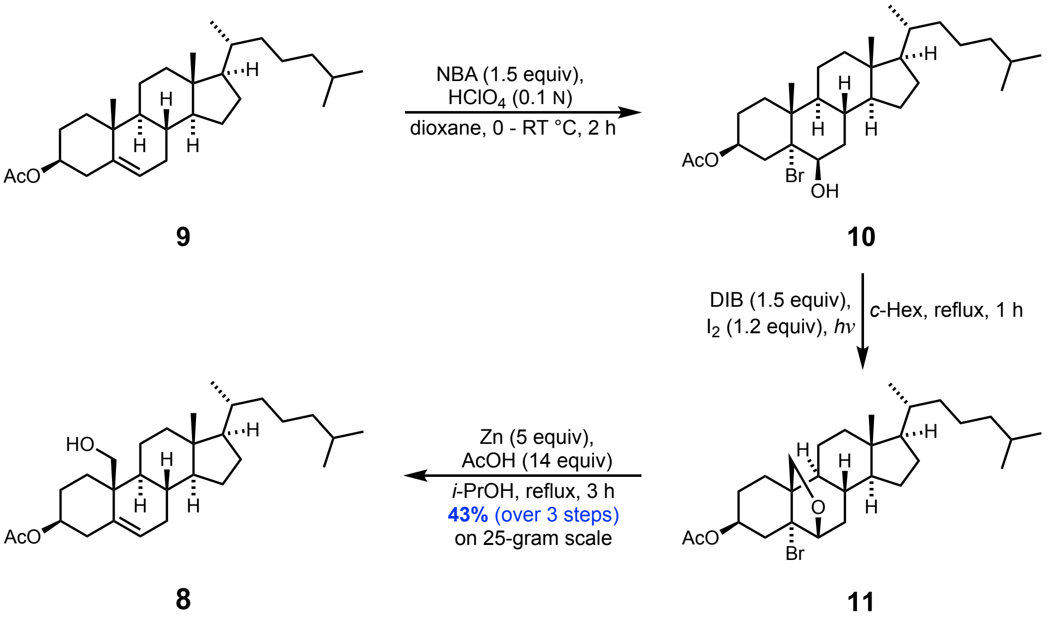


To a solution of 25 g (58 mmol, 1.0 equiv) of cholesteryl acetate (**9**) in dioxane (200 mL) was added 12 g (87 mmol, 1.5 equiv) of *N*-bromoacetamide (NBA) and 40 mL of $HClO_4$ (0.5 N). After stirring the mixture at 0 °C for 1 h in the dark and for further 1 h at ambient temperature excess reagent was quenched by addition of saturated aqueous $Na_2SO_3$ (until discoloration) and water. After extraction with MTBE, the organic layers were washed with brine, dried over $MgSO_4$ and filtrated before the solvent was removed under reduced pressure. The crude product (**10**, beige solid, 31 g) - containing significant amounts (>40%) of the regioisomeric bromohydrin - was directly used in the next step

without further purification. For analytical purposes, a small sample was purified by chromatography.

To a solution of the crude bromohydrin **10** (+ regioisomer) in *c*-Hex (1.5 L) and were added 27.6 g (85.8 mmol, 1.5 equiv) of (diacetoxyiodo)benzene (DIB) and 8.76 g (68.6 mmol, 1.2 equiv) of iodine. The reaction mixture was refluxed for 1 h under irradiation with a 150 W Hg-lamp. The reaction mixture (still purple) was then cooled to ambient temperature and quenched by addition of saturated aqueous $Na_2SO_3$ (until discoloration) and water. After extraction with EtOAc the collected organic layers were washed with brine, dried over $MgSO_4$ and the solvent was removed under reduced pressure. The residual crude product (**11**, brown viscous oil) was used in the next step without further purification. Only for analytical purposes, a small sample was purified by chromatography.

To a stirred solution of the crude ether **11** (prepared as described above) in *i*-PrOH (800 mL) were added 18.9 g (0.29 mol, 5.0 equiv) of Zn (dust) followed by 47 mL (49 g, 0.8 mol, 14.0 equiv) of AcOH. The mixture was then refluxed for 3 h, cooled to ambient temperature and filtrated through a short pad of Celite® to give a clear yellowish solution. The solution was concentrated to a volume of 50 mL and, after addition of water, extracted with MTBE. The collected organic layers were washed with brine, dried over $MgSO_4$ and the solvent was removed under reduced pressure. The residue (brown oil) was purified by silica gel column chromatography (*c*-Hex/EtOAc, 10:1) to give **8** (10.9 g, 24.5 mol, 43% over three steps) as a beige solid.

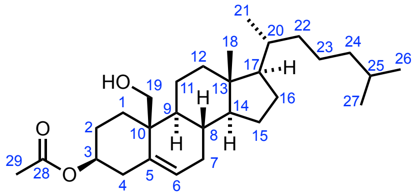


**Formula:** $C_{29}H_{48}O_3$

**Molecular Weight:** 444.70 g/mol

**Yield:** 10.9 g (24.5 mmol, 43% over three steps).

**m.p.:** 112 °C – 114 °C.

**TLC:** $R_f$ = 0.35 (*c*-Hex/EtOAc 3:1), Ce reagent.

**$^1$H NMR** (500 MHz, $CDCl_3$): δ [ppm] = 5.76 (d, *J* = 5.0 Hz, 1H, H-6), 4.66 – 4.60 (m, 1H, H-3), 3.82 (d, *J* = 11.2 Hz, 1H, H-19*β*), 3.61 (dd, *J* = 11.4, 9.1 Hz, 1H, H-19*α*), 2.41 (ddd, *J* = 13.0, 4.9 Hz, 2.1 Hz, 1H, H-4*β*), 2.28 – 2.23 (m, 1H, H-4*α*), 2.02 (s, 3H, H-29), 1.95 (dt, *J* = 13.8, 3.4 Hz, 1H, H-1*α*), 1.90 – 0.93 (m, 26H), 0.90 (d, *J* = 6.5 Hz, 3H, H-21), 0.85 (dd, *J* = 6.6, 2.2 Hz, 3H, H-26, H-27), 0.72 (s, 3H, H-18).

**$^{13}$C NMR** (125 MHz, $CDCl_3$): δ [ppm] = 170.6 (s, C-28), 134.7 (s, C-5), 128.4 (d, C-6), 73.6 (d, C-3), 62.9 (t, C-19), 57.7 (d, C-14), 56.2 (d, C-17), 50.4 (d, C-9), 42.7 (s, C-13), 41.7 (s, C-10), 40.1 (t, C-12), 39.6 (t, C-24), 38.4 (t, C-4), 36.3 (t, C-22), 35.9 (d, C-20), 33.5 (d, C-8), 33.2 (t, C-1), 31.4 (t, C-7), 28.4 (t, C-16), 28.2 (t, C-2), 28.2 (d, C-25), 24.2 (t, C-15), 24.0 (t, C-23), 23.0 (q, C-27), 22.7 (q, C-26), 21.9 (t, C-11), 21.5 (q, C-29), 18.8 (q, C-21), 12.4 (q, C-18).

**FT-IR (ATR):** $\tilde{\nu}$ [cm$^{-1}$] = 3507 (bw), 2944 (s), 2934 (s), 2870 (m), 1734 (m), 1713 (s), 1469 (m), 1444 (m), 1381 (m), 1370 (m), 1254 (s), 1245 (vs), 1029 (vs), 977 (m), 961 (m), 911 (w), 886 (w), 824 (w), 807 (w), 742 (w), 670 (w), 610 (w).

**HR-MS:** (ESI, 70 eV) = *m/z* calcd. for: $C_{29}H_{48}O_3Na^+[M+Na]^+$ 467.3496 u, found: 467.3493 u.

**$[a]^T_\lambda$:** (c = 0.58 g/100 mL, $CHCl_3$) = $[a]^{20}_{365}$: –77.76°, $[a]^{20}_{436}$: –49.94°, $[a]^{20}_{546}$: –28.68°, $[a]^{20}_{579}$: –25.28°, $[a]^{20}_{D}$: –25.28°.

**XRD:** A single crystalline sample was obtained by recrystallization from $CH_2Cl_2$.

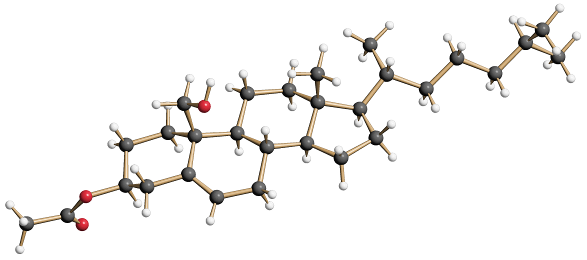

**Figure SI-1.** X-ray crystal structure of 3*β*-Acetoxy-cholest-$\Delta^5$-en-19*β*-ol (**8**).

**Characterization of an analytical sample of intermediate 10**

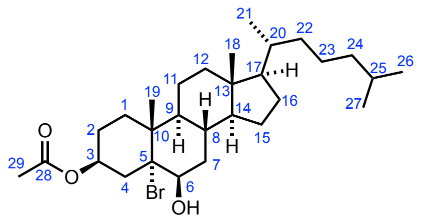


**Formula:** $C_{29}H_{49}BrO_3$

**Molecular Weight:** 525.61 g/mol

**m.p.:** 165 °C – 170 °C.

**TLC:** $R_f$ = 0.33 (*c*-Hex/EtOAc 2:1), Cer-reagent.

**$^1$H NMR** (500 MHz, $CDCl_3$): δ [ppm] = 5.50 – 5.44 (m, 1H, H-3), 4.18 (s, 1H, H-6), 2.53 – 2.48 (dd, *J* = 13.5, 10.5 Hz, 1H, H-4*β*), 2.27 – 2.16 (m, 2H, H-4*α*, 7*β*), 2.03 (s, 3H, H-29), 2.01 – 1.93 (m, 2H, H-2*α*, 12*β*), 1.93 – 1.32 (m, 14H), 1.32 (s, 3H, H-19), 1.29 – 0.95 (m, 10H), 0.90 (d, *J* = 6.5 Hz, 3H, H-21), 0.87 – 0.85 (dd, *J* = 6.6, 2.3 Hz, 6H, H-26, H-27), 0.67 (s, 3H, H-18).

**$^{13}$C NMR** (125 MHz, $CDCl_3$): δ [ppm] = 170.6 (s, C-28), 86.9 (s, C-5), 75.9 (d, C-6), 72.3 (d, C-3), 56.2 (d, C-17), 55.9 (d, C-14), 47.6 (d, C-9), 42.8 (s, C-13), 40.5 (s, C-10), 39.8 (t, C-12), 39.6 (t, C-24), 38.6 (t, C-4), 36.3 (t, C-22), 35.9 (d, C-20), 35.3 (t, C-1), 34.7 (t, C-7), 30.7 (d, C-8), 28.3 (d, C-25), 28.1 (t, C-2), 26.5 (t, C-16), 24.2 (t, C-15), 23.9 (t, C-23), 23.0 (q, C-27), 22.7 (q, C-26), 21.5 (q, C-29), 21.4 (t, C-11), 18.8 (q, C-21), 18.2 (q, C-19), 12.3 (q, C-18).

**FT-IR (ATR):** $\tilde{\nu}$ [$cm^{-1}$] = 3409 (bw), 2935 (m), 2869 (m), 2852 (w), 1704 (vs), 1665 (w), 1467 (w), 1437 (w), 1377 (m), 1366 (m), 1330 (w), 1268 (s), 1238 (m), 1208 (w), 1157 (m), 1050 (m), 1032 (m), 1006 (w), 981 (w), 963 (w), 942 (w), 904 (w), 892 (w), 840 (w), 760 (w), 668 (m).

**HR-MS:** (ESI, 70 eV) = *m/z* calcd. for: $C_{29}H_{49}BrO_3Na^+[M+Na]^+$ 547.27573 u, found: 547.23579 u.

**$[a]^T_\lambda$:** (c = 0.51 g/100 mL, $CHCl_3$) = $[a]^{20}_{365}$: –90.13°, $[a]^{20}_{436}$: –54.58°, $[a]^{20}_{546}$: –30.91°,

$[a]^{20}_{579}$: –27.12°, $[a]^{20}_{D}$: –26.08°.

**Characterization of an analytical sample of intermediate 11**

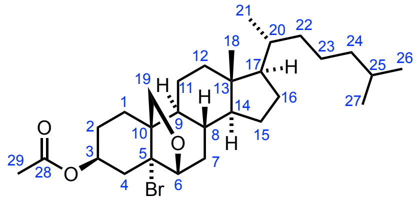


**Formula:** $C_{29}H_{47}BrO_3$

**Molecular Weight:** 523.60 g/mol

**m.p.:** 148 °C – 151 °C.

**TLC:** $R_f$ = 0.44 (*c*-Hex/EtOAc 3:1), Ce reagent.

**$^1$H NMR** (500 MHz, $CDCl_3$): δ [ppm] = 5.22 – 5.16 (m, 1H, H-3), 4.05 (d, *J* = 4.7 Hz, 1H, H-6), 3.91 (d, *J* = 8.4 Hz, 1H, H-19*β*), 3.74 (d, *J* = 8.3 Hz, 1H, H-19*α*), 2.34 – 2.23 (m, 2H, H-4), 2.02 (s, 3H, H-29), 2.01 – 1.96 (m, 3H, H-1*α*, H-7*β*, H-12*β*), 1.85 – 1.79 (m, 1H, H-16*α*), 1.71 – 1.05 (m, 22H), 0.89 (d, *J* = 6.5, 2.3 Hz, 6H, H-26, H-27), 0.69 (s, 3H, H-18).

**$^{13}$C NMR** (125 MHz, $CDCl_3$): δ [ppm] = 170.5 (s, C-28), 82.5 (d, C-6), 74.7 (s, C-5), 70.1 (d, C-3), 67.6 (t, C-19), 56.1 (d, C-17), 54.5 (d, C-14), 48.8 (d, C-9), 46.0 (s, C-10), 43.4 (s, C-13), 41.5 (t, C-4), 39.9 (t, C-12), 39.6 (t, C-24), 36.3 (t, C-22), 35.9 (d, C-20), 33.4 (d, C-8), 33.0 (t, C-1), 28.4 (t, C-16), 28.1 (d, C-25), 27.0 (t, C-7), 23.9 (t, C-23), 23.6 (t, C-15), 23.4 (t, C-2), 22.9 (q, C-27), 22.8 (t, C-11), 22.7 (q, C-26), 21.4 (q, C-29), 18.7 (q, C-21), 12.5 (q, C-18).

**FT-IR (ATR):** $\tilde{\nu}$ [$cm^{-1}$] = 2949 (m), 2933 (m), 2911 (m), 2887 (m), 2870 (m), 1733 (m), 1494 (m), 1467 (w), 1447 (w), 1367 (m), 1304 (w), 1287 (w), 1234 (vs), 1190 (w), 1100 (w), 1047 (m), 1026 (m), 961 (w), 915 (m), 853 (w), 788 (m), 698 (w), 605 (w), 503 (w).

**HR-MS:** (ESI, 70 eV) = *m/z* calcd. for: $C_{29}H_{47}BrO_3Na^+$$[M+Na]^+$ 545.26008 u, found: 545.26009 u.

**$[a]^T_\lambda$:** (c = 0.56 g/100 mL, $CHCl_3$) = $[a]^{20}_{365}$: +15.24°, $[a]^{20}_{436}$: +10.89°, $[a]^{20}_{546}$: +7.14°, $[a]^{20}_{579}$: +6.25°, $[a]^{20}_{D}$: +5.59°.

## 3.2 Synthesis of 3*β*-Acetoxy-19-*tert*-butyldimethylsilyloxy-cholest-Δ$^5$-ene (12)[2]

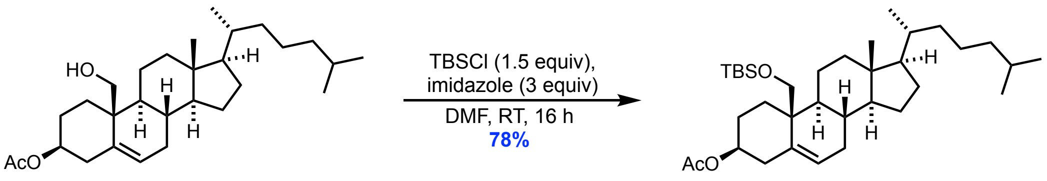


**8** **12**

To a stirred solution of alcohol **8** (9.72 g, 21.9 mmol, 1.0 equiv) in DMF (200 mL) were added 4.94 g (32.8 mmol, 1.5 equiv) of TBSCl followed by 4.64 g (65.6 mmol, 3.0 equiv) of imidazole and stirring was continued at ambient temperature for 16 h. After addition of water the mixture was extracted with

MTBE. The collected organic layers were washed with HCl (1 M, aq.), $NaHCO_3$ (sat. aq.), water and brine, dried over $MgSO_4$ and the solvent was removed under reduced pressure. The residue was purified by silica gel column chromatography (*c*-Hex/EtOAc, 7:1) to give **12** (9.52 g, 17.0 mmol, 78%) as a white solid.

**Formula:** $C_{35}H_{62}O_3Si$

**Molecular Weight:** 558.96 g/mol

**Yield:** 9.52 g (17.0 mmol, 78%).

**m.p.:** 81 °C – 84 °C.

**TLC:** $R_f$ = 0.66 (*c*-Hex/EtOAc 3:1), Ce reagent.

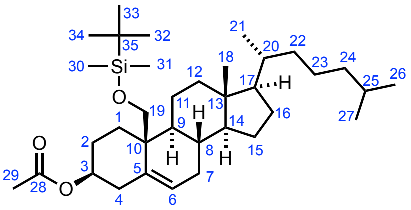

**$^1$H NMR** (500 MHz, $CDCl_3$): δ [ppm] = 5.56 (d, $J$ = 5.2 Hz, 1H, H-6), 4.66 – 4.59 (m, 1H, H-3), 3.73 (d, $J$ = 10.6 Hz, 1H, H-19$\beta$), 3.58 (d, $J$ = 10.5 Hz, 1H, H-19$\alpha$), 2.36 (ddd, $J$ = 13.1, 5.0, 2.3 Hz, 1H, H-4$\beta$), 2.26 – 2.13 (m, 1H, H-4$\alpha$), 2.15 (dt, $J$ = 13.5, 3.6 Hz, 1H, H-1$\beta$), 2.03 (s, 3H, H-29), 2.01 – 1.94 (m, 2H, H-7, H-12$\beta$), 1.85 – 1.77 (m, 3H, H-1$\alpha$, H-2$\alpha$, H-16$\alpha$), 1.72 – 0.94 (m, 20H), 0.91 (d, $J$ = 6.6 Hz, 3H, H-21), 0.87 (s, 9H, H-32, H-33, H-34), 0.86 (dd, $J$ = 6.6, 2.3 Hz, 6H, H-26, H-27), 0.70 (s, 3H, H-18), 0.04 (d, $J$ = 4.8 Hz, 6H, H-30, H-31).

**$^{13}$C NMR** (125 MHz, $CDCl_3$): δ [ppm] = 170.7 (s, C-28), 135.8 (s, C-5), 126.3 (d, C-6), 73.9 (d, C-3), 63.2 (t, C-19), 57.4 (d, C-17), 56.2 (d, C-14), 50.6 (d, C-9), 42.6 (s, C-13), 41.5 (s, C-10), 40.3 (t, C-12), 39.7 (t, C-24), 38.8 (t, C-4), 36.3 (t, C-22), 35.9 (d, C-20), 32.8 (d, C-8), 32.5 (t, C-1), 31.7 (t, C-7), 28.4 (t, C-2), 28.4 (t, C-16), 28.2 (d, C-25), 26.0 (q, C-32, C-33, C-34), 24.4 (t, C-15), 24.0 (t, C-23), 23.0 (q, C-27), 22.7 (q, C-26), 22.0 (t, C-11), 21.6 (q, C-29), 18.9 (q, C-21), 18.3 (s, C-35), 12.3 (q, C-18), –5.4 (q, C-30/31), –5.5 (q, C-30/31).

**FT-IR (ATR):** $\tilde{\nu}$ [cm$^{-1}$] = 2950 (m), 2933 (m), 2871 (m), 2851 (m), 1736 (m), 1727 (m), 1470 (m), 1430 (w), 1367 (m), 1244 (vs), 1092 (vs), 1039 (s), 1005 (w), 971 (w), 911 (w), 892 (w), 868 (m), 836 (s), 800 (m), 773 (s), 703 (w), 676 (w), 624 (w), 601 (w), 589 (w).

**HR-MS:** (ESI, 70 eV) = *m/z* calcd. for: $C_{35}H_{62}O_3Si^+$[M+H]$^+$ 581.43604 u, found: 581.43586 u.

**$[a]_\lambda^T$:** (c = 0.59 g/100 mL, $CHCl_3$) = $[a]_{365}^{20}$: –165.59°, $[a]_{436}^{20}$: –100.74°, $[a]_{546}^{20}$: –56.78°, $[a]_{579}^{20}$: –49.38°, $[a]_{D}^{20}$: –48.03°.

### 3.3. Synthesis of 3*β*-Acetoxy-19-*tert*-butyldimethylsilyloxy-cholest-$\Delta^5$-en-7-one (SI-1)

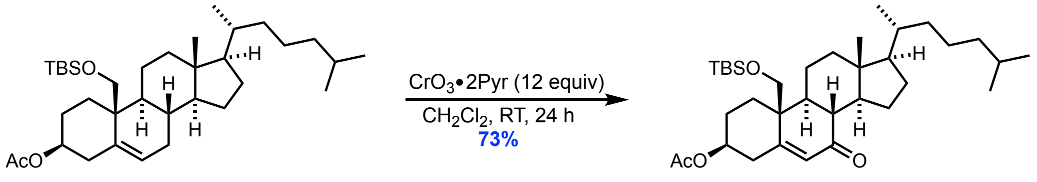


**12** **SI-1**

To a rapidly stirred solution of pyridine (7.47 mL, 92.5 mmol, 18.0 equiv) in anhydrous $CH_2Cl_2$ (250 mL) were added under an atmosphere of dry argon 4.63 g (46.3 mmol, 9.0 equiv) of $CrO_3$. The red solution was stirred for 20 min at ambient temperature before 2.88 g (5.1 mmol, 1.0 equiv) of the alkene **12** in $CH_2Cl_2$ (20 mL) were added in one portion. Stirring was continued at ambient temperature for 17 h. Next day, an additional portion of $CrO_3 \cdot 2$Pyr complex (prepared from 1.54 g (15.4 mmol, 3.0 equiv) $CrO_3$ and 1.24 mL (78.5 mmol, 6.0 equiv) pyridine in 120 mL $CH_2Cl_2$) was added to the reaction mixture and the stirring was continued for further 7 h. The mixture was diluted with 500 mL of MTBE and washed with $NaHCO_3$ (sat. aq.), HCl (1 M, aq.), $NaHCO_3$ (sat. aq.) and brine. The combined organic layers were dried over $MgSO_4$ and the solvent was removed under reduced pressure. The residue (brown oil) was purified by silica gel column chromatography (*c*-Hex/EtOAc, 5:1) to yield enone **SI-1** (2.16 g, 3.77 mmol, 73%) as a white solid.

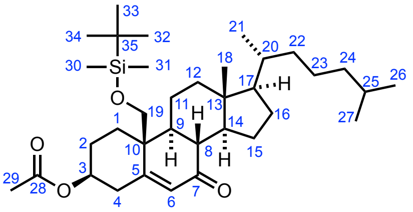

**Formula:** $C_{35}H_{60}O_4Si$

**Molecular Weight:** 572.95 g/mol

**Yield:** 2.16 g (3.77 mmol, 73%).

**TLC:** $R_f$ = 0.47 (*c*-Hex/EtOAc 3:1), Ce reagent.

**$^1$H NMR** (500 MHz, $CDCl_3$): δ [ppm] = 5.86 (d, $J$ = 1.5 Hz, 1H, H-6), 4.76 – 4.70 (m, 1H, H-3), 3.90 (d, $J$ = 10.6 Hz, 1H, H-19*β*), 3.82 (d, $J$ = 10.5 Hz, 1H, H-19*α*), 2.82 (dd, $J$ = 12.5, 10.4 Hz, 1H, H-8), 2.58 (ddd, $J$ = 13.7, 4.9, 2.3 Hz, 1H, H-4*β*), 2.42 – 2.33 (m, 2H, H-4*α*, H-23), 2.05 (s, 3H, H-29), 1.95 – 1.03 (m, 22H), 0.91 (d, $J$ = 6.5 Hz, 3H, H-21), 0.85 (d, $J$ = 6.5 Hz, 3H, H-21), 0.85 (d, $J$ = 5.4 Hz, 15H, H-26, H-27, H-32, H-33, H-34), 0.70 (s, 3H, H-18), 0.06 (s, 3H, H-30/31), 0.03 (s, 3H, H-30/31).

**$^{13}$C NMR** (125 MHz, $CDCl_3$): δ [ppm] = 202.7 (s, C-7), 170.4 (s, C-28), 159.5 (s, C-5), 129.7 (d, C-6), 72.3 (d, C-3), 63.9 (t, C-19), 54.9 (d, C-17), 51.3 (d, C-14), 50.4 (d, C-9), 46.9 (d, C-8), 43.7 (s, C-13), 43.4 (s, C-10), 39.6 (t, C-24), 39.3 (t, C-12), 38.3 (t, C-4), 36.3 (t, C-22), 35.9 (d, C-20), 32.6 (t, C-1), 28.7 (t, C-16), 28.1 (d, C-25), 28.0 (t, C-2), 26.4 (t, C-23), 25.9 (q, C-32, C-33, C-34), 24.0 (t, C-15), 23.0 (q, C-27), 22.7 (q, C-26), 21.8 (t, C-11), 21.4 (q, C-29), 19.0 (q, C-21), 18.2 (s, C-35), 12.4 (q, C-18), –5.5 (q, C-30/31), –5.5 (q, C-30/31).

**FT-IR (ATR):** $\tilde{\nu}$ [cm$^{-1}$] = 2951 (m), 2930 (m), 2859 (m), 1736 (s), 1667 (s), 1470 (w), 1441

| | |
|---|---|
| | (w), 1375 (w), 1361 (w), 1249 (vs), 1283 (w), 1093 (s), 1040 (s), 1005 (w), 912 (w), 890 (w), 836 (vs), 804 (m), 774 (s), 669 (w), 627 (w); 552 (w). |
| **HR-MS:** | (ESI, 70 eV) = *m/z* calcd. for: $C_{35}H_{60}O_4SiNa^+[M+Na]^+$ 595.41531 u, found: 595.41531 u. |
| **$[a]_{\lambda}^{T}$:** | (c = 0.51 g/100 mL, $CHCl_3$ = $[a]_{365}^{20}$: –144.97°, $[a]_{436}^{20}$: –208.86°, $[a]_{546}^{20}$: –116.27°, $[a]_{579}^{20}$: –100.78°, $[a]_{D}^{20}$: –97.58°. |

### 3.4. Synthesis of 19-*tert*-Butyldimethylsilyloxy-cholest-$\Delta^5$-en-3$\beta$-ol (SI-2)

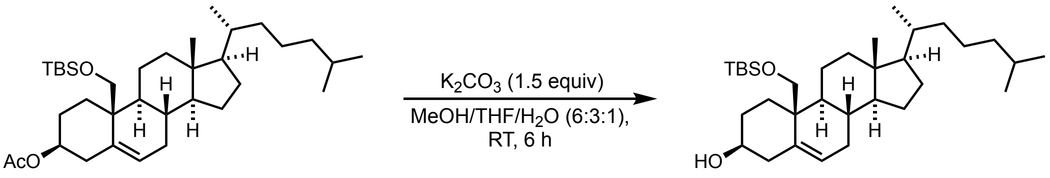


**12** **SI-2**

To a solution of **12** (7.62 g, 13.6 mmol, 1.0 equiv) in a mixture (6:3:1) of MeOH (240 mL), THF (120 mL) and water (40 mL) were added 2.86 g (20.7 mmol, 1.5 equiv) of $K_2CO_3$ and the mixture was stirred at ambient temperature for 6 h. After addition of $NH_4Cl$ (sat. aq.) and extraction with MTBE, the combined organic layers were dried over $MgSO_4$ and the solvent was removed under reduced pressure. The crude product **SI-2** (beige solid) was used in the next step without further purification. An analytical sample of pure **SI-2** was obtained by chromatography.

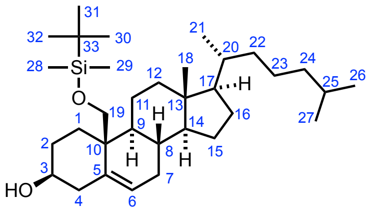


| | |
|---|---|
| **Formula:** | $C_{33}H_{60}O_2Si$ |
| **Molecular Weight:** | 516.93 g/mol |
| **m.p.:** | 50 °C – 52 °C. |
| **TLC:** | $R_f$ = 0.42 (*c*-Hex/EtOAc 2:1), Ce reagent. |
| **$^1$H NMR** | (500 MHz, $CDCl_3$): δ [ppm] = 5.54 (d, *J* = 5.0 Hz, 1H, H-6), 3.73 (d, *J* = 10.5 Hz, 1H, H-19*β*), 3.57 (d, *J* = 10.5 Hz, 1H, H-19*α*), 3.58 – 3.52 (m, 1H, H-3), 2.33 (ddd, *J* = 13.0, 4.9, 2.4 Hz, 1H, H-4*β*), 2.19 – 2.11 (m, 2H, H-1*α*, H-4*α*), 2.01 – 1.93 (m, 2H, H-7, H-12*β*), 1.84 – 1.78 (m, 3H, H-1*β*, H-2*α*, H-16*α*), 1.64 – 0.98 (m, 21H), 0.91 (d, *J* = 6.5 Hz, 3H, H-21), 0.87 – 0.85 (m, 15H, H-26, H-27, H-30, H-31, H-32), 0.70 (s, 3H, H-18), 0.03 (d, *J* = 4.3 Hz, 6H, H-28, H-29). |
| **$^{13}$C NMR** | (125 MHz, $CDCl_3$): δ [ppm] = 136.8 (s, C-5), 125.4 (d, C-6), 71.8 (d, C-3), 63.3 (t, C-19), 57.5 (d, C-17), 56.3 (d, C-14), 50.7 (d, C-9), 42.9 (t, C-4), 42.7 (s, C-13), 41.4 (s, C-10), 40.4 (t, C-12), 39.7 (t, C-24), 36.3 (t, C-22), 35.9 (d, C-20), 32.9 (d, C-8), 32.8 (t, C-1), 32.2 (t, C-2), 31.8 (t, C-7), 28.4 (t, C-16), 28.2 (d, C-25), 26.0 (q, C-30, C-31, C-32), 24.4 (t, C-15), 24.0 (t, C-23), 23.0 (q, C-27), 22.7 (q, C-26), 22.1 (t, C-11), 18.9 (q, C-21), 18.3 (s, C-33), 12.3 (q, C-18), –5.4 (q, C-28/29), –5.5 (q, C-28/29). |

**FT-IR (ATR):** $\tilde{\nu}$ [cm$^{-1}$] = 3336 (bw), 2930 (m), 2900 (m), 2862 (m), 1470 (m), 1441 (w), 1382 (w), 1364 (w), 1250 (m), 1091 (m), 1051 (m), 1018 (w), 1005 (w), 972 (w), 898 (w), 835 (vs), 799 (w), 774 (s), 667 (w), 593 (w).

**HR-MS:** (ESI, 70 eV) = *m/z* calcd. for: $C_{33}H_{60}O_2SiNa^+$[M+Na]$^+$ 539.42548 u, found: 539.42532 u.

**$[a]_{\lambda}^{T}$:** (c = 0.45 g/100 mL, $CHCl_3$) = $[a]_{365}^{20}$: –165.48°, $[a]_{436}^{20}$: –101.63°, $[a]_{546}^{20}$: –57.93°, $[a]_{579}^{20}$: –50.82°, $[a]_{D}^{20}$: –49.48°.

## 3.5. Synthesis of 3*β*-Methoxymethyloxy-19-*tert*-butyldimethylsilyloxy-cholest-Δ$^5$-ene (13)

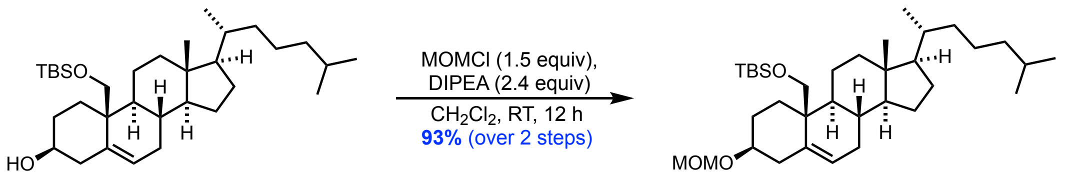


**SI-2** **13**

To a stirred solution of alcohol **SI-2** from the previous step (**3.4.**) in dry $CH_2Cl_2$ (170 mL) were added under an atmosphere of argon 5.53 mL (32.4 mmol, 2.4 equiv) of DIPEA and (10 min later) 1.54 mL (20.2 mmol, 1.5 equiv) of MOMCl. Stirring was continued for 12 h before $NH_4Cl$ (sat. aq.) was added. The mixture was extracted with $CH_2Cl_2$, and the combined organic layers were washed with HCl (1 M, aq.) and brine and dried over $MgSO_4$. After removal of the solvents in vacuo the residue was purified by silica gel column chromatography (*c*-Hex/EtOAc, 5:1) to give **13** (7.13 g, 12.7 mmol, 93% over two steps) as a colorless oil.

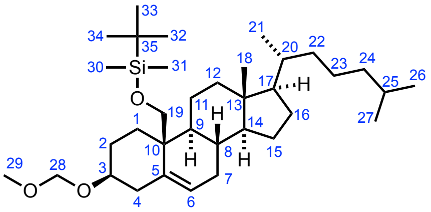

**Formula:** $C_{35}H_{64}O_3Si$

**Molecular Weight:** 560.98 g/mol

**Yield:** 7.13 g (12.7 mmol, 93% over two steps).

**TLC:** $R_f$ = 0.65 (*c*-Hex/EtOAc 2:1), Ce reagent.

**$^1$H NMR** (500 MHz, $CDCl_3$): δ [ppm] = 5.54 (d, *J* = 5.0 Hz, 1H, H-6), 4.68 (s, 2H, H-28), 3.73 (d, *J* = 10.5 Hz, 1H, H-19*β*), 3.58 (d, *J* = 10.5 Hz, 1H, H-19*α*), 3.73 (d, *J* = 10.5 Hz, 1H, H-3), 3.37 (s, 3H, H-29), 2.38 (ddd, *J* = 13.2, 4.9, 2.3 Hz, 1H, H-12*β*), 2.20 (ddd, *J* = 13.8, 10.8, 2.6 Hz, 1H, H-12*α*), 2.13 (dt, *J* = 13.6, 3.6 Hz, 1H, H-1*α*), 2.01 – 1.94 (m, 2H, H-4*β*, H-7), 1.88 – 1.79 (m, 3H, H-1*β*, H-2*α*, H-16*α*), 1.55 – 1.06 (m, 20H), 0.91 (d, *J* = 6.5 Hz, 3H, H-21), 0.88 – 0.85 (m, 15H, H-26, H-27, H-32, H-33, H-34), 0.70 (s, 3H, H-18), 0.03 (d, *J* = 4.3 Hz, 6H, H-30/31).

**$^{13}$C NMR** (125 MHz, $CDCl_3$): δ [ppm] = 136.7 (s, C-5), 125.4 (d, C-6), 94.8 (t, C-28), 76.8 (d, C-3), 63.2 (t, C-19), 57.5 (d, C-17), 56.3 (d, C-14), 55.3 (q, C-29), 50.7 (d, C-9), 42.7 (s, C-13), 41.6 (s, C-10), 40.4 (t, C-4), 40.1 (t, C-12), 39.7 (t, C-24), 36.3 (t, C-22), 35.9 (d, C-20), 32.9 (d, C-8), 32.8 (t, C-1), 31.8 (t, C-

7), 29.5 (t, C-2), 28.4 (t, C-16), 28.2 (d, C-25), 26.0 (q, C-32, C-33, C-34), 24.4 (t, C-15), 24.0 (t, C-23), 23.0 (q, C-27), 22.7 (q, C-26), 22.0 (t, C-11), 18.9 (q, C-21), 18.3 (s, C-35), 12.3 (q, C-18), –5.4 (q, C-30/31), –5.5 (q, C-30/31).

**FT-IR (ATR):** $\tilde{\nu}$ [cm$^{-1}$] = 2929 (s), 2866 (m), 2853 (m), 1471 (m), 1441 (w), 1381 (w), 1364 (w), 1255 (m), 1210 (w), 1148 (m), 1103 (s), 1037 (s), 1005 (m), 957 (w), 939 (w), 917 (w), 836 (vs), 797 (w), 774 (s), 667 (w), 624 (w).

**HR-MS:** (ESI, 70 eV) = *m/z* calcd. for: $C_{35}H_{64}O_3SiNa^+$[M+Na]$^+$ 583.45169 u, found: 583.45154 u.

**$[a]_{\lambda}^{T}$:** (c = 0.48 g/100 mL, $CHCl_3$) = $[a]_{365}^{20}$: –118.33°, $[a]_{436}^{20}$: –71.80°, $[a]_{546}^{20}$: –40.49°, $[a]_{579}^{20}$: –35.14°, $[a]_{D}^{20}$: –34.86°.

## 3.6. Synthesis of 3*β*-Methoxymethyloxy-19-*tert*-butyldimethylsilyloxy-cholest-Δ$^5$-en-7-one (14)

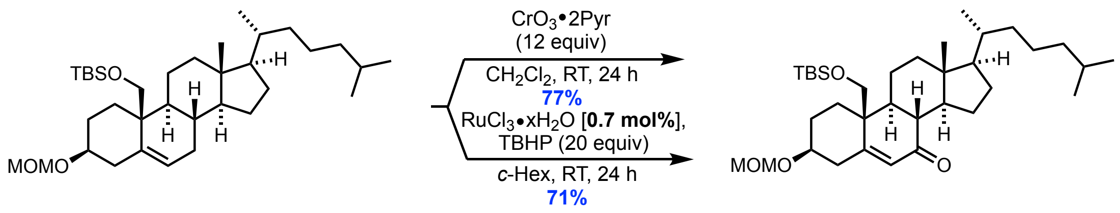


**13** **14**

**Procedure A**

Under argon atmosphere, 11.78 g (117.8 mmol, 9.0 equiv) of $CrO_3$ were added under rapidly stirring to a solution of pyridine (19.02 mL, 235.6 mmol, 18.0 equiv) in dry $CH_2Cl_2$ (350 mL). The mixture (red solution) was kept for 20 min at ambient temperature before as solution of 7.34 g (13.1 mmol, 1.0 equiv) of **13** in $CH_2Cl_2$ (20 mL) was added in one portion. Stirring was continued at ambient temperature for 17 h. Then, an additional portion of the $CrO_3 \cdot$ pyridine complex (prepared from 3.93 g (39.3 mmol, 3.0 equiv) of $CrO_3$ and 6.34 mL (78.5 mmol, 6.0 equiv) of pyridine in 150 mL of $CH_2Cl_2$) was added. After stirring the mixture for further 7 h it was diluted with 500 mL of MTBE and washed with $NaHCO_3$ (sat. aq.), HCl (1 M, aq.), again with $NaHCO_3$ (sat. aq.) and finally with brine. The combined organic layers were dried over $MgSO_4$ and the solvent was removed under reduced pressure. The residue (brown oil) was purified by silica gel column chromatography (*c*-Hex/EtOAc, 5:1) to give enone **14** (5.83 g, 10.1 mmol, 77%) as a white solid.

**Procedure B**

To a solution of 36 mg (0.064 mmol, 1.0 equiv) of **13** in *c*-Hex (0.5 mL) were added 1 mg (0.005 mmol, 0.7 mol%, 0.07 equiv) of $RuCl_3 \cdot H_2O$ (suspension). Then TBHP (0.09 mL, 0.64 mmol, 10.0 equiv) was added dropwise and the resulting black clear solution was stirred at ambient temperature for 8 h before an additional portion of TBHP (0.09 mL, 0.64 mmol, 10.0 equiv) was added and the stirring continued for further 8 h. The reaction mixture was then quenched with $Na_2SO_3$

(sat. aq.). The aqeous layer was extracted with MTBE and the combined organic layers were washed with water, brine and dried over $MgSO_4$. The solvent was removed under reduced pressure and the residue (yellowish oil) was purified by silica gel column chromatography (*c*-Hex/EtOAc, 5:1) to give enone **14** (26 mg, 0.045 mmol, 71%) as a light-yellow oil.

**Formula:** $C_{35}H_{62}O_4Si$

**Molecular Weight:** 574.96 g/mol

**TLC:** $R_f$ = 0.35 (*c*-Hex/EtOAc 3:1), Ce reagent.

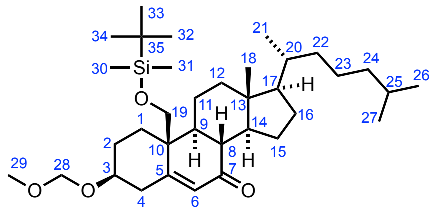


**$^1$H NMR** (500 MHz, $CDCl_3$): δ [ppm] = 5.85 (d, *J* = 1.5 Hz, 1H, H-6), 4.68 (s, 2H, H-28), 3.91 (d, *J* = 10.6 Hz, 1H, H-19*β*), 3.81 (d, *J* = 10.6 Hz, 1H, H-19*α*), 3.60 – 3.54 (m, 1H, H-3), 3.37 (s, 3H, H-29), 2.85 (dd, *J* = 12.6, 10.4 Hz, 1H, H-8), 2.59 (ddd, *J* = 13.7, 4.6, 2.2 Hz, 1H, H-12*β*), 2.38 – 2.32 (m, 2H, H-12*α*, H-23), 2.07 – 1.05 (m, 22H), 0.91 (d, *J* = 6.5 Hz, 3H, H-21), 0.85 (dd, *J* = 6.6, 2.3 Hz, 6H, H-26, H-27), 0.84 (s, 9H, H-32, H-33, H-34), 0.70 (s, 3H, H-18), 0.05 (s, 3H, H-30/31), 0.02 (s, 3H, H-30/31).

**$^{13}$C NMR** (125 MHz, $CDCl_3$): δ [ppm] = 203.1 (s, C-7), 160.7 (s, C-5), 129.2 (d, C-6), 95.1 (t, C-28), 75.7 (d, C-3), 64.1 (t, C-19), 55.5 (q, C-29), 54.9 (d, C-17), 51.4 (d, C-14), 50.5 (d, C-9), 46.9 (d, C-8), 43.6 (s, C-13), 43.5 (s, C-10), 39.9 (t, C-24), 39.6 (t, C-12), 39.4 (t, C-4), 36.3 (t, C-22), 35.9 (d, C-20), 32.9 (t, C-1), 29.2 (t, C-16), 28.8 (t, C-2), 28.1 (d, C-25), 26.4 (t, C-23), 25.9 (q, C-32, C-33, C-34), 24.0 (t, C-15), 23.0 (q, C-27), 22.7 (q, C-26), 21.8 (t, C-11), 19.0 (q, C-21), 18.3 (s, C-35), 12.4 (q, C-18), –5.5 (q, C-30/31), –5.5 (q, C-30/31).

**FT-IR (ATR):** $\tilde{\nu}$ [$cm^{-1}$] = 2948 (s), 2931 (s), 2857 (m), 2951 (w), 1670 (s), 1470 (m), 1444 (w), 1382 (w), 1365 (w), 1300 (w), 1251 (m), 1215 (w), 1149 (w), 1101 (vs), 1036 (vs), 1005 (w), 916 (m), 839 (vs), 776 (s), 734 (vs), 668 (w), 648 (w), 626 (w).

**HR-MS:** (ESI, 70 eV) = *m/z* calcd. for: $C_{35}H_{62}O_4SiNa^+$[M+Na]$^+$ 597.43096 u, found: 597.43088 u.

**$[a]_\lambda^T$:** (c = 0.51 g/100 mL, $CHCl_3$ = $[a]^{20}_{436}$: –221.57°, $[a]^{20}_{546}$: –128.63°, $[a]^{20}_{579}$: –112.55°, $[a]^{20}_{D}$: –109.80°.

### 3.7. Synthesis of 3*β*-Methoxymethyloxy-7-(*para*-toluenesulfonyl)-hydrazonyl-19-*tert*-butyldimethylsilyloxy-cholest-$\Delta^5$-ene (SI-3)

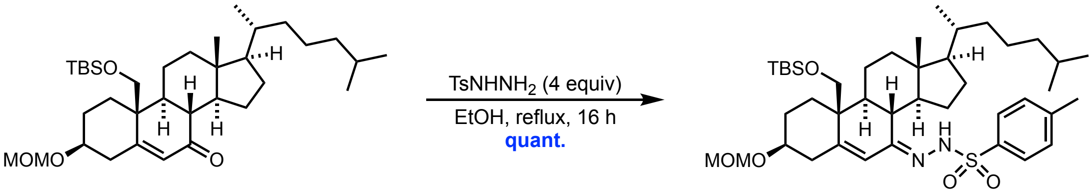


**14** *syn/anti*-**SI-3**

To a solution of 9.04 g (15.7 mmol, 1.0 equiv) of enone **14** in EtOH (350 mL) were added 11.7 g (62.8 mmol, 4.0 equiv) of *p*-toluenesulfonyl hydrazide ($TsNHNH_2$) and the reaction mixture was heated to reflux for 16 h. After cooling to ambient temperature the solvent was removed under reduced pressure and the residue (yellow solid) was immediately purified by silica gel column chromatography (*c*-Hex/EtOAc, 5:1) to give hydrazone **SI-3** (mixture of *syn*/*anti* isomers, 11.2 g, 15.7 mmol, quant.) as a beige foam, which was used subsequently in the next step. Note: It is very important to purify hydrazone **SI-3** because otherwise a complicated product mixture is formed in the following elimination step (3.8.).

**Formula:** $C_{42}H_{40}N_2O_5SSi$

**Molecular Weight:** 743.18 g/mol

**Yield:** 11.23 g (15.71 mmol, quant).

**m.p.:** 69 °C – 72 °C.

**TLC:** $R_f$ = 0.37 (*c*-Hex/EtOAc 2:1), Ce reagent.

**FT-IR (ATR):** $\tilde{\nu}$ [cm$^{-1}$] = 3209 (bw), 2949 (m), 2930 (m), 2883 (m), 2861 (m), 1741 (w), 1639 (w), 1601 (w), 1470 (w), 1444 (w), 1380 (w), 1339 (w), 1307 (w), 1251 (w), 1214 (w), 1165 (vs), 1094 (vs), 1034 (vs), 969 (w), 933 (w), 898 (m), 837 (vs), 812 (m), 775 (m), 713 (m), 668 (s), 594 (m), 547 (s).

**HR-MS:** (ESI, 70 eV) = *m/z* calcd. for: $C_{42}H_{40}N_2O_5SSi^+$ $[M+H]^+$ 743.48475 u, found: 743.48493 u.

**$[a]_\lambda^T$:** (c = 0.70 g/100 mL, $CHCl_3$) = $[a]_{436}^{20}$: –358.19°, $[a]_{546}^{20}$: –177.14°, $[a]_{579}^{20}$: –151.14°, $[a]_D^{20}$: –145.52°.

### 3.8. Synthesis of 3*β*-Methoxymethyloxy-19-*tert*-butyldimethylsilyloxy-cholest-$\Delta^{5,7}$-diene (15)

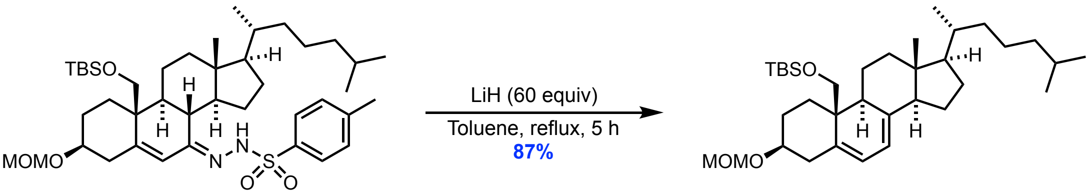


*syn/anti*-**SI-3** **15**

To a solution of 6.51 g (8.76 mmol, 1.0 equiv) of hydrazone **SI-3** in toluene (200 mL) were added

4.18 g (526 mmol, 60.0 equiv) of LiH and the mixture was heated to reflux for 5 h. Then, the reaction mixture was cooled to ambient temperature and the white residue (excess of LiH) was removed by means of filtration. The filtrate was concentrated to 30 mL and diluted with EtOAc and water. The aqueous layer was extracted with EtOAc, the combined organic layers were washed with brine and dried over $MgSO_4$. The solvent was removed under reduced pressure and the residue was purified by silica gel column chromatography (*c*-Hex/EtOAc, 20:1) to give diene **15** (4.24 g, 7.58 mmol, 87%) as a yellowish oil.

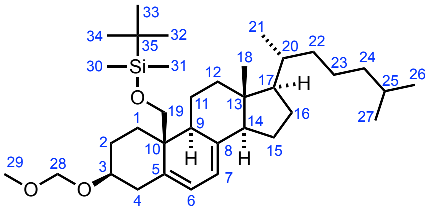


**Formula:** $C_{35}H_{62}O_3Si$

**Molecular Weight:** 558.96 g/mol

**Yield:** 4.24 g (7.58 mmol, 87%).

**TLC:** $R_f$ = 0.76 (*c*-Hex/EtOAc 2:1), Ce reagent.

**$^{1}$H NMR** (500 MHz, $CDCl_3$): δ [ppm] = 5.66 (dd, *J* = 5.6, 2.0 Hz, 1H, H-6), 5.31 (d, *J* = 5.4 Hz, 1H, H-7), 4.70 (d, *J* = 2.2 Hz, 2H, H-28), 3.72 (d, *J* = 10.6 Hz, 1H, H-19*β*), 3.58 – 3.51 (m, 1H, H-3), 3.46 (d, *J* = 10.6 Hz, 1H, H-19*α*), 3.37 (s, 3H, H-29), 2.51 (ddd, *J* = 14.5, 4.8, 2.4 Hz, 1H, H-4*β*), 2.38 (dt, *J* = 13.7, 3.7 Hz, 1H, H-1*α*), 2.30 – 2.25 (m, 1H, H-4*α*), 2.09 – 0.98 (m, 22H), 0.93 (d, *J* = 6.5 Hz, 3H, H-21), 0.87 (s, 15H, H-26, H-27, H-32, H-33, H-34), 0.63 (s, 3H, H-18), 0.01 (d, *J* = 6.8 Hz, 6H, H-30/31).

**$^{13}$C NMR** (125 MHz, $CDCl_3$): δ [ppm] = 142.4 (s, C-5), 135.5 (s, C-8), 122.5 (d, C-6), 115.9 (d, C-7), 94.9 (t, C-28), 75.7 (d, C-3), 61.6 (t, C-19), 56.1 (d, C-17), 55.4 (q, C-29), 54.8 (d, C-14), 47.0 (d, C-9), 43.3 (s, C-13), 42.2 (s, C-10), 39.9 (t, C-24), 39.6 (t, C-12), 38.9 (t, C-4), 36.3 (d, C-20), 36.3 (t, C-22), 33.3 (t, C-1), 29.6 (t, C-16), 28.2 (d, C-25), 28.2 (t, C-2), 26.0 (q, C-32, C-33, C-34), 24.0 (t, C-23), 23.1 (t, C-15), 23.0 (q, C-27), 22.7 (q, C-26), 22.7 (t, C-11), 19.0 (q, C-21), 18.4 (s, C-35), 12.4 (q, C-18), –5.3 (q, C-30/31), –5.6 (q, C-30/31).

**FT-IR (ATR):** $\tilde{\nu}$ [$cm^{-1}$] = 2948 (m), 2930 (m), 2868 (m), 2854 (m), 1470 (m), 1445 (w), 1380 (w), 1362 (w), 1255 (m), 1212 (w), 1149 (m), 1103 (s), 1084 (s), 1042 (s), 1007 (w), 954 (w), 916 (w), 836 (vs), 775 (s), 735 (m), 667 (w), 588 (w).

**HR-MS:** (ESI, 70 eV) = *m/z* calcd. for: $C_{35}H_{62}O_3SiNa^+$[M+Na]$^+$ 581.43604 u, found: 581.43646 u.

**$[a]_{\lambda}^{T}$:** (c = 0.69 g/100 mL, $CHCl_3$) = $[a]_{365}^{20}$: –473.33°, $[a]_{436}^{20}$: –186.57°, $[a]_{546}^{20}$: –76.33°, $[a]_{579}^{20}$: –62.85°, $[a]_{D}^{20}$: –59.47°.

### 3.9. Synthesis of 3*β*-Methoxymethyloxy-5*α*-hydroxy-19-*tert*-butyldimethylsilyloxy-cholest-$\Delta^7$-en-6-one (SI-4)

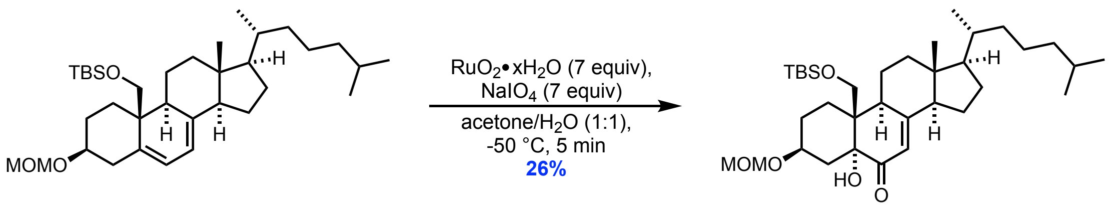


**15** **SI-4**

92 mg (0.16 mmol, 1.0 equiv) of diene **15** were dissolved in acetone (40 mL) and cooled to -50 °C. In the meantime, 30 mg (0.17 mmol, 7.0 equiv) of $RuO_2 \cdot xH_2O$ and 235 mg (1.10 mmol, 7.0 equiv) of $NaIO_4$ were dissolved in 6 mL acetone/water (1:1) in a separate flask and stirred for 1 h. The yellowish, cloudy solution was centrifuged, and the yellowish, clear solution was cooled to -50 °C and added to the substrate solution. The reaction mixture was stirred for 5 min (no starting material left according to TLC), quenched by addition of *i*-PrOH and filtrated through a short pad of Celite®. The filtrate was extracted with MTBE and the combined organic layers were washed with water (3x), brine and dried over $MgSO_4$. The residue was purified by silica gel column chromatography (*c*-Hex/EtOAc, 5:1) to give enone **SI-4** (25 mg, 0.04 mmol, 26%) as a white foam.

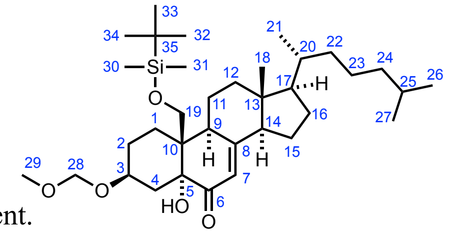

**Formula:** $C_{35}H_{62}O_5Si$

**Molecular Weight:** 590.96 g/mol

**Yield:** 25 mg (0.04 mmol, 26%).

**m.p.:** 57 °C – 59 °C.

**TLC:** $R_f$ = 0.31 (*c*-Hex/EtOAc 2:1), Ce reagent.

**$^1$H NMR** (600 MHz, $CDCl_3$): δ [ppm] = 5.61 (s, 1H, H-7), 4.71 (d, $J$ = 6.9 Hz, 1H, H-28), 4.67 (d, $J$ = 6.9 Hz, 1H, H-28), 3.99 – 3.94 (m, 1H, H-3), 3.80 (d, $J$ = 11.0 Hz, 1H, H-19*β*), 3.58 (d, $J$ = 11.0 Hz, 1H, H-19*α*), 3.38 (s, 3H, H-29), 2.37 (ddd, $J$ = 12.5, 6.4, 2.4 Hz, 1H, H-9), 2.23 (d, $J$ = 14.3, 5.0, 1.8 Hz, 1H, H-1*α*), 2.13 – 2.07 (m, 3H, H-4*β*, H-12*β*, H-14), 1.88 – 1.20 (m, 22H), 0.93 (d, $J$ = 6.4 Hz, 3H, H-21), 0.86 (dd, $J$ = 6.6, 2.9 Hz, 6H, H-26, H-27), 0.84 (s, 9H, H-32, H-33, H-34), 0.65 (s, 3H, H-18), 0.00 (s, 3H, H-30/31), –0.03 (s, 3H, H-30/31).

**$^{13}$C NMR** (150 MHz, $CDCl_3$): δ [ppm] = 197.4 (s, C-6), 165.1 (s, C-8), 120.4 (d, C-7), 94.8 (t, C-28), 76.4 (s, C-5), 72.7 (d, C-3), 63.0 (d, C-19), 56.5 (d, C-17), 56.2 (d, C-14), 55.4 (q, C-29), 45.7 (s, C-13), 45.4 (s, C-10), 44.9 (d, C-9), 39.7 (t, C-4), 39.6 (t, C-12, C-24), 36.1 (d, C-20), 36.1 (t, C-22), 33.6 (t, C-1), 28.3 (t, C-16), 28.1 (d, C-25), 27.9 (t, C-2), 26.4 (t, C-23), 26.1 (q, C-32, C-33, C-34), 24.1 (t, C-15), 23.0 (q, C-27), 22.7 (q, C-26), 22.7 (t, C-11), 19.0 (q, C-21), 18.4 (s, C-35), 13.1 (q, C-18), –5.6 (q, C-30/31), –6.0 (q, C-30/31).

| | |
|---|---|
| **FT-IR (ATR):** | $\tilde{\nu}$ [cm$^{-1}$] = 3402 (bw), 2950 (m), 2929 (m), 2860 (m), 1714 (w), 1679 (m), 1626 (w), 1469 (m), 1447 (w), 1377 (w), 1365 (w), 1251 (m), 1214 (w), 1146 (m), 1099 (s), 1041 (s), 1005 (w), 965 (w), 950 (w), 916 (w), 836 (vs), 775 (s), 669 (w), 612 (w), 546 (w). |
| **HR-MS:** | (ESI, 70 eV) = *m/z* calcd. for: $C_{35}H_{62}O_5SiNa^+[M+Na]^+$ 613.4259 u, found: 613.4252 u. |
| **$[a]_\lambda^T$:** | (c = 0.52 g/100 mL, $CHCl_3$) = $[a]_{436}^{20}$: +163.71°, $[a]_{546}^{20}$: +45.38°, $[a]_{579}^{20}$: +35.38°, $[a]_D^{20}$: +32.95°. |

### 3.10. Synthesis of 3*β*-Methoxy-19-hydroxy-cholest-$\Delta^{5,7}$-diene (SI-5)

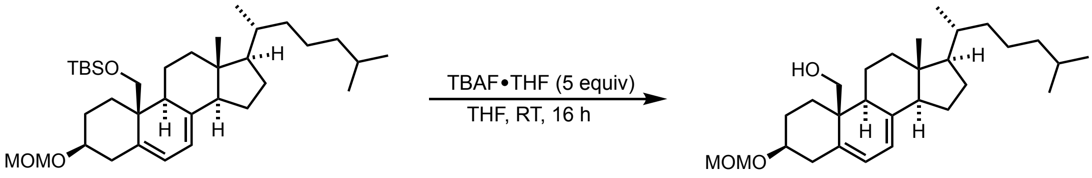


**15** **SI-5**

1.98 g (3.54 mmol, 1.0 equiv) of diene **15** were dissolved in dry THF (10 mL) under argon atmosphere. To this solution, 17.71 mL (17.71 mmol, 5.0 equiv) of TBAF · THF (1 M) were added and the reaction mixture was stirred at ambient temperature for 16 h. The reaction was stopped by addition of water, and the solution was diluted with EtOAc. The aqueous layer was extracted with EtOAc and the combined organic layers were washed with water, brine and dried over $MgSO_4$. The solvent was removed under reduced pressure and the crude product **SI-5** was used in the next step without any further purification. Only a small analytical sample of **SI-5** was purified by chromatography.

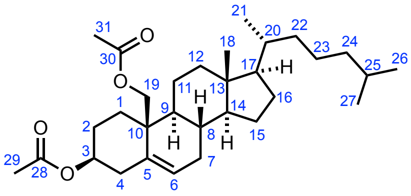

| | |
|---|---|
| **Formula:** | $C_{29}H_{48}O_3$ |
| **Molecular Weight:** | 444.70 g/mol |
| **m.p.:** | 89 °C – 92 °C. |
| **TLC:** | $R_f$ = 0.41 (*c*-Hex/EtOAc 2:1), Ce reagent. |
| **$^1$H NMR** | (600 MHz, $CDCl_3$): δ [ppm] = 5.86 (dd, *J* = 5.7, 2.0 Hz, 1H, H-6), 5.28 (d, *J* = 5.7 Hz, 1H, H-7), 4.69 (s, 2H, H-28), 3.79 (dd, *J* = 11.3, 2.6 Hz, 1H, H-19*β*), 3.63 – 3.55 (m, 2H, H-3, H-19*α*), 3.37 (s, 3H, H-29), 2.54 (ddd, *J* = 14.2, 4.7, 2.1 Hz, 1H, H-4*β*), 2.41 (t, *J* = 12.2 Hz, 1H, H-4*α*), 2.14 (dt, *J* = 12.6, 3.4 Hz, 2H, H-9, H-12*β*), 2.06 – 1.99 (m, 2H, H-1, H-15), 1.95 – 1.83 (m, 3H, H-2*α*, H-14, H-16*α*), 1.70 – 1.10 (m, 17H), 0.93 (d, *J* = 6.5 Hz, 3H, H-21), 0.86 (dd, *J* = 6.6, 2.7 Hz, 6H, H-26, H-27), 0.67 (s, 3H, H-18). |
| **$^{13}$C NMR** | (150 MHz, $CDCl_3$): δ [ppm] = 144.0 (s, C-5), 133.6 (s, C-8), 123.3 (d, C-6), 115.0 (d, C-7), 95.0 (t, C-28), 75.7 (d, C-3), 65.0 (t, C-19), 56.1 (d, C-17), 55.4 (q, C-29), 55.2 (d, C-14), 46.2 (d, C-9), 43.7 (s, C-13), 42.3 (s, C-10), 39.7 (t, C-24), 39.6 (t, C-12), 38.5 (t, C-4), 36.2 (t, C-22), 36.2 (d, C-20), 35.8 (t, C-1), |

| | |
|---|---|
| | 29.7 (t, C-16), 28.2 (d, C-25), 28.1 (t, C-2), 24.0 (t, C-23), 23.0 (q, C-27), 22.9 (t, C-15), 22.7 (q, C-26), 22.0 (t, C-11), 19.0 (q, C-21), 12.2 (q, C-18). |
| **FT-IR (ATR):** | $\tilde{\nu}$ [cm$^{-1}$] = 3440 (bw), 3366 (br w), 2950 (m), 2935 (m), 2869 (m), 2850 (m), 2779 (w), 1653 (w), 1601 (w), 1467 (w), 1443 (w), 1375 (w), 1344 (w), 1323 (w), 1258 (w), 1214 (w), 1147 (m), 1106 (s), 1046 (vs), 1027 (vs), 997 (w), 962 (w), 943 (w), 931 (w), 912 (w), 897 (m), 834 (m), 803 (w), 761 (w), 701 (w), 668 (w). |
| **HR-MS:** | (ESI, 70 eV) = *m/z* calcd. for: $C_{29}H_{48}O_3Na^+$[M+Na]$^+$ 467.34957 u, found: 467.34945 u. |
| **$[a]_\lambda^T$:** | (c = 0.56 g/100 mL, $CHCl_3$) = $[a]_{436}^{20}$: –164.17°, $[a]_{546}^{20}$: –50.36°, $[a]_{579}^{20}$: –38.63°, $[a]_D^{20}$: –36.67°. |

### 3.11. Synthesis of 3*β*-Methoxymethyloxy-19-acetoxy-cholest-$\Delta^{5,7}$-diene (16)

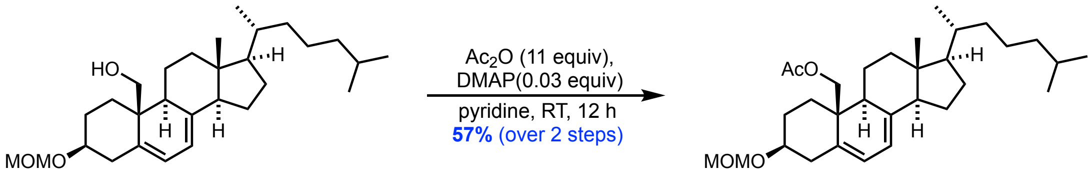


The crude alcohol **SI-5** from the previous step (3.10.) was dissolved in pyridine (8 mL). To this solution, 2.77 mL (29.3 mmol, 11 equiv) of $Ac_2O$ and 9 mg (0.07 mmol, 0.03 equiv) of DMAP were added and the reaction mixture was stirred at ambient temperature for 12 h. After completion of the reaction (TLC control), the mixture was diluted with water and extracted with EtOAc. The combined organic layers were washed with HCl (1 M, aq.), $NaHCO_3$ (sat. aq.), brine and dried over $MgSO_4$. The solvent was removed under reduced pressure and the residue (yellowish oil) was purified by silica gel column chromatography (*c*-Hex/EtOAc, 10:1) to give **16** (990 mg, 2.03 mmol, 57% over two steps) as a light yellow solid.

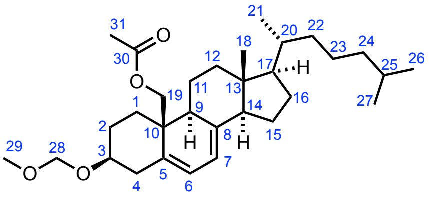

| | |
|---|---|
| **Formula:** | $C_{31}H_{50}O_4$ |
| **Molecular Weight:** | 486.74 g/mol |
| **Yield:** | 990 mg (2.03 mmol, 57% over two steps). |
| **m.p.:** | 87 °C – 90 °C. |
| **TLC:** | $R_f$ = 0.51 (*c*-Hex/EtOAc 2:1), Ce reagent. |
| **$^1$H NMR** | (500 MHz, $CDCl_3$): δ [ppm] = 5.70 (dd, *J* = 5.7, 2.1 Hz, 1H, H-6), 5.30 (d, *J* = 5.7 Hz, 1H, H-7), 4.68 (s, 2H, H-28), 4.56 (d, *J* = 11.6 Hz, H-19*β*), 3.87 (d, *J* = 11.6 Hz, 1H, H-19*α*), 3.58 – 3.52 (m, 1H, H-4), 3.36 (s, 3H, H-29), 2.51 (ddd, *J* = 14.4, 5.0, 2.3 Hz, 1H, H-4*β*), 2.42 (ddd, *J* = 14.1, 11.7, 2.1 Hz, 1H, H-4*α*), 2.13 – 2.07 (m, 3H, H-1*α*, H-9, H-12*β*), 1.98 (s, 3H, H-31), 1.74 – 1.08 (m, 20H), 0.93 (d, *J* = 6.5 Hz, 3H, H-21), 0.86 (dd, *J* = 6.6, 2.2 Hz, 6H, H-26, |

H-27), 0.64 (s, 3H, H-18).

**$^{13}$C NMR** (125 MHz, $CDCl_3$): δ [ppm] = 171.0 (s, C-30), 142.1 (s, C-5), 133.5 (s, C-8), 123.2 (d, C-6), 115.7 (d, C-7), 94.9 (t, C-28), 75.6 (d, C-3), 65.0 (t, C-19), 56.0 (d, C-17), 55.4 (q, C-29), 55.0 (d, C-14), 46.4 (d, C-9), 43.4 (s, C-13), 40.5 (s, C-10), 39.6 (t, C-12/24), 39.6 (t, C-12/24) 38.7 (t, C-4), 36.3 (d, C-20), 36.2 (t, C-22), 35.3 (t, C-1), 29.6 (t, C-16), 28.2 (t, C-2) 28.2 (d, C-25), 24.0 (t, C-23), 23.0 (t, C-15), 23.0 (q, C-27), 22.7 (q, C-26), 22.4 (t, C-11), 21.2 (q, C-31), 19.0 (q, C-21), 12.0 (q, C-18).

**FT-IR (ATR):** $\tilde{\nu}$ [cm$^{-1}$] = 2946 (m), 2935 (m), 2870 (w), 1743 (m), 1467 (w), 1446 (w), 1377 (w), 1366 (w), 1225 (s), 1148 (m), 1105 (m), 1034 (vs), 971 (w), 934 (w), 916 (m), 840 (w), 804 (w), 603 (w).

**HR-MS:** (ESI, 70 eV) = *m/z* calcd. for: $C_{31}H_{50}O_4Na^+[M+Na]^+$ 509.36068 u, found: 509.36013 u.

**$[a]_\lambda^T$:** (c = 0.61 g/100 mL, $CHCl_3$) = $[a]_{365}^{20}$: –616.31°, $[a]_{436}^{20}$: –263.88°, $[a]_{546}^{20}$: –112.95°, $[a]_{579}^{20}$: –94.10°, $[a]_{D}^{20}$: –89.95°.

### 3.12. Synthesis of 3*β*-Methoxymethyloxy-5*α*-hydroxy-19-acetoxy-cholest-Δ$^7$-en-6-one (17)

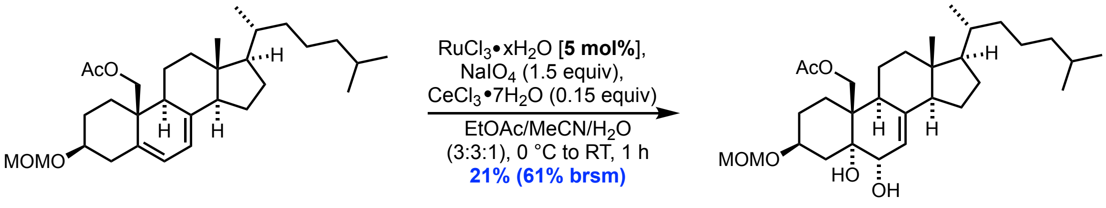


**16** **SI-6**

571 mg (2.67 mmol, 1.5 equiv) of $NaIO_4$ and 99 mg (0.27 mmol, 0.15 equiv) of $CeCl_3 \cdot 7H_2O$ were dissolved in water (3 mL). The beige suspension was heated gently (60 °C) until the color changed to yellow. After cooling the suspension to 0 °C, MeCN (11.4 mL) and EtOAc (7.6 mL) were added, followed by 22 mg (0.09 mmol, 5 mol%, 0.05 eq.) of $RuCl_3 \cdot xH_2O$ in water (0.8 mL). The brownish solution was stirred for 2 min, before adding 867 mg (1.78 mmol, 1.0 equiv) of diene **16** in EtOAc (3.8 mL) in one portion. After 1 h, solid $Na_2SO_4$ was added to the reaction mixture and the residue was removed by filtration. The filter cake was washed with EtOAc and the filtrate was diluted with water. Then the aqueous layer was extracted with EtOAc. The combined organic layers were washed with $Na_2SO_3$ (sat. aq.), brine and dried over $MgSO_4$. The solvent was removed under reduced pressure and the residue was purified by silica gel column chromatography (*c*-Hex/EtOAc, 3:1) to give 346 mg of recovered **16** and **SI-6** (192 mg, 0.37 mmol, 21%, 61% brsm) as a white solid.

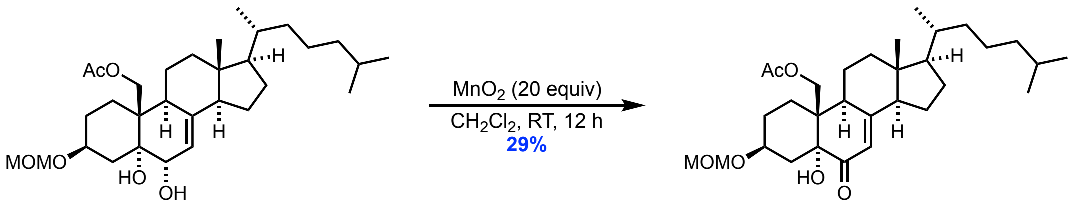


**SI-6** **17**

192 mg (0.37 mmol, 1.0 equiv) of **SI-6** were dissolved in $CH_2Cl_2$ (10 mL). To this solution, 322 mg (3.70 mmol, 10.0 equiv) of $MnO_2$ were added and the reaction mixture was stirred at ambient temperature for 6 h. Another portion of $MnO_2$ (322 mg, 3.70 mmol, 10.0 equiv) was added due to incomplete conversion of the starting material and the reaction mixture was stirred for further 6 h. The excess of $MnO_2$ was removed by filtration and the filtrate was concentrated under reduced pressure. The residue was purified by silica gel column chromatography (*c*-Hex/EtOAc, 2:1) to give **17** (55 mg, 0.11 mmol, 29%) as a white solid.

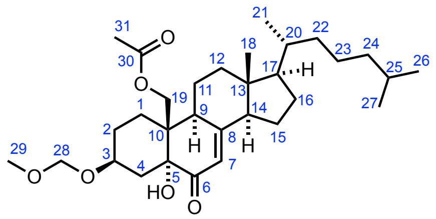

**Formula:** $C_{31}H_{50}O_6$

**Molecular Weight:** 518.74 g/mol

**Yield:** 55 mg (0.11 mmol, 29%).

**m.p.:** 45 °C – 48 °C.

**TLC:** $R_f$ = 0.20 (*c*-Hex/EtOAc 2:1), Ce reagent.

**$^1$H NMR** (500 MHz, $CDCl_3$): δ [ppm] = 5.65 (s, 1H, H-7), 4.65 (dd, *J* = 24.0, 6.9 Hz, 2H, H-28), 4.40 (d, *J* = 12.6 Hz, 1H, H-19*β*), 3.99 (d, *J* = 12.6 Hz, 1H, H-19*α*), 3.97 – 3.92 (m, 1H, H-3), 3.35 (s, 3H, H-29), 2.83 (s, 1H, OH), 2.41 (ddd, *J* = 12.8, 6.3, 2.5 Hz, 1H, H-9), 2.25 (ddd, *J* = 14.5, 5.1 Hz, 1H, H-22), 2.14 (dt, *J* = 12.9, 3.6 Hz, 1H, H-4*β*), 2.10 – 2.07 (m, 1H, H-14), 1.90 (s, 3H, H-31), 1.79 – 1.01 (m, 21H), 0.92 (d, *J* = 6.3 Hz, 3H, H-21), 0.85 (dd, *J* = 6.6 Hz, 6H, H-26, H-27), 0.60 (s, 3H, H-18).

**$^{13}$C NMR** (125 MHz, $CDCl_3$): δ [ppm] = 197.5 (s, C-6), 170.4 (s, C-30), 164.6 (s, C-8), 120.2 (d, C-7), 94.8 (t, C-28), 75.8 (s, C-5), 72.5 (d, C-3), 64.2 (t, C-19), 56.5 (d, C-17), 56.1 (d, C-14), 55.3 (q, C-29), 45.2 (s, C-13), 44.2 (d, C-9), 43.9 (s, C-10), 39.5 (t, C-24), 39.3 (t, C-4), 36.1 (d, C-20), 36.1 (t, C-12), 33.0 (t, C-22), 28.1 (d, C-25), 27.9 (t, C-1), 27.8 (t, C-16), 27.2 (t, C-2), 24.1 (t, C-23), 22.9 (q, C-27), 22.7 (q, C-26), 22.6 (t, C-15), 22.5 (t, C-11), 20.8 (q, C-31), 18.9 (q, C-21), 12.4 (q, C-18).

**FT-IR (ATR):** $\tilde{\nu}$ [cm$^{-1}$] = 3410 (bw), 2951 (m), 2928 (m), 2870 (m), 1745 (m), 1678 (m), 1629 (w), 1466 (m), 1449 (m), 1377 (m), 1366 (m), 1230 (s), 1146 (m), 1102 (m), 1037 (vs), 975 (w), 950 (w), 915 (w), 869 (w), 805 (w), 767 (w), 604 (w).

**HR-MS:** (ESI, 70 eV) = *m/z* calcd. for: $C_{31}H_{50}O_6Na^+$[M+Na]$^+$ 541.34996 u, found: 541.34969 u.

**$[a]_\lambda^T$:** (c = 1.40 g/100 mL, $CHCl_3$) = $[a]_{436}^{20}$: +153.48°, $[a]_{546}^{20}$: +44.74°, $[a]_{579}^{20}$: +35.41°,

$[a]_D^{20}$: –32.83°.

### 3.13. Synthesis of 3*β*-Methoxymethyloxy-5*α*-hydroxy-8*β*,19-epoxy-cholestan-6-one (18)

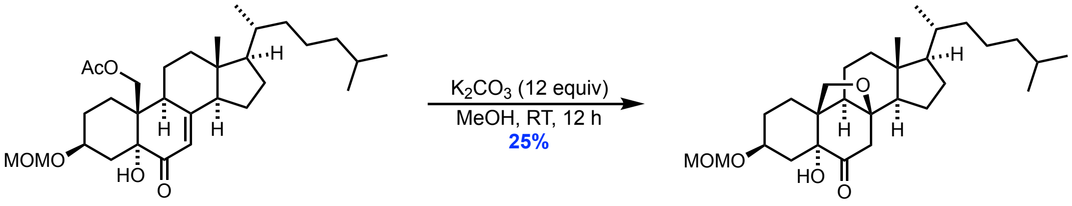


**17** **18**

To a solution of 48 mg (0.09 mmol, 1.0 equiv) of enone **17** in MeOH (5 mL) were added 153 mg (1.11 mmol, 12.0 equiv) of $K_2CO_3$ and the reaction mixture was stirred at ambient temperature for 12 h. After completion of the reaction, the reaction mixture was quenched by the addition of water. After extraction with EtOAc, the combined organic layers were washed with brine, dried over $MgSO_4$ and the solvent was removed under reduced pressure. The residue was purified by silica gel column chromatography (*c*-Hex/EtOAc, 3:1) to give **18** (11 mg, 0.02 mmol, 25%) as a white solid.

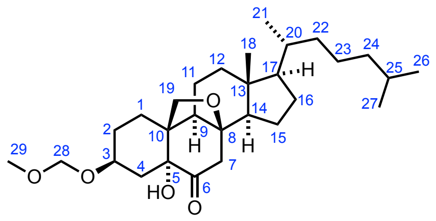

**Formula:** $C_{29}H_{48}O_5$

**Molecular Weight:** 476.70 g/mol

**Yield:** 11 mg (0.02 mmol, 25%).

**m.p.:** 178 °C – 181 °C.

**TLC:** $R_f$ = 0.46 (*c*-Hex/EtOAc 1:1), Ce reagent.

**$^1$H NMR** (500 MHz, $CDCl_3$): δ [ppm] = 4.67 (d, *J* = 1.0 Hz, 2H, H-28), 3.87 – 3.79 (m, 1H, H-3), 3.80 (d, *J* = 9.6 Hz, 1H, H-19*β*), 3.74 (d, *J* = 9.6 Hz, 1H, H-19*α*,*β*), 3.37 (s, 3H, H-29), 2.62 (d, *J* = 2.8 Hz, 2H, H-7), 2.45 (dd, *J* = 12.1, 6.5 Hz, 1H, H-9), 2.31 (ddd, *J* = 14.6, 4.5, 2.1 Hz, 1H, H-4*β*), 2.24 (s, 1H, OH), 2.08 (dt, *J* = 12.9, 3.5 Hz, 1H, H-12*β*), 1.99 – 1.83 (m, 3H, H-1*α*, H-2*α*, H-16*α*), 1.72 – 1.64 (m, 2H, H-2*β*, H-4*α*), 1.54 – 1.04 (m, 17H), 0.91 (d, *J* = 6.5 Hz, 3H, H-21), 0.86 (dd, *J* = 6.6, 2.3 Hz, 6H, H-26, H-27), 0.81 (s, 3H, H-18).

**$^{13}$C NMR** (125 MHz, $CDCl_3$): δ [ppm] = 208.2 (s, C-6), 94.9 (t, C-28), 84.6 (s, C-8), 78.6 (s, C-5), 72.3 (d, C-3), 72.3 (t, C-19), 56.6 (d, C-17), 55.4 (q, C-29), 53.6 (d, C-14), 50.9 (t, C-7), 50.8 (s, C-13), 44.9 (d, C-9), 42.0 (s, C-10), 39.6 (t, C-24), 38.6 (t, C-12), 36.0 (t, C-22), 35.9 (t, C-4), 35.4 (d, C-20), 30.4 (t, C-1), 28.2 (d, C-25), 27.7 (t, C-16), 24.5 (t, C-2), 23.9 (t, C-23), 23.0 (q, C-27), 22.7 (q, C-26), 20.9 (t, C-15), 20.6 (t, C-11), 18.8 (q, C-21), 12.0 (q, C-18).

**FT-IR (ATR):** $\tilde{\nu}$ [cm$^{-1}$] = 3388 (bw), 2947 (s), 2926 (vs), 2867 (s), 2854 (s), 1717 (m), 1666 (w), 1465 (m), 1376 (m), 1304 (w), 1244 (m), 1147 (m), 1105 (m), 1040 (vs), 969 (w), 940 (w), 917 (w), 815 (w), 757 (w), 610 (w).

**HR-MS:** (ESI, 70 eV) = *m/z* calcd. for: $C_{29}H_{48}O_5Na^+$[M+Na]$^+$ 499.33940 u, found:

499.33975 u.

**[a]$^T_\lambda$:** (c = 0.55 g/100 mL, $CHCl_3$) = [a]$^{20}_{436}$: –81.15°, [a]$^{20}_{546}$: –31.09°, [a]$^{20}_{579}$: –25.94°, [a]$^{20}_{D}$: –25.27°.

### 3.14. Synthesis of 3*β*-Hydroxy-8*β*,19-epoxy-cholestan-6-one (4)

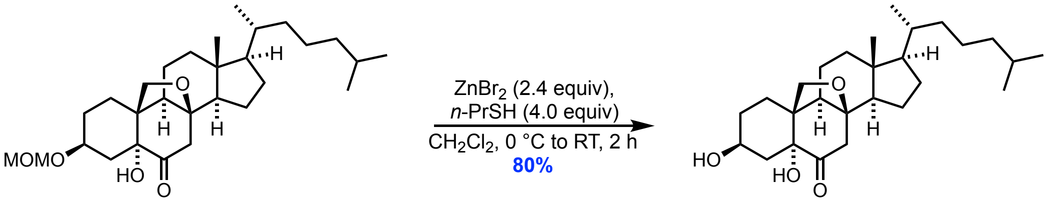


**18** **4**

To a stirred solution of 11 mg (0.23 mmol, 1.0 equiv) of **18** in $CH_2Cl_2$ (0.4 mL) were added 6 mg (0.03 mmol, 1.2 equiv) of $ZnBr_2$ and 4 µL (0.05 mmol, 2.0 equiv) of *n*-PrSH were added at 0 °C. The mixture was allowed to warm to ambient temperature. After 1 h an additional portion of $ZnBr_2$ (6 mg, 0.03 mmol, 1.2 equiv) and *n*-PrSH (4 µL, 0.05 mmol, 2.0 equiv) were added at 0 °C and stirring was continued for further 1 h. After completion (TLC control) the mixture was diluted with $CH_2Cl_2$ and quenched with $NaHCO_3$ (sat. aq.). The aqueous layer was extracted with $CH_2Cl_2$. The combined organic layers were washed with brine, dried over $MgSO_4$ and the solvent was removed under reduced pressure. The residue was purified by silica gel column chromatography (*c*-Hex/EtOAc, 1:1) to give **4** (8 mg, 0.02 mmol, 80%) as a white solid.

**Formula:** $C_{27}H_{44}O_4$

**Molecular Weight:** 432.65 g/mol

**Yield:** 8 mg (0.02 mmol, 80%).

➔ The analytical data were identical with those of a sample of **4** obtained from **21** (see **3.21.** page 30).

### 3.15. Synthesis of 3*β*,19-Diacetoxy-cholest-Δ$^5$-ene (19)[3]

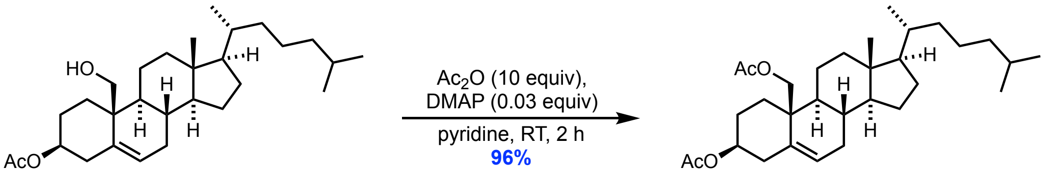


**8** **19**

To a solution of 3.0 g (6.75 mmol, 1.0 equiv) of alcohol **8** (from experiment 3.1.) in pyridine (8 mL) were added 6.56 mL (6.89 g, 67.5 mmol, 10.0 equiv) of $Ac_2O$ followed by 21 mg (0.17 mmol, 0.03 equiv) of DMAP. The mixture was stirred at ambient temperature for 2 h. After completion of the reaction (TLC control), water and EtOAc were added and the layers were separated. The aqueous

layer was extracted with EtOAc and the combined organic layers were washed with HCl (1 M, aq.), $NaHCO_3$ (sat. aq.), brine and dried over $MgSO_4$. The solvent was removed under reduced pressure. The residue was purified by silica gel column chromatography to give the remote functionalized and protected **19** (3.14 g, 6.45 mmol, 96%) as a light yellow, viscous oil.

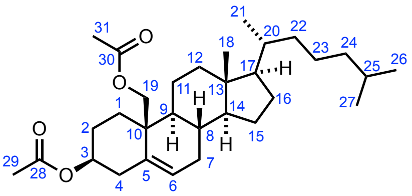


**Formula:** $C_{31}H_{50}O_4$

**Molecular Weight:** 486.74 g/mol

**Yield:** 3.14 g (6.45 mmol, 96%).

**TLC:** $R_f$ = 0.60 (*c*-Hex/EtOAc 3:1), Ce reagent.

**$^1$H NMR** (500 MHz, $CDCl_3$): δ [ppm] = 5.62 (d, *J* = 5.2 Hz, 1H, H-6), 4.62 – 4.60 (m, 1H, H-3), 4.45 (d, *J* = 11.8 Hz, 1H, H-19*β*), 3.98 (d, *J* = 11.8 Hz, 1H, H-19*α*), 2.38 (ddd, *J* = 6.8, 5.6, 1.9 Hz, 1H, H-4*β*), 2.36 – 2.29 (m, 1H, H-4*α*), 2.08 (dt, *J* = 13.6, 3.6 Hz, 1H, H-1*α*), 2.04 (s, 3H, H-29), 2.02 (s, 3H, H-29), 2.01 – 1.97 (m, 1H, H-7), 1.89 – 1.78 (m, 2H, H-16*α*), 1.68 (qd, *J* = 10.8, 5.3 Hz, 1H, H-8), 1.61 – 0.93 (m, 21H), 0.90 (d, *J* = 6.5 Hz, 3H, H-21), 0.86 (dd, *J* = 6.6, 2.2 Hz, 6H, H-26, H-27), 0.68 (s, 3H, H-18).

**$^{13}$C NMR** (125 MHz, $CDCl_3$): δ [ppm] = 170.9 (s, C-30), 170.7 (s, C-28), 134.6 (s, C-5), 127.0 (d, C-6), 73.5 (d, C-3), 64.7 (t, C-19), 57.4 (d, C-14), 56.2 (d, C-17), 50.3 (d, C-9), 42.6 (s, C-13), 40.0 (t, C-12), 39.8 (s, C-10), 39.7 (t, C-24), 38.3 (t, C-4), 36.3 (t, C-22), 35.9 (d, C-20), 33.5 (t, C-1), 33.0 (d, C-8), 31.5 (t, C-7), 28.2 (t, C-16), 28.2 (d, C-25), 28.0 (t, C-2), 24.3 (t, C-23), 24.0 (t, C-15), 23.0 (q, C-27), 22.7 (q, C-26), 21.8 (t, C-11), 21.5 (q, C-29), 21.3 (q, C-31), 18.8 (q, C-21), 12.1 (q, C-18).

**FT-IR (ATR):** $\tilde{\nu}$ [$cm^{-1}$] = 2935 (m), 2868 (m), 1736 (s), 1468 (w), 1444 (w), 1367 (m), 1230 (vs), 1170 (w), 1030 (s), 979 (w), 828 (w), 605 (w).

**HR-MS:** (ESI, 70 eV) = *m/z* calcd. for: $C_{31}H_{50}O_4Na^+$ $[M+Na]^+$ 509.36013 u, found: 509.36010 u.

**$[a]_{\lambda}^{T}$:** (c = 0.52 g/100 mL, $CHCl_3$) = $[a]_{365}^{20}$: –216.28°, $[a]_{436}^{20}$: –131.00°, $[a]_{546}^{20}$: –74.55°, $[a]_{579}^{20}$: –66.09°, $[a]_{D}^{20}$: –62.69°.

### 3.16. Synthesis of 3*β*,19-Diacetoxy-cholestane-5*α*,6*α*-diol (SI-7)

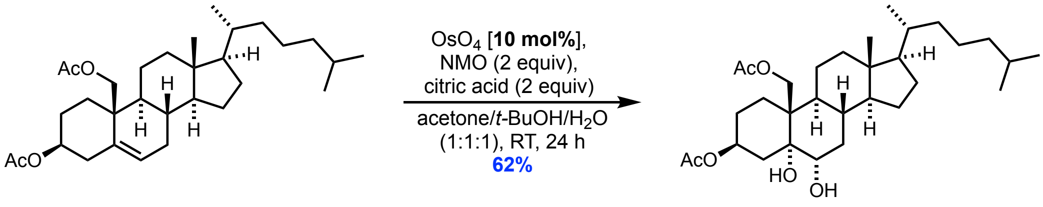


**19** **SI-7**

To a solution of 1.73 g (8.22 mmol, 2.0 equiv) of citric acid in water (13.7 mL) were added a solution

of 2.0 g (4.11 mmol, 1.0 equiv) of alkene **19** in 27.4 mL of acetone/*t*-BuOH (1:1). To the resulting suspension was added under stirring 104 mg (0.41 mmol, 10 mol%, 0.1 equiv) of pure $OsO_4$. To the yellow (slightly cloudy) solution were added after a few minutes 0.96 g (8.22 mmol, 2.0 equiv) of *N*-methyl-morpholin *N*-oxide. The now brown color of the mixture changed to green after some minutes. After 24 h at ambient temperature the (now grey) reaction mixture was quenched by addition of $Na_2SO_3$ (aq. sat.). and stiring was continued for 1 h before the mixture was extracted with EtOAc. The combined organic layers were washed with water, $Na_2SO_3$ (aq. sat.) and brine. After drying over $MgSO_4$, the solvent was removed under reduced pressure and the residue (purple oil) was purified by silica gel column chromatography (*c*-Hex/EtOAc, 10:1 to 2:1) to give **SI-7** (1.33 g, 2.55 mmol, 62%) as a light purple solid.

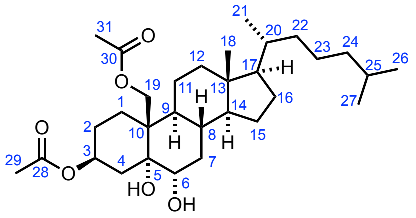


**Formula:** $C_{31}H_{52}O_6$

**Molecular Weight:** 520.75 g/mol

**Yield:** 1.33 g (2.55 mmol, 62%).

**m.p.:** 138 °C – 141 °C.

**TLC:** $R_f$ = 0.14 (*c*-Hex/EtOAc 2:1), Ce reagent.

**$^{1}$H NMR** (500 MHz, $CDCl_3$): δ [ppm] = 5.09 – 5.02 (m, 1H, H-3), 4.28 (dd, *J* = 18.1, 12.2 Hz, 2H, H-19*α*,*β*), 3.51 (dd, *J* = 11.2, 5.0 Hz, 1H, H-6), 3.03 (s, 1H, OH), 2.89 (br s, 1H, OH), 2.33 (dd, *J* = 13.9, 4.3 Hz, 1H, H-4*β*), 2.07 (s, 3H, H-29), 2.00 (s, 3H, H-31), 1.96 – 1.92 (m, 2H, H-2, H-12*β*), 1.85 – 1.78 (m, 2H, H-7, H-16*α*), 1.64 – 0.99 (m, 22H), 0.88 (d, *J* = 6.5 Hz, 3H, H-21), 0.85 (dd, *J* = 6.6, 2.3 Hz, 6H, H-26, H-27), 0.61 (s, 3H, H-18).

**$^{13}$C NMR** (125 MHz, $CDCl_3$): δ [ppm] = 171.4 (s, C-28), 171.1 (s, C-30), 76.0 (s, C-5), 71.3 (d, C-3), 70.6 (d, C-6), 63.2 (t, C-19), 56.2 (d, C-17), 56.2 (d, C-14), 44.1 (d, C-9), 42.8 (s, C-13), 42.8 (s, C-10), 40.3 (t, C-12), 39.6 (t, C-24), 36.3 (t, C-1), 35.9 (d, C-20), 34.7 (t, C-22), 34.7 (t, C-4), 34.2 (d, C-8), 28.3 (t, C-7), 28.1 (d, C-25), 26.9 (t, C-16), 25.4 (t, C-2), 24.2 (t, C-15), 24.0 (t, C-23), 22.9 (q, C-27), 22.7 (q, C-26), 22.1 (t, C-11), 21.6 (q, C-31), 21.3 (q, C-29), 18.7 (q, C-21), 12.2 (q, C-18).

**FT-IR (ATR):** $\tilde{\nu}$ [$cm^{-1}$] = 3408 (br w), 2941 (m), 2905 (m), 2869 (m), 2851 (m), 1739 (m), 1709 (m), 1482 (w), 1463 (w), 1382 (m), 1366 (m), 1233 (vs), 1173 (w), 1156 (w), 1111 (w), 1031 (m), 967 (m), 936 (w), 911 (w), 838 (w), 796 (w), 755 (w), 687 (w), 632 (w), 605 (w), 524 (w).

**HR-MS:** (ESI, 70 eV) = *m/z* calcd. for: $C_{31}H_{52}O_6Na^+$ $[M+Na]^+$ 543.36561 u, found: 543.36517 u.

**$[a]^{T}_{\lambda}$:** (c = 0.51 g/100 mL, $CHCl_3$) = $[a]^{20}_{365}$: –44.97°, $[a]^{20}_{436}$: –45.55°, $[a]^{20}_{546}$: +14.71°, $[a]^{20}_{579}$: +13.14°, $[a]^{20}_{D}$: +12.74°.

### 3.17. Synthesis of 3$\beta$,19-Diacetoxy-5$\alpha$-hydroxy-cholestan-6-one (20)

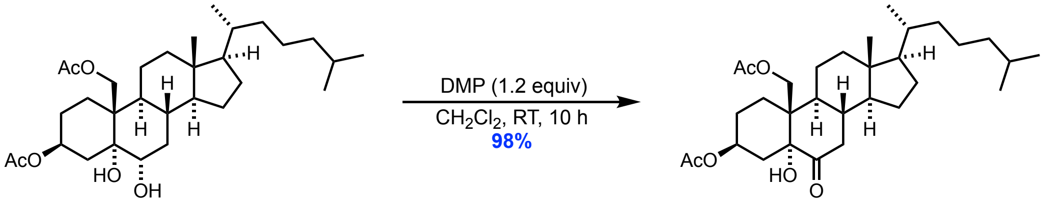


**SI-7** **20**

1.33 g (2.55 mmol, 1.0 equiv) of diol **SI-7** were dissolved in 25 mL $CH_2Cl_2$. 1.30 g (3.06 mmol, 1.2 equiv) of *Dess-Martin* periodinane was added to this solution and the reaction mixture was stirred for 10 h at ambient temperature. After completion of the reaction, the mixture was quenched with a solution of $NaHCO_3$ and $Na_2SO_3$ (1:1, sat. aq.) and extracted with EtOAc. The combined organic layers were washed with brine, dried over $MgSO_4$ and the solvent was removed under reduced pressure. The (purple) residue was purified by silica gel column chromatography (*c*-Hex/EtOAc, 5:1) to give **20** (1.29 g, 2.49 mmol, 98%) as a white foam.

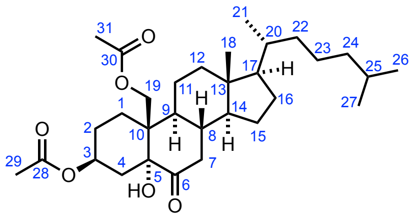

**Formula:** $C_{31}H_{50}O_6$

**Molecular Weight:** 518.74 g/mol

**Yield:** 1.29 g (2.49 mmol, 98%).

**m.p.:** 73 °C – 76 °C.

**TLC:** $R_f$ = 0.30 (*c*-Hex/EtOAc 3:1), Ce reagent.

**$^1$H NMR** (500 MHz, $CDCl_3$): δ [ppm] = 5.12 – 5.05 (m, 1H, H-3), 4.30 (d, $J$ = 12.4 Hz, 1H, H-19$\beta$), 4.09 (d, $J$ = 12.4 Hz, 1H, H-19$\alpha$), 3.21 (s, 1H, OH), 2.64 (dd, $J$ = 14.5, 11.6 Hz, 1H, H-7$\alpha$), 2.19 (dd, $J$ = 14.5, 5.0 Hz, 1H, H-7$\beta$), 2.14 (ddd, $J$ = 14.2, 5.1 Hz, 1.8 Hz, 1H, H-4$\beta$), 2.07 – 1.98 (m, 5H, H-9, H-12$\beta$, H-29), 1.97 (s, 3H, H-31), 1.91 – 1.76 (m, 4H, H-1$\alpha$, H-2$\alpha$, H-8, H-23), 1.71 – 0.97 (m, 19H), 0.91 (d, $J$ = 6.5 Hz, 3H, H-21), 0.86 (dd, $J$ = 6.6, 2.2 Hz, 6H, H-26, H-27), 0.65 (s, 3H, H-18).

**$^{13}$C NMR** (125 MHz, $CDCl_3$): δ [ppm] = 209.8 (s, C-6), 171.2 (s, C-28), 170.5 (s, C-30), 77.6 (s, C-5), 70.2 (d, C-3), 62.4 (t, C-19), 57.0 (d, C-14), 56.2 (d, C-17), 45.3 (s, C-10), 44.0 (d, C-9), 43.2 (s, C-13), 41.6 (t, C-7), 39.9 (t, C-12), 39.6 (t, C-24), 37.3 (d, C-8), 36.3 (t, C-22), 35.9 (d, C-20), 32.3 (t, C-4), 28.2 (t, C-23), 28.1 (d, C-25), 26.4 (t, C-2), 26.0 (t, C-1), 24.1 (t, C-15/16), 24.0 (t, C-15/16), 23.0 (q, C-27), 22.9 (q, C-26), 21.8 (t, C-11), 21.5 (q, C-29), 20.8 (q, C-31), 18.8 (q, C-21), 12.2 (q, C-18).

**FT-IR (ATR):** $\tilde{\nu}$ [cm$^{-1}$] = 3424 (br w), 2946 (m), 2868 (w), 1714 (s), 1467 (w), 1445 (w), 1366 (m), 1230 (s), 1176 (w), 1157 (w), 1157 (w), 1035 (m), 968 (w), 901 (w), 835 (w), 738 (w), 667 (w), 635 (w), 605 (w), 554 (w),

**HR-MS:** (ESI, 70 eV) = *m/z* calcd. for: $C_{31}H_{50}O_6Na^+$ $[M+Na]^+$ 541.34980 u, found: 541.34996 u.

**$[a]_{\lambda}^{T}$:** (c = 0.51 g/100 mL, $CHCl_3$) = $[a]_{365}^{20}$: –258.95°, $[a]_{436}^{20}$: –110.59°, $[a]_{546}^{20}$: –53.20°, $[a]_{579}^{20}$: –45.62°, $[a]_{D}^{20}$: –43.86°.

### 3.18. Synthesis of 3*β*,19-Diacetoxy-5*α*-hydroxy-cholestan-6-one (20)[4]

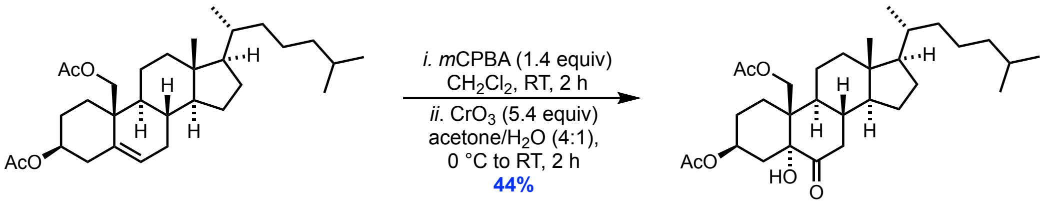


**19** **20**

To a stirred solution of 4.09 g (7.88 mmol, 1.0 equiv) of alkene **19** in $CH_2Cl_2$ (40 mL) were added 2.72 g (11.0 mmol, 1.4 equiv) of *m*CPBA (70%). The mixture was stirred for 1 h at ambient temperature (a white precipitate indiacted comple conversion) and cooled to 0 °C. The precipitate was dissolved by addition of acetone (60 mL) before a solution of 2.84 g (28.4 mmol, 3.6 equiv) of $CrO_3$ in 10 mL of water was added to the clear solution. After 10 min of stirring at 0 °C, the mixture was allowed to warm to ambient temperature and further stirred for 1 h. Then, the above described procedure was repeated with 1.42 g (14.2 mmol, 1.8 equiv) of $CrO_3$ in 5 mL water. After another 2 h TLC indicated a complete conversion. After addition of $NaHCO_3$ (sat. aq.) the aqueous layer was extracted with EtOAc. The combined organic layers were washed with $H_2O$, $NaHCO_3$ (sat. aq.) and brine. The clear yellowish solution was dried over $MgSO_4$ and the solvent was removed under reduced pressure. The residue (brown oil) was purified by silica gel column chromatography (*c*-Hex/EtOAc, 6:1) to give **20** (1.78 g, 3.49 mmol, 44%) as a white foam.

**Formula:** $C_{31}H_{50}O_6$

**Molecular Weight:** 518.14 g/mol

**Yield:** 1.78 g (3.49 mmol, 44%).

➔ The analytical data of **20** matched those of the sample prepared above (see **3.17.**).

### 3.19. Synthesis of 3*β*,19-Diacetoxy-5*α*-hydroxy-7*α*-bromo-cholestan-6-one (SI-8)

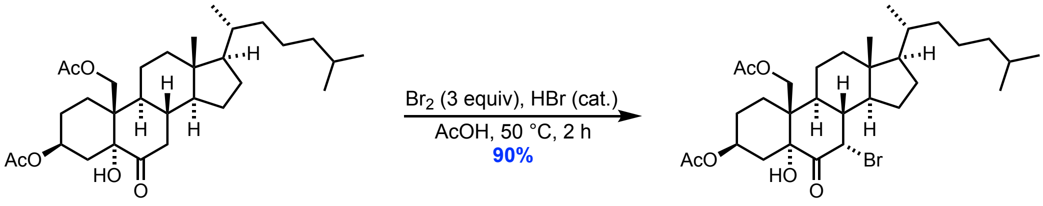


**20** **SI-8**

To a solution of 1.75 g (3.37 mmol, 1.0 equiv) of ketol **20** in acetic acid (35 mL) was added a solution of 0.52 mL (1.62 g, 10.1 mmol, 3.0 equiv) of bromine in acetic acid (3 mL) followed by a few drops of HBr (48% aq.). The mixture was heated to 50 °C for 2 h. The mixture was then cooled to ambient

temperature and quenched with $Na_2SO_3$ (sat. aq.). The aquous layer was extracted with EtOAc and the combined organic layers were washed with water and brine, and dried over $MgSO_4$. The solvent was removed under reduced pressure and the residue was purified by silica gel column chromatography (*c*-Hex/EtOAc, 5:1) to give **SI-8** (1.80 g, 3.01 mmol, 90%) as a beige foam.

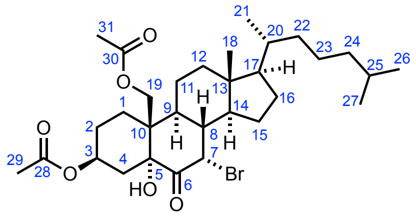

**Formula:** $C_{31}H_{49}O_6Br$

**Molecular Weight:** 597.63 g/mol

**Yield:** 1.80 g (3.01 mmol, 90%).

**m.p.:** 84 °C – 86 °C.

**TLC:** $R_f$ = 0.68 (*c*-Hex/EtOAc 2:1), Ce reagent.

**$^1$H NMR** (600 MHz, $CDCl_3$): δ [ppm] = 5.19 – 5.14 (m, 1H, H-3), 4.43 (d, *J* = 12.7 Hz, 1H, H-19*β*), 4.28 (d, *J* = 4.8 Hz, 1H, H-7), 4.12 (d, *J* = 12.6 Hz, 1H, H-19*α*), 2.84 (s, 1H, OH), 2.41 (ddd, *J* = 13.7, 5.1, 1.6 Hz, 1H, H-4*β*), 2.22 (td, *J* = 12.2, 4.9 Hz, 1H, H-9), 2.08 – 2.04 (m, 1H, H-12*β*), 2.02 (s, 3H, H-29), 1.92 (m, 6H, H-2*α*, H-24, H-31), 1.88 – 1.78 (m, 2H, H-1*α*, H-15*α*), 1.57 – 0.98 (m, 19H), 0.93 (d, *J* = 6.5 Hz, 3H, H-21), 0.87 (dd, *J* = 6.6, 3.0 Hz, 6H, H-26, H-27), 0.69 (s, 3H, H-18).

**$^{13}$C NMR** (150 MHz, $CDCl_3$): δ [ppm] = 201.7 (s, C-6), 170.8 (s, C-28), 170.1 (s, C-30), 78.8 (s, C-5), 69.4 (d, C-3), 62.3 (t, C-19), 55.6 (d, C-17), 53.8 (d, C-7), 53.3 (d, C-14), 45.0 (s, C-10), 42.9 (s, C-13), 39.6 (t, C-24), 39.5 (d, C-8), 38.7 (t, C-12), 37.4 (d, C-9), 36.2 (t, C-22), 35.8 (d, C-20), 33.0 (t, C-4), 28.2 (d, C-25), 28.0 (t, C-16), 26.2 (t, C-2), 26.0 (t, C-1), 23.9 (t, C-23), 23.4 (t, C-15), 23.0 (q, C-27), 22.7 (q, C-26), 21.5 (t, C-11), 21.0 (q, C-29), 20.6 (q, C-31), 18.8 (q, C-21), 12.7 (q, C-18).

**FT-IR (ATR):** $\tilde{\nu}$ [cm$^{-1}$] = 3676 (br w), 3417 (br w), 2945 (m), 2868 (m), 1746 (m), 1713 (s), 1467 (w), 1445 (w), 1366 (m), 1223 (s), 1179 (m), 1154 (m), 1082 (m), 1037 (s), 984 (m), 967 (m), 901 (m), 842 (w), 812 (w), 724 (w), 701 (w), 607 (m), 575 (w), 548 (w).

**HR-MS:** (ESI, 70 eV) = *m/z* calcd. for: $C_{31}H_{49}O_6BrNa^+$ $[M+Na]^+$ 619.26047 u, found: 619.26058 u.

**$[a]_\lambda^T$:** (c = 0.48 g/100 mL, $CHCl_3$) = $[a]_{365}^{20}$: +218.47°, $[a]_{436}^{20}$: –4.44°, $[a]_{546}^{20}$: –17.57°, $[a]_{579}^{20}$: –17.64°, $[a]_{D}^{20}$: –20.42°.

### 3.20. Synthesis of 3$\beta$,19-Diacetoxy-5$\alpha$-hydroxy-cholestan-$\Delta^7$-en-6-one (21)

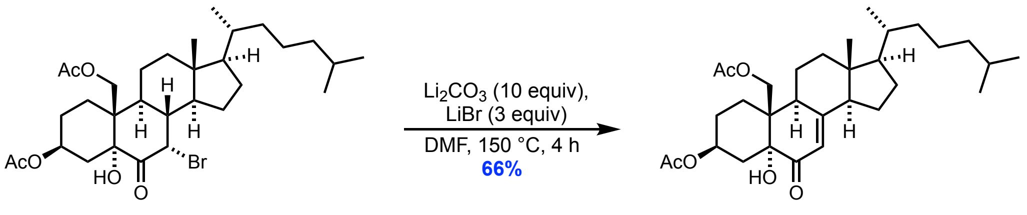


To a solution of 1.36 g (2.27 mmol, 1.0 equiv) of bromide **SI-8** in dimethylformamide (80 mL) were added 1.68 g (22.7 mmol, 10.0 equiv) of $Li_2CO_3$ and 0.59 g (6.81 mmol, 3.0 eq.) of LiBr. Then, the mixture was heated to 150 °C for 4 h and then allowed to cool to ambient temperature. The formed white solid was filtrated off and the filtrate (clear dark yellow solution) was diluted with water. The aqueous layer was extracted with EtOAc and the combined organic layers were washed with water and brine, and dried over $MgSO_4$. After the solvent was removed under reduced pressure, the residue (orange oil) was purified by silica gel column chromatography (*c*-Hex/EtOAc, 5:1) to give **21** (0.71 g, 1.49 mmol, 66%) as a beige foam.

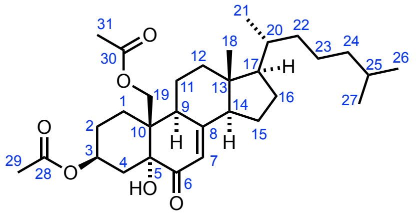

**Formula:** $C_{31}H_{48}O_6$

**Molecular Weight:** 516.72 g/mol

**Yield:** 0.71 g (1.49 mmol, 66%).

**m.p.:** 69 °C – 71 °C.

**TLC:** $R_f$ = 0.32 (*c*-Hex/EtOAc 3:1), Ce reagent.

**$^1$H NMR** (500 MHz, $CDCl_3$): δ [ppm] = 5.63 (s, 1H, H-7), 5.15 – 5.08 (m, 1H, H-3), 4.41 (d, $J$ = 12.7 Hz, 1H, H-19$\beta$), 3.98 (d, $J$ = 12.6 Hz, 1H, H-19$\alpha$), 3.47 (s, 1H, OH), 2.47 (ddd, $J$ = 12.5, 6.2 Hz, 2.2 Hz, 1H, H-9), 2.28 (ddd, $J$ = 14.2, 5.1, 1.3 Hz, 1H, H-4$\beta$), 2.19 – 2.08 (m, 2H, H-24, H-17), 2.02 (s, 3H, H-29), 1.97 – 1.91 (m, 4H, H-1$\alpha$, H-31), 1.86 – 0.98 (m, 23H), 0.94 (d, $J$ = 6.4 Hz, 3H, H-21), 0.87 (dd, $J$ = 6.6, 2.3 Hz, 6H, H-26, H-27), 0.62 (s, 3H, H-18).

**$^{13}$C NMR** (125 MHz, $CDCl_3$): δ [ppm] = 197.3 (s, C-6), 171.0 (s, C-28), 170.4 (s, C-30), 164.6 (s, C-8), 120.2 (d, C-7), 75.6 (s, C-5), 70.4 (d, C-3), 64.0 (t, C-19), 56.5 (d, C-14), 56.1 (d, C-17), 45.1 (s, C-13), 44.0 (d, C-9), 43.8 (s, C-10), 39.5 (t, C-24), 39.3 (t, C-12), 36.1 (d, C-20), 36.1 (t, C-22), 31.9 (t, C-4), 28.1 (d, C-25), 27.8 (t, C-16), 26.8 (t, C-1), 26.8 (t, C-2), 24.1 (t, C-23), 22.9 (q, C-27), 22.6 (q, C-26), 22.6 (t, C-15), 22.4 (t, C-11), 21.4 (q, C-29), 20.8 (q, C-31), 18.9 (q, C-21), 12.4 (q, C-18).

**FT-IR (ATR):** $\tilde{\nu}$ [cm$^{-1}$] = 3507 (br w), 2944 (s), 2934 (s), 2870 (m), 1734 (m), 1713 (s), 1469 (m), 1444 (m), 1381 (m), 1370 (m), 1254 (s), 1245 (vs), 1029 (vs), 977 (m), 961 (m), 911 (w), 886 (w), 824 (w), 807 (w), 742 (w), 670 (w), 610 (w).

**HR-MS:** (ESI, 70 eV) = *m/z* calcd. for: $C_{29}H_{48}O_3Na^+$ [M+Na]$^+$ 539.33431 u, found: 539.33402 u.

**$[a]_{\lambda}^{T}$:** (c = 0.54 g/100 mL, $CHCl_3$) = $[a]_{436}^{20}$: +141.11°, $[a]_{546}^{20}$: +32.78°, $[a]_{579}^{20}$: +24.32°, $[a]_{D}^{20}$: +19.75°.

### 3.21. Synthesis of 3*β*,5*α*-Dihydroxy-8*β*,19-epoxy-cholestan-6-one (4)

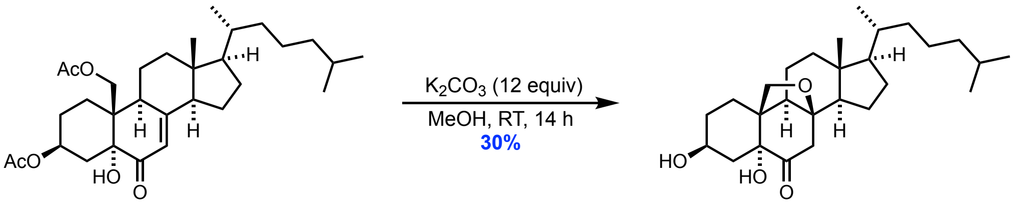


**21** **4**

To a solution of 687 mg (1.33 mmol, 1.0 equiv) of enone **21** in methanol (40 mL) were added 2.20 g (16.0 mmol, 12.0 equiv) of $K_2CO_3$ and the mixture was stirred at ambient temperature over night. After completion of the reaction, the mixture was filtrated to remove excess $K_2CO_3$. The filtrate was then concentrated and diluted with water. After extraction with EtOAc, the combined organic layers were washed with brine, dried over $MgSO_4$ and the solvent was removed under reduced pressure. The residue (beige foam) was purified by silica gel column chromatography (*c*-Hex/EtOAc, 1:1) to give **4** (171 mg, 0.40 mmol, 30%) as a colorles solid.

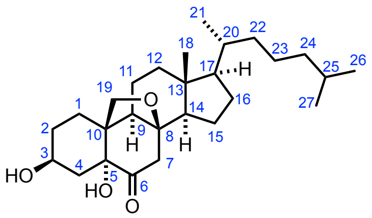


**Formula:** $C_{27}H_{44}O_4$

**Molecular Weight:** 432.65 g/mol

**Yield:** 171 mg (0.40 mmol, 30%).

**m.p.:** 216 °C – 217 °C.

**TLC:** $R_f$ = 0.35 (*c*-Hex/EtOAc 1:1), Ce reagent.

**$^1$H NMR** (500 MHz, $CDCl_3$): δ [ppm] = 3.99 – 3.93 (m, 1H, H-3), 3.78 (d, *J* = 9.6 Hz, 1H, H-19*β*), 3.73 (d, *J* = 9.7 Hz, 1H, H-19*α*), 3.07 (s, 1H, OH), 2.63 (dd, *J* = 23.5, 15.8 Hz, 2H, H-7), 2.47 (dd, *J* = 12.1, 6.5 Hz, 1H, H-9), 2.29 – 2.22 (m, 1H, H-4*β*), 2.11 – 2.06 (m, 1H, H-12*β*), 1.98 – 1.80 (m, 3H, H-1*β*, H-2*α*, H-16*α*), 1.70 – 0.95 (m, 17H), 0.91 (d, *J* = 6.5 Hz, 3H, H-21), 0.86 (dd, *J* = 6.6, 2.3 Hz, 9H, H-26, H-27), 0.81 (s, 3H, H-18).

**$^{13}$C NMR** (125 MHz, $CDCl_3$): δ [ppm] = 208.9 (s, C-6), 84.8 (s, C-8), 78.7 (s, C-5), 72.3 (t, C-19), 67.1 (d, C-3), 56.6 (d, C-17), 53.6 (d, C-14), 50.9 (t, C-7), 50.9 (s, C-10), 44.9 (d, C-9), 42.1 (s, C-13), 39.6 (t, C-24), 38.6 (t, C-12), 38.2 (t, C-4), 36.1 (t, C-22), 35.4 (d, C-20), 32.8 (t, C-2), 28.2 (d, C-25), 27.7 (t, C-16), 24.6 (t, C-1), 23.9 (t, C-23), 23.0 (q, C-27), 22.7 (q, C-26), 20.9 (t, C-11), 20.6 (t, C-15), 18.8 (q, C-21), 12.0 (q, C-18).

**FT-IR (ATR):** $\tilde{\nu}$ [cm$^{-1}$] = 3364 (br w), 2953 (s), 2935 (s), 2869 (s), 1788 (w), 1716 (s), 1467 (m), 1465 (m), 1407 (w), 1375 (m), 1339 (w), 1304 (w), 1249 (m), 1158 (m), 1110 (w), 1073 (m), 1038 (m), 1004 (m), 967 (m), 940 (w), 876 (w), 813 (w),

| | |
|---|---|
| | 728 (w), 624 (w), 580 (w), 530 (w). |
| **HR-MS:** | (ESI, 70 eV) = *m/z* calcd. for: $C_{27}H_{45}O_4^+$ $[M+H]^+$ 433.33124 u, found: 433.33117 u; $C_{27}H_{44}O_4Na^+$ $[M+Na]^+$ 455.31318 u, found: 455.31310 u. |
| **$[a]_{\lambda}^{T}$:** | (c = 0.55 g/100 mL, $CHCl_3$) = $[a]_{365}^{20}$: –234.91°, $[a]_{436}^{20}$: –81.15°, $[a]_{546}^{20}$: –36.36°, $[a]_{579}^{20}$: –30.91°, $[a]_{D}^{20}$: –29.64°. |
| **XRD:** | A single crystalline sample was obtained by recrystallization from $CDCl_3$. |

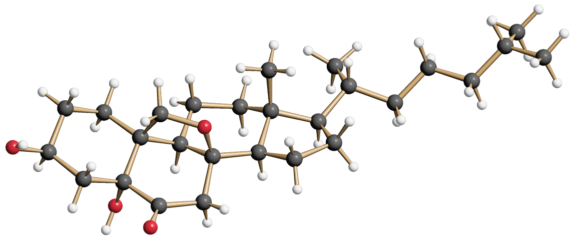

**Figure SI-2.** X-ray crystal structure of 3*β*,5*α*-Dihydroxy-8*β*,19-epoxy-cholestan-6-on (**4**).

### 3.22. Synthesis of 5*α*-Hydroxy-8*β*,19-epoxy-cholestan-6-one 3*β*-sulfonic acid (22)

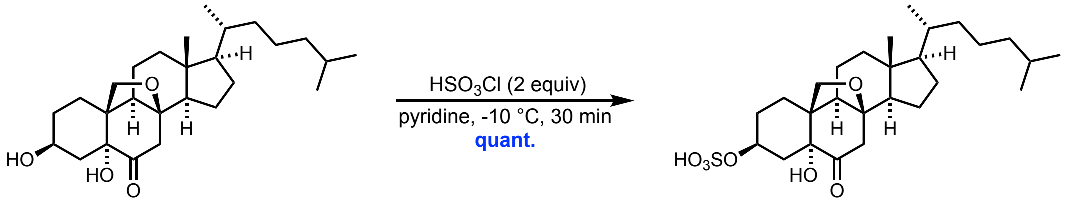


**4** **22**

170 mg (0.39 mmol, 1.0 equiv) of **4** (prepared according to 3.2.1.) were dissolved in pyridine (10 mL) and 0.05 mL (0.79 mmol, 2.0 equiv) of $HSO_3Cl$ were added under an argon atmosphere. The mixture was stirred at -10 °C for 30 min. After completion of the reaction, pyridine was removed under reduced pressure to give a yellowish residue which was purified by silica gel column chromatography ($CH_2Cl_2$/MeOH, 5:1) to afford **22** (199 mg, 0.39 mol, quant.) as a white solid.

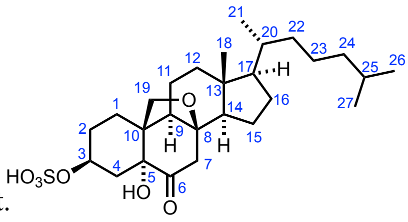


| | |
|---|---|
| **Formula:** | $C_{27}H_{44}O_7S$ |
| **Molecular Weight:** | 512.70 g/mol |
| **Yield:** | 199 mg (0.39 mmol, quant.). |
| **m.p.:** | 203 °C – 206 °C (decomp.). |
| **TLC:** | $R_f$ = 0.28 ($CH_2Cl_2$/MeOH 5:1), Ce reagent. |
| **$^1$H NMR** | (500 MHz, $CD_3OD$): δ [ppm] = 4.61 – 4.54 (m, 1H, H-3), 3.76 (dd, *J* = 9.5, 3.5 Hz, 2H, H-19*α*,*β*), 2.74 (d, *J* = 15.2 Hz, 1H, H-7*α*), 2.56 (dd, *J* = 12.0, 6.7 Hz, 1H, H-9), 2.48 (d, *J* = 15.2 Hz, 1H, H-7*β*), 2.46 (m, 1H, H-20), 2.17 – 2.13 (m, 1H, H-2*α*), 2.11 (dt, *J* = 13.1, 3.7 Hz, 1H, H-4*β*), 2.01 – 1.94 (m, 1H, H-1*α*), 1.93 – 1.85 (m, 1H, H-16*α*), 1.72 – 1.67 (m, 2H, H-1*β*, H-12*β*), 1.66 – 1.26 (m, 12H, H-11*β*, H-15, H-16*β*, H-22, H-23), 1.19 – 1.09 (m, 5H, H-2*β*, H-23, H-24), 1.05 – 1.00 (m, 1H, H-22), 0.94 (d, *J* = 6.5 Hz, 3H, |

| | |
|---|---|
| | H-21), 0.88 (dd, *J* = 6.6, 1.7 Hz, 6H, H-26, H-27), 0.83 (s, 3H, H-18). |
| **$^{13}$C NMR** | (125 MHz, $CD_3OD$): δ [ppm] = 209.7 (s, C-6), 86.3 (s, C-8), 79.3 (s, C-5), 76.1 (d, C-3), 73.1 (t, C-19), 57.7 (d, C-17), 54.5 (d, C-14), 52.3 (s, C-10), 51.7 (t, C-7), 46.1 (d, C-9), 43.1 (s, C-13), 40.7 (t, C-24), 39.7 (t, C-4), 37.1 (t, C-22), 36.6 (d, C-20), 36.2 (t, C-12), 31.4 (t, C-2), 29.1 (d, C-25), 28.6 (t, C-16), 25.5 (t, C-1), 24.9 (t, C-23), 23.2 (q, C-27), 22.9 (q, C-26), 21.8 (t, C-11), 21.4 (t, C-15), 19.2 (q, C-21), 12.4 (q, C-18). |
| **FT-IR (ATR):** | $\tilde{\nu}$ [cm$^{-1}$] = 3404 (br w), 2952 (m), 2931 (m), 2868 (m), 1717 (m), 1639 (w), 1466 (w), 1448 (w), 1378 (w), 1219 (br vs), 1160 (m), 1120 (w), 1073 (m), 1046 (w), 1021 (m), 993 (s), 963 (vs), 900 (w), 858 (m), 811 (m), 747 (w), 658 (w), 628 (w), 591 (s), 544 (w), 528 (w). |
| **$[a]_\lambda^T$:** | (c = 0.48 g/100 mL, MeOH) = $[a]_{365}^{20}$: –255.27°, $[a]_{436}^{20}$: –102.50°, $[a]_{546}^{20}$: –48.33°, $[a]_{579}^{20}$: –41.18°, $[a]_{D}^{20}$: –39.58°. |

### 3.23. Synthesis of eurysterol A (1)

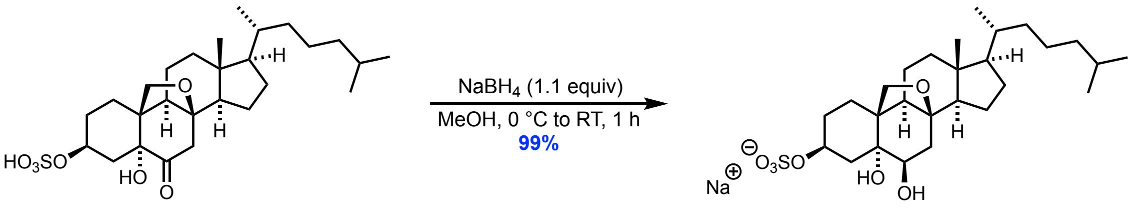


**22** eurysterol A (**1**)

141 mg (0.28 mmol, 1.0 equiv) of **22** were dissolved in methanol (2 mL) and 11 mg (0.30 mmol, 1.1 equiv) of $NaBH_4$ were added at 0 °C. The mixture was stirred at ambient temperature for 1 h. After completion of the reaction (TLC), the solvent was removed and the residue was purified by silica gel chromatography ($CH_2Cl_2$/MeOH, 5:1) to give eurysterol A (150 mg, 0.28 mmol, 99%) as a white solid.

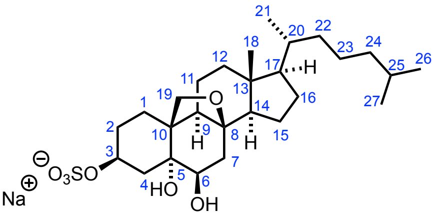


| | |
|---|---|
| **Formula:** | $C_{27}H_{45}O_7SNa$ |
| **Molecular Weight:** | 536.70 g/mol |
| **Yield:** | 150 mg (0.28 mmol, 99%). |
| **m.p.:** | 197 °C – 200 °C (decomp.). |
| **TLC:** | $R_f$ = 0.18 ($CH_2Cl_2$/MeOH 5:1), Ce reagent. |
| **$^1$H NMR** | (500 MHz, $CD_3OD$): δ [ppm] = 4.64 (tt, *J* = 11.4, 4.4. Hz, 1H, H-3), 4.33 (d, *J* = 8.5 Hz, 1H, H-19*β*), 3.64 (d, *J* = 8.5 Hz, 1H, H-19*α*), 3.43 (d, *J* = 4.3 Hz, 1H, H-6), 2.24 (dd, *J* = 13.7, 11.7 Hz, 1H, H-4*β*), 2.17 – 2.11 (m, 1H, H-2*α*), 2.09 – 2.01 (m, 3H, H-1*β*, H-4*α*, H-9, H-12*β*), 1.93 – 1.82 (m, 4H, H-7*α*,*β*, H-1*α*, H-16*α*), 1.70 – 1.64 (m, 1H, H-15*α*), 1.60 – 1.43 (m, 3H, H-1*β*, H-15*β*, H-25), 1.42 – 1.20 (m, 8H, H-11*α*, H-11*β*, H-14, H-16*β*, H-20, H-23), 1.19 – |

0.96 (m, 7H, H-2$\beta$, H-12$\alpha$, H-17, H-22, H-24), 0.93 (d, $J$ = 6.5 Hz, 1H, H-21), 0.88 (dd, $J$ = 6.6, 1.9 Hz, 6H, H-26, H-27), 0.85 (s, 3H, H-18).

**$^{13}$C NMR** (125 MHz, $CD_3OD$): δ [ppm] = 85.6 (s, C-8), 77.4 (s, C-5), 76.7 (d, C-3), 75.6 (d, C-6), 72.2 (t, C-19), 57.8 (d, C-17), 54.8 (d, C-14), 50.3 (s, C-10), 46.5 (d, C-9), 42.8 (s, C-13), 42.6 (t, C-7), 40.7 (t, C-24), 40.0 (t, C-12), 39.9 (t, C-4), 37.1 (t, C-22), 36.7 (d, C-20), 31.7 (t, C-2), 29.1 (d, C-25), 28.7 (t, C-16), 26.3 (t, C-1), 24.9 (t, C-23), 23.2 (q, C-27), 22.9 (q, C-26), 21.6 (t, C-11), 21.5 (t, C-15), 19.2 (q, C-21), 12.5 (q, C-18).

**FT-IR (ATR):** $\tilde{\nu}$ [cm$^{-1}$] = 3449 (br w), 3189 (br w), 2950 (m), 2928 (m), 2868 (m), 2858 (m), 2596 (w), 2383 (w), 1710 (w), 1456 (w), 1378 (w), 1336 (w), 1212 (br s), 1070 (w), 1057 (w), 1025 (w), 1000 (w), 970 (vs), 929 (w), 875 (w), 854 (m), 809 (m), 743 (w), 694 (w), 640 (w), 625 (w), 612 (w), 583 (w), 552 (w), (w).

**HR-MS:** (ESI, 70 eV) = $m/z$ calcd. for: $C_{27}H_{45}O_7S^-$ $[M]^-$ 513.28915 u, found: 513.28981 u.

**$[a]_\lambda^T$:** (c = 0.50 g/100 mL, MeOH) = $[a]_{436}^{20}$: –40.20°, $[a]_{546}^{20}$: –22.20°, $[a]_{579}^{20}$: –19.40°, $[a]_D^{20}$: –19.00°.

**XRD:** A single crystalline sample was obtained by recrystallization from MeOH/ $CH_2Cl_2$.

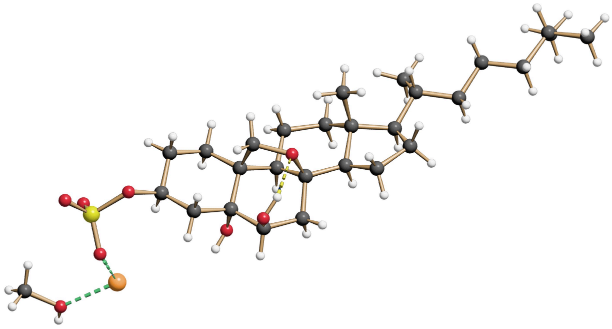

**Figure SI-3.** X-ray crystal structure of eurysterol A (**1**).

### 3.24. Synthesis of 3$\beta$-Acetoxy-19-methoxymethyloxy-cholestan-$\Delta^5$-ene (SI-9)

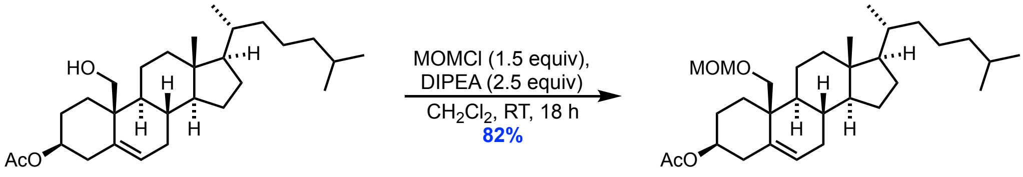


**8** **SI-9**

To a solution of 700 mg (1.57 mmol, 1.0 equiv) of **8** in dry $CH_2Cl_2$ (20 mL) were added 0.67 mL (3.94 mmol, 2.5 equiv) of DIPEA. After stirring for 10 min, 0.19 mL (2.36 mmol, 1.5 equiv) of MOMCl were added and the reaction mixture was stirred for 18 h. After completion (TLC), $NH_4Cl$ (sat. aq.) was added to the mixture and the aqueous layer was extracted with $CH_2Cl_2$. The combined

organic layers were washed with HCl (1 M, aq.), brine and dried over $MgSO_4$. The residue was purified by silica gel column chromatography (*c*-Hex/EtOAc, 5:1) to give **SI-9** (630 mg, 1.30 mmol, 82%) as a white solid.

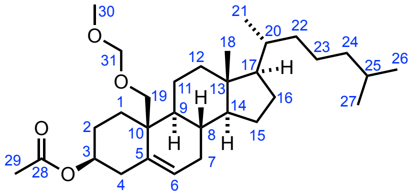

**Formula:** $C_{31}H_{52}O_4$

**Molecular Weight:** 488.75 g/mol

**Yield:** 630 mg (1.29 mmol, 82%).

**m.p.:** 84 °C – 85 °C.

**TLC:** $R_f$ = 0.65 (*c*-Hex/EtOAc 3:1), Ce reagent.

**$^1$H NMR** (500 MHz, $CDCl_3$): δ [ppm] = 5.60 (d, *J* = 5.4 Hz, 1H, H-6), 4.67 – 4.58 (m, 3H, H-3, H-30), 3.73 (d, *J* = 10.3 Hz, 1H, H-19*β*), 3.48 (d, *J* = 10.3 Hz, 1H, H-19*α*), 3.37 (s, 3H, H-31), 2.40 (ddd, *J* = 13.0, 5.2, 2.2 Hz, 1H, H-4*β*), 2.33 (t, *J* = 12.2 Hz, 1H, H-4*α*), 2.11 (dt, *J* = 13.8, 3.7 Hz, 1H, H-1*α*), 2.03 (s, 3H, H-29), 2.02 – 0.96 (m, 25H), 0.91 (d, *J* = 6.5 Hz, 3H, H-21), 0.86 (dd, *J* = 6.6, 2.3 Hz, 6H, H-26, H-27), 0.70 (s, 3H, H-18).

**$^{13}$C NMR** (125 MHz, $CDCl_3$): δ [ppm] = 170.7 (C-28), 135.8 (C-5), 126.2 (C-6), 97.0 (C-30), 73.8 (C-3), 69.1 (C-19), 57.4 (C-14), 56.2 (C-17), 55.6 (C-31), 50.5 (C-9), 42.6 (C-13), 40.5 (C-10), 40.2 (C-12), 39.7 (C-24), 38.5 (C-4), 36.3 (C-22), 36.0 (C-20), 33.4 (C-1), 32.9 (C-8), 31.7 (C-7), 28.4 (C-16), 28.2 (C-2), 28.2 (C-25), 24.3 (C-23), 24.0 (C-15), 23.0 (C-27), 22.7 (C-26), 22.0 (C-11), 21.6 (C-29), 18.8 (C-21), 12.2 (C-18).

**FT-IR (ATR):** $\tilde{\nu}$ [cm$^{-1}$] = 3419 (br w), 2932 (m), 2868 (m), 1733 (m), 1498 (w), 1467 (w), 1444 (w), 1366 (m), 1242 (s), 1144 (m), 1112 (m), 1096 (m), 1046 (s), 1033 (s), 986 (m), 962 (m), 943 (m), 915 (m), 881 (w), 845 (w), 824 (w), 814 (w), 727 (w), 703 (w), 679 (w), 630 (w), 607 (m), 580 (w).

**$[a]^{T}_{\lambda}$:** (c = 0.63 g/100 mL, $CHCl_3$) = $[a]^{20}_{365}$: –136.67°, $[a]^{20}_{436}$: –82.65°, $[a]^{20}_{546}$: –46.56°, $[a]^{20}_{579}$: –41.00°, $[a]^{20}_{D}$: –41.64°.

### 3.25. Synthesis of 3*β*-Acetoxy-5*α*-hydroxy-19-methoxymethyloxy-cholestan-6-one (SI-10)

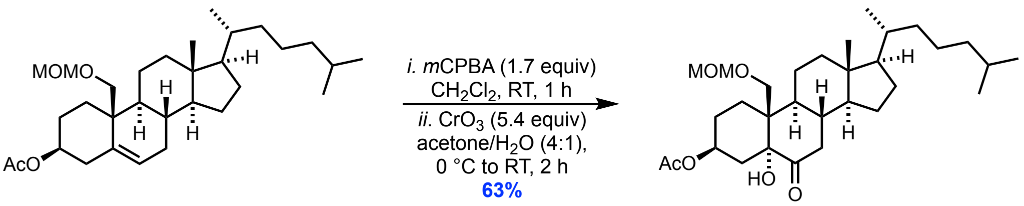


**SI-9** **SI-10**

580 mg (1.19 mmol, 1.0 equiv) of alkene **SI-9** were dissolved in $CH_2Cl_2$ (7 mL) and 490 mg (1.98 mmol, 1.7 equiv) of *m*CPBA (70%) were added to this solution. The mixture was stirred for 1 h at ambient temperature (a white precipitate was observed after the reaction was completed) and then

cooled to 0 °C. By the addition of acetone (17 mL) the white precipitate was dissolved and 429 mg (4.28 mmol, 3.6 equiv) of $CrO_3$ in 1.4 mL water was added to the clear solution. After 10 min of stirring at 0 °C, the reaction mixture was warmed to ambient temperature and stirred for 1 h. Then, the above described procedure was repeated with 214 g (2.14 mmol, 1.8 equiv) of $CrO_3$ in 0.89 mL water. After completion of the reaction (2 h), the reaction mixture was quenched with $NaHCO_3$ (sat. aq.) and the aqueous layer was extracted with EtOAc. The combined organic layers were washed with $H_2O$, $NaHCO_3$ (sat. aq.) and brine. The clear, yellowish solution was dried over $MgSO_4$ and the solvent was removed under reduced pressure. The residue (brown oil) was purified by silica gel column chromatography (*c*-Hex/EtOAc, 8:1) to give **SI-10** (393 g, 0.76 mmol, 63%) as a white foam.

**Formula:** $C_{31}H_{52}O_6$

**Molecular Weight:** 520.75 g/mol

**Yield:** 393 mg (0.76 mmol, 63%).

**m.p.:** 143 °C – 144 °C.

**TLC:** $R_f$ = 0.37 (*c*-Hex/EtOAc 3:1), Ce reagent.

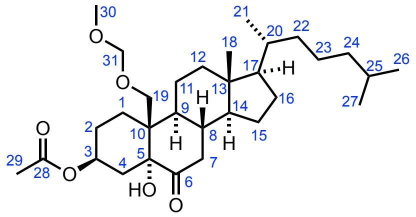

**$^1$H NMR** (500 MHz, $CDCl_3$): δ [ppm] = 5.11 (m, 1H, H-3), 4.50 (s, 2H, H-30), 3.64 (d, *J* = 10.6 Hz, 1H, H-19*β*), 3.57 (d, *J* = 10.6 Hz, 1H, H-19*α*), 3.34 (s, 3H, H-31), 2.75 (s, 1H, OH), 2.57 (dd, *J* = 14.6, 11.6 Hz, 1H, H-7), 2.20 – 2.13 (m, 2H, H-4*β*, H-7), 2.09 – 2.03 (m, 2H, H-8, H-12*β*), 2.02 (s, 3H, H-29), 1.96 – 1.82 (m, 3H, H-2*α*, H-9, H-23), 1.76 – 1.63 (m, 3H, H-1, H-4*α*), 1.59 – 1.43 (m, 5H), 1.39 – 0.96 (m, 12H), 0.91 (d, *J* = 6.5 Hz, 3H, H-21), 0.86 (dd, *J* = 6.6, 2.1 Hz, 6H, H-26, H-27), 0.68 (s, 3H, H-18).

**$^{13}$C NMR** (125 MHz, $CDCl_3$): δ [ppm] = 209.6 (C-6), 171.1 (C-28), 97.1 (C-30), 77.8 (C-5), 70.4 (C-3), 67.1 (C-19), 57.0 (C-14), 56.3 (C-17), 56.1 (C-31), 45.7 (C-10), 44.1 (C-9), 43.2 (C-13), 41.5 (C-7), 40.0 (C-12), 39.6 (C-24), 37.2 (C-8), 36.3 (C-22), 35.9 (C-20), 32.5 (C-4), 28.3 (C-23), 28.1 (C-25), 26.6 (C-2), 26.1 (C-1), 24.0 (C-16), 22.9 (C-27), 22.7 (C-26), 21.9 (C-11), 21.5 (C-29), 18.8 (C-21), 12.3 (C-18).

**FT-IR (ATR):** $\tilde{\nu}$ [cm$^{-1}$] = 3384 (br w), 2939 (m), 2869 (m), 1730 (m), 1713 (s), 1467 (w), 1401 (w), 1382 (m), 1365 (m), 1236 (s), 1150 (m), 1106 (m), 1034 (s), 1012 (s), 967 (m), 940 (m), 920 (m), 904 (m), 871 (w), 834 (w), 734 (w), 664 (w), 941 (w), 609 (w), 553 (w).

**$[a]^{T}_{\lambda}$:** (c = 0.50 g/100 mL, $CHCl_3$) = $[a]^{20}_{365}$: –253.00°, $[a]^{20}_{436}$: –120.47°, $[a]^{20}_{546}$: –62.80°, $[a]^{20}_{579}$: –54.60°, $[a]^{20}_{D}$: –55.13°.

### 3.26. Synthesis of 3*β*-Acetoxy-7*α*-bromo-5*α*,6*α*-dihydroxy-6*β*,19-epoxy-cholestane (23)

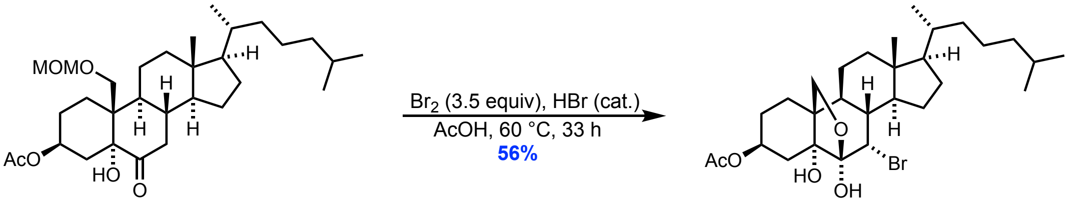


**SI-10** **23**

To a solution of 155 mg (0.29 mmol, 1.0 equiv) of ketol **SI-10** in acetic acid (2.8 mL) were added 0.03 mL (0.16 g, 1.0 mmol, 3.5 equiv) of bromine in acetic acid (0.45 mL) and one drop of HBr (48% aq.). The reaction mixture was heated to 60 °C for 33 h. After completion of the reaction, the mixture was cooled to ambient temperature and quenched with $Na_2SO_3$ (sat. aq.). The aqueous layer was extracted with EtOAc and the combined organic layers were washed with water, brine, and dried over $MgSO_4$. The solvent was removed under reduced pressure and the residue was purified by silica gel column chromatography (*c*-Hex/EtOAc, 5:1) to give **23** (89 mg, 0.16 mmol, 56%) as a white solid.

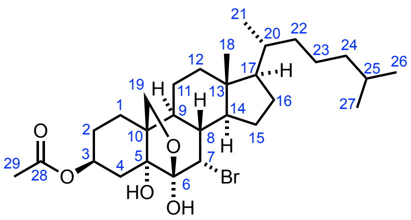

**Formula:** $C_{29}H_{47}BrO_5$

**Molecular Weight:** 555.59 g/mol

**Yield:** 89 mg (0.16 mmol, 56%).

**m.p.:** 124 °C – 125 °C.

**TLC:** $R_f$ = 0.35 (*c*-Hex/EtOAc 2:1), Ce reagent.

**$^1$H NMR** (500 MHz, $CDCl_3$): δ [ppm] = 5.05 – 5.00 (m, 1H, H-3), 4.12 (d, *J* = 4.9 Hz, 1H, H-7), 4.02 (d, *J* = 9.0 Hz, 1H, H-19), 3.73 (d, *J* = 9.0 Hz, 1H, H-19), 3.09 (s, 1H, OH-6), 2.89 (s, 1H, OH-5), 2.19 (ddd, *J* = 12.9, 4.5, 2.2 Hz, 1H, H-4*β*), 2.04 (s, 3H, H-29), 2.05 – 1.99 (m, 1H, H-12*β*), 1.97 – 1.83 (m, 5H, H-2*α*, H-8, H-9, H-15*α*, H-16*α*), 1.74 (t, *J* = 12.4 Hz, 1H, H-4*α*), 1.62 (s, 2H), 1.59 – 0.98 (m, 19H), 0.91 (d, *J* = 6.5 Hz, 3H, H-21), 0.87 (dd, *J* = 6.6, 3.0 Hz, 6H, H-26, H-27), 0.74 (s, 3H, H-18).

**$^{13}$C NMR** (125 MHz, $CDCl_3$): δ [ppm] = 170.7 (C-28), 101.7 (C-6), 79.4 (C5), 69.9 (C-3), 66.8 (C-19), 59.4 (C-7), 55.7 (C-17), 52.9 (C-14), 45.4 (C-10), 43.5 (C-13), 39.6 (C-24), 39.0 (C-12), 38.7 (C-8), 38.6 (C-9), 36.2 (C-22), 35.8 (C-20), 35.0 (C-4), 28.2 (C-25), 28.1 (C-16), 27.2 (C-2), 24.0 (C-1), 24.0 (C-23), 23.3, (C-15), 23.0 (C-27), 22.7 (C-26), 21.6 (C-11), 21.5 (C-29), 18.8 (C-21), 13.1 (C-18).

**FT-IR (ATR):** $\tilde{\nu}$ [cm$^{-1}$] = 3418 (br w), 2933 (m), 2868 (m), 1726 (s), 1714 (s), 1498 (w), 1466 (w), 1382 (m), 1366 (m), 1244 (s), 1154 (m), 1131 (m), 1096 (m), 1042 (s), 984 (m), 965 (s), 942 (s), 906 (m), 846 (m), 814 (w), 727 (w), 703 (m), 680 (m), 666 (m), 629 (m), 609 (m), 583 (m).

**$[a]_\lambda^T$:** (c = 0.51 g/100 mL, $CHCl_3$) = $[a]_{365}^{20}$: –187.24°, $[a]_{436}^{20}$: –86.17°, $[a]_{546}^{20}$:

–50.57°, $[a]^{20}_{579}$: –44.82°, $[a]^{20}_{D}$: –131.49°.

### 3.27. Synthesis of 3*β*-Acetoxy-5*α*-hydroxy-7*β*,19-epoxy-cholestan-6-one (SI-11)

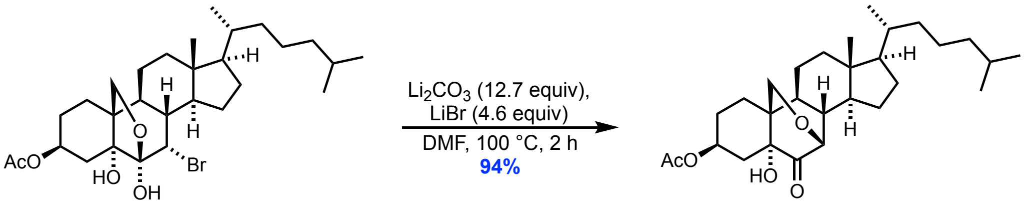


**23** **SI-11**

70 mg (0.13 mmol, 1.0 equiv) of hemiacetal **23** were dissolved in dimethylformamide (6 ml). 122 mg (1.65 mmol, 12.7 equiv) of $Li_2CO_3$ were added to the solution followed by 52 mg (0.60 mmol, 4.6 equiv) of LiBr. The reaction mixture was then heated to 100 °C and stirred for 2 h. After completion of the reaction, the reaction mixture was cooled to ambient temperature and diluted with water. The aqueous layer was extracted with EtOAc, the combined organic layers were washed with water, brine and dried over $MgSO_4$. The solvent was removed under reduced pressure and the crude product was purified by silica gel column chromatography (*c*-Hex/EtOAc, 6:1) to give compound **SI-11** (58 mg, 0.12 mmol, 94%) as a white solid.

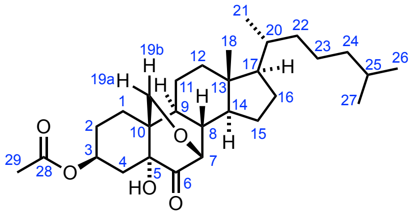

**Formula:** $C_{29}H_{46}O_5$

**Molecular Weight:** 474.68 g/mol

**Yield:** 58 mg (0.12 mmol, 94%).

**m.p.:** 146 °C – 147 °C.

**TLC:** $R_f$ = 0.48 (*c*-Hex/EtOAc 3:1), Ce reagent.

**$^1$H NMR** (500 MHz, $CDCl_3$): δ [ppm] = 5.25 – 5.18 (m, 1H, H-3), 4.14 (dd, *J* = 10.2, 1.7 Hz, 1H, H-19a), 3.91 (d, *J* = 10.2 Hz, 1H, H-19b), 3.82 (d, *J* = 1.7 Hz, 1H, H-7), 2.61 (s, 1H, OH), 2.12 – 2.03 (m, 3H, H-4*β*, H-8, H-12), 2.03 (s, 3H, H-29), 2.00 – 1.94 (m, 2H, H-2*α*, H-4*α*), 1.86 (dt, *J* = 13.0, 9.4, 5.8 Hz, 1H, H-16*α*), 1.73 (td, *J* = 13.8, 4.7 Hz, 1H, H-1*α*), 1.65 – 1.48 (m, 5H), 1.42 – 1.04 (m, 13H), 0.98 (dt, *J* = 10.2, 8.7 Hz, 1H, H-22), 0.89 (d, *J* = 6.5 Hz, 3H, H-21), 0.86 (dd, *J* = 6.6, 2.4 Hz, 6H, H-26, H-27), 0.74 (s, 3H, H-18).

**$^{13}$C NMR** (125 MHz, $CDCl_3$): δ [ppm] = 212.9 (s, C-6), 170.5 (s, C-28), 78.7 (s, C-5), 76.2 (d, C-7), 69.2 (d, C-3), 63.6 (t, C-19), 55.7 (d, C-17), 52.0 (d, C-14), 45.5 (s, C-13), 45.1 (d, C-8), 43.2 (d, C-9), 40.5 (s, C-10), 40.2 (t, C-12), 39.6 (t, C-24), 36.2 (t, C-22), 35.8 (d, C-20), 35.2 (t, C-4), 28.6 (t, C-16), 28.1 (d, C-25), 26.2 (t, C-2), 23.9 (t, C-23), 22.9 (q, C-27), 22.9 (t, C-15), 22.7 (q, C-26), 21.7 (t, C-11), 21.5 (q, C-29), 21.3 (t, C-1), 18.7 (q, C-21), 13.1 (q, C-18).

**FT-IR (ATR):** $\tilde{\nu}$ [cm$^{-1}$] = 3507 (br w), 2944 (s), 2934 (s), 2870 (m), 1734 (m), 1713 (s), 1469 (m), 1444 (m), 1381 (m), 1370 (m), 1254 (s), 1245 (vs), 1029 (vs), 977 (m),

961 (m), 911 (w), 886 (w), 824 (w), 807 (w), 742 (w), 670 (w), 610 (w).

**HR-MS:** (ESI, 70 eV) = *m/z* calcd. for: $C_{29}H_{46}O_5Na^+$ $[M+Na]^+$ 497.32375 u, found: 497.32341 u.

### 3.28. Synthesis of 3*β*,5*α*-Dihydroxy-7*β*,19-epoxy-cholestan-6-one (*iso*-4)

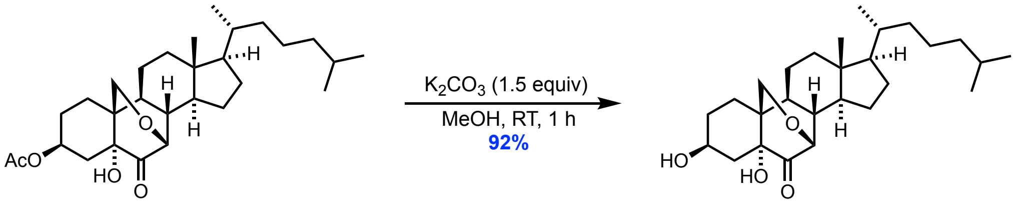


**SI-11** *iso*-**4**

To a solution of 680 mg (1.43 mmol, 1.0 equiv) of **SI-11** in methanol (50 ml) was added 297 mg (2.15 mmol, 1.5 equiv) of $K_2CO_3$ and the mixture was stirred for 1 h at room temperature. After completion of the reaction (TLC), the reaction mixture was diluted with water. The aqueous layer was extracted with EtOAc, the combined organic layers were washed with brine and dried over $MgSO_4$. The solvent was removed under reduced pressure and the crude product was purified by silica gel column chromatography (*c*-Hex/EtOAc, 1:1) to give compound *iso*-**4** (570 mg, 1.32 mmol, 92%) as a white solid.

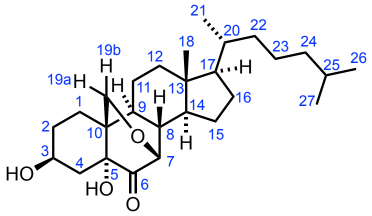

**Formula:** $C_{27}H_{44}O_4$

**Molecular Weight:** 432.65 g/mol

**Yield:** 570 mg (1.32 mmol, 92%).

**m.p.:** 178 °C – 180 °C.

**TLC:** $R_f$ = 0.19 (*c*-Hex/EtOAc 1:1), Ce reagent.

**$^1$H NMR** (600 MHz, $CDCl_3$): δ [ppm] = 4.19 – 4.13 (m, 2H, H-3, H-19a), 3.88 (d, *J* = 10.2, 1H, H-19b), 3.81 (s, 1H, H-7), 2.71 (s, 1H, OH-5), 2.11 – 2.00 (m, 3H, H-4*β*, H-8, H-12), 1.96 – 1.82 (m, 3H, H-2*α*, H-4*α*, H-16*α*), 1.75 (s, 1H, OH-3), 1.65 – 0.94 (m, 20H), 0.88 (d, *J* = 6.5 Hz, 3H, H-21), 0.85 (dd, *J* = 6.6, 2.9 Hz, 6H, H-26, H-27), 0.73 (s, 3H, H-18).

**$^{13}$C NMR** (150 MHz, $CDCl_3$): δ [ppm] = 213.6 (s, C-6), 79.2 (s, C-5), 76.2 (d, C-7), 66.2 (d, C-3), 63.7 (t, C-19), 55.7 (d, C-17), 52.1 (d, C-14), 45.5 (s, C-13), 45.2 (d, C-8), 43.3 (d, C-9), 40.6 (s, C-10), 40.3 (t, C-12), 39.6 (t, C-24), 38.8 (t, C-2), 36.2 (t, C-22), 35.8 (d, C-20), 30.2 (t, C-4), 28.6 (t, C-16), 28.1 (d, C-25), 23.9 (t, C-23), 22.9 (t, C-15), 22.9 (q, C-27), 22.7 (q, C-26), 21.8 (t, C-11), 21.5 (t, C-1), 18.7 (q, C-21), 13.1 (q, C-18).

**FT-IR (ATR):** $\tilde{\nu}$ [cm$^{-1}$] = 3463 (br w), 2948 (s), 2868 (m), 1733 (vs), 1497 (w), 1466 (m), 1414 (w), 1382 (m), 1365 (w), 1340 (w), 1296 (w), 1248 (m), 1225 (w), 1153 (m), 1051 (vs), 968 (m), 958 (m), 887 (w), 818 (w), 779 (w), 756 (m), 713 (w),

637 (w), 558 (m), 539 (w).

**$[a]_{\lambda}^{T}$:** (c = 0.99 g/100 mL, $CHCl_3$) = $[a]_{365}^{20}$: +261.18°, $[a]_{436}^{20}$: +125.08°, $[a]_{546}^{20}$: +66.90°, $[a]_{579}^{20}$: +58.01°, $[a]_{D}^{20}$: +55.22°.

# 4. [1]H and [13]C NMR spectra

## 4.1 NMR spectra of 3*β*-Acetoxy-5*α*-bromo-cholestan-6*β*-ol (10)

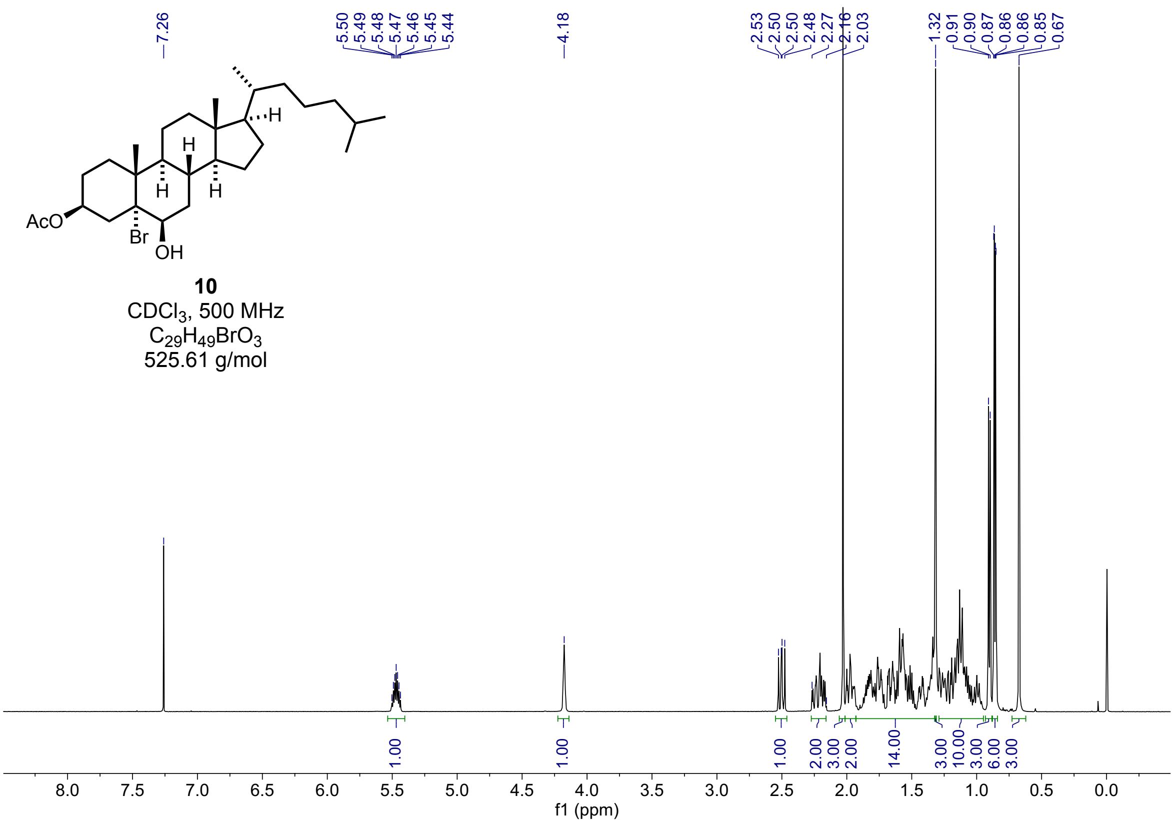


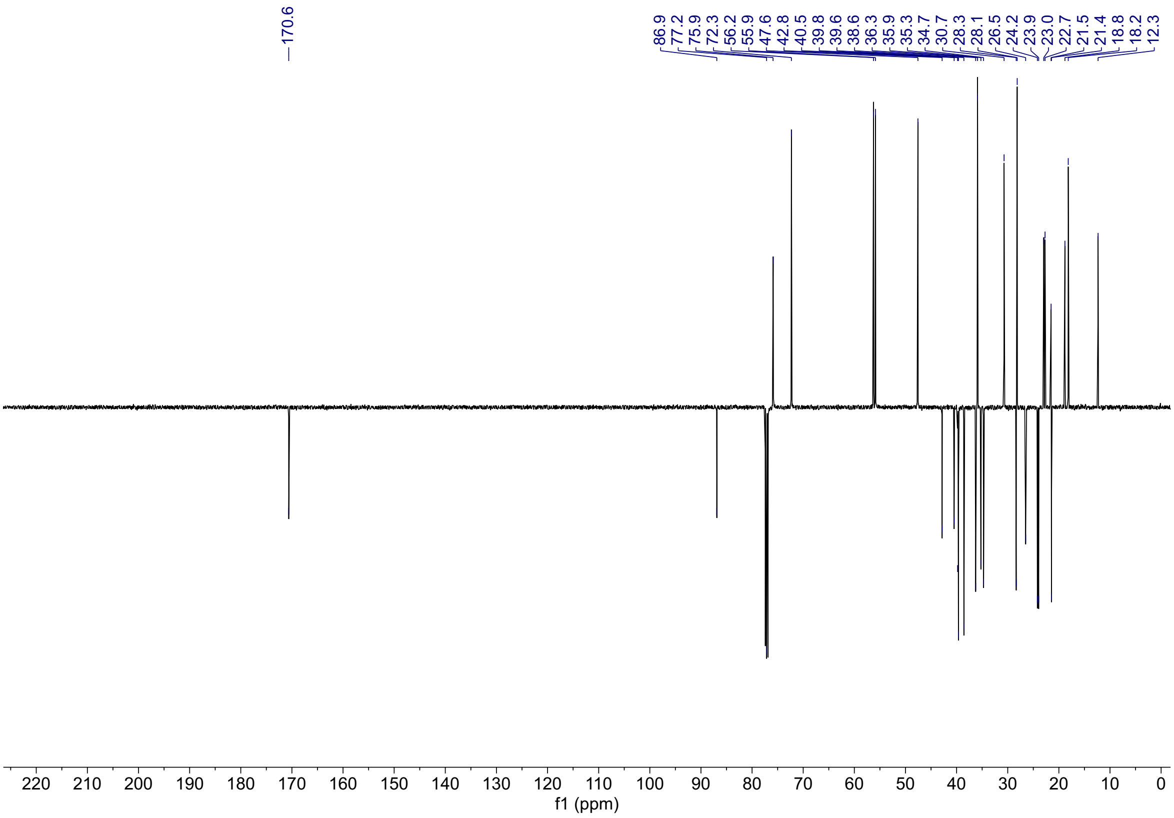


**Figure SI-4.** [1]H and [13]C NMR spectra of 3*β*-Acetoxy-5*α*-bromocholestan-6*β*-ol (**10**).

## 4.2 NMR spectra of 3*β*-Acetoxy-5*α*-bromo-6*β*,19*β*-epoxycholestane (11)

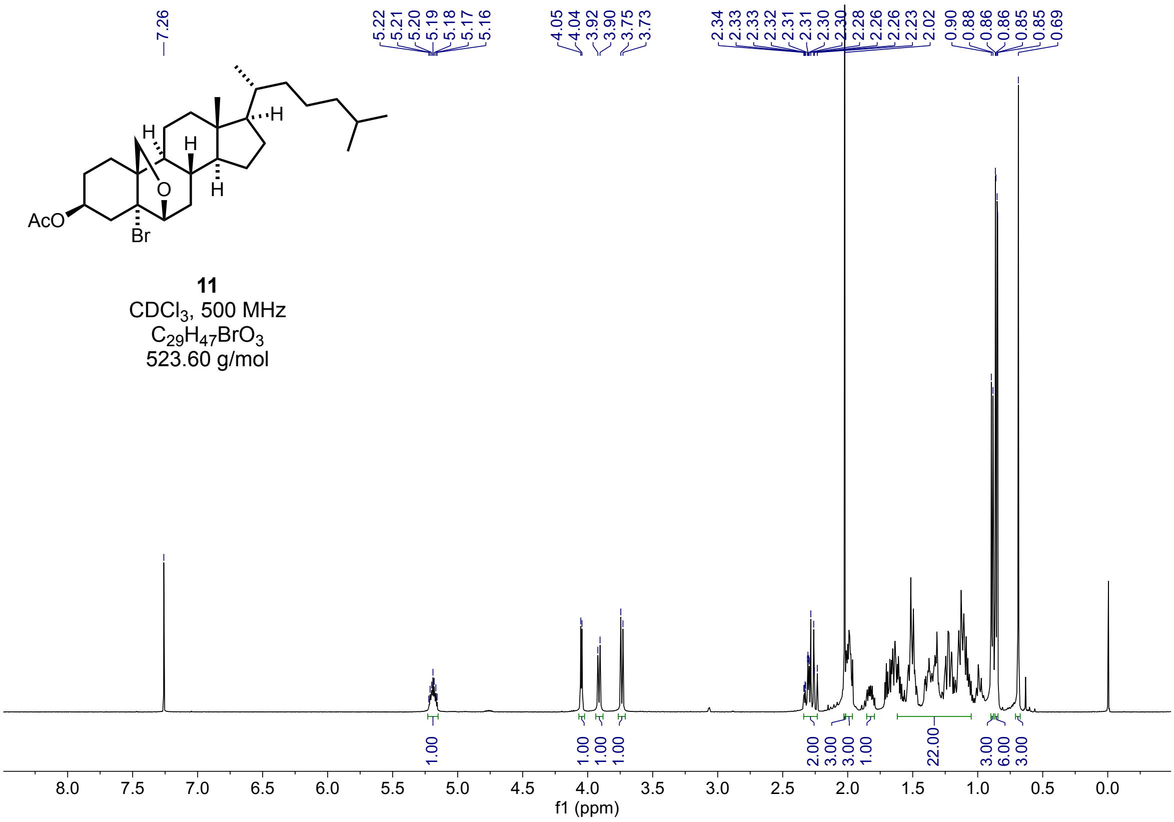


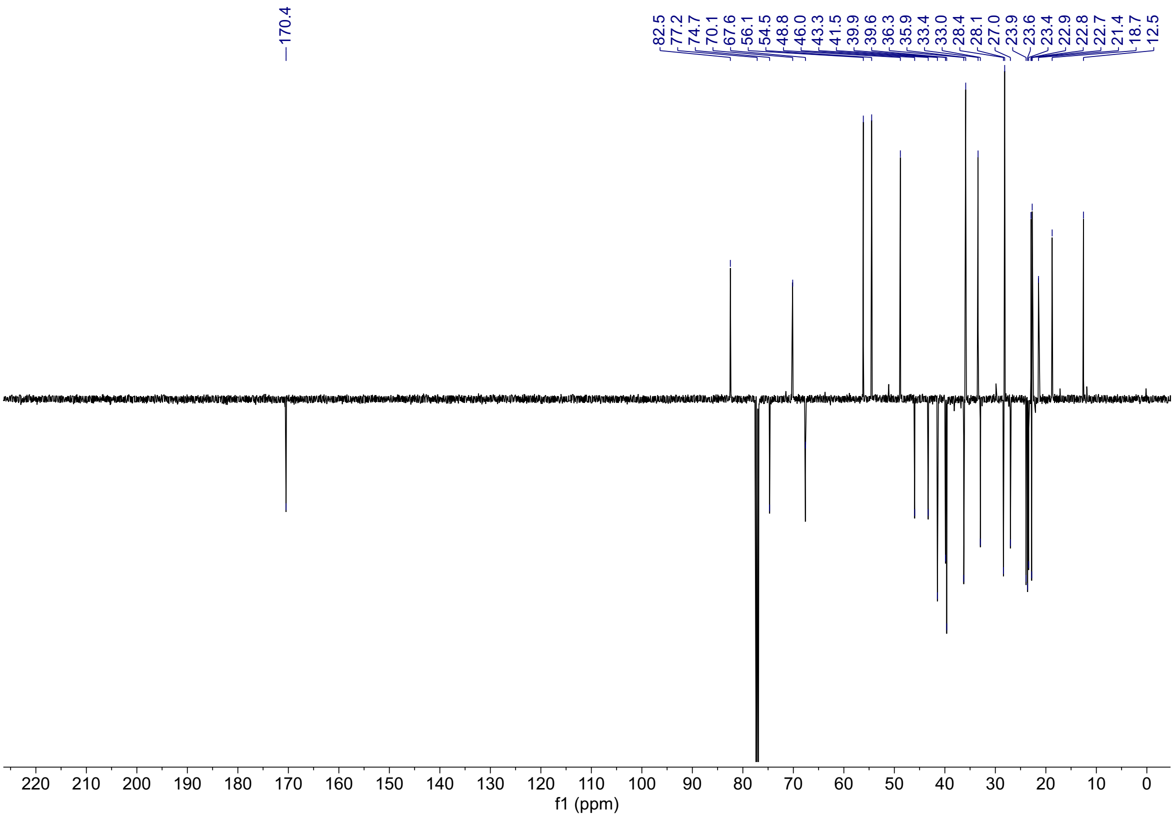


**Figure SI-5.** $^{1}$H and $^{13}$C NMR spectra of 3*β*-Acetoxy-5*α*-bromo-6*β*,19*β*-epoxycholestane (**11**).

## 4.3 NMR spectra of 3*β*-Acetoxy-cholest-$\Delta^5$-en-19*β*-ol (8)

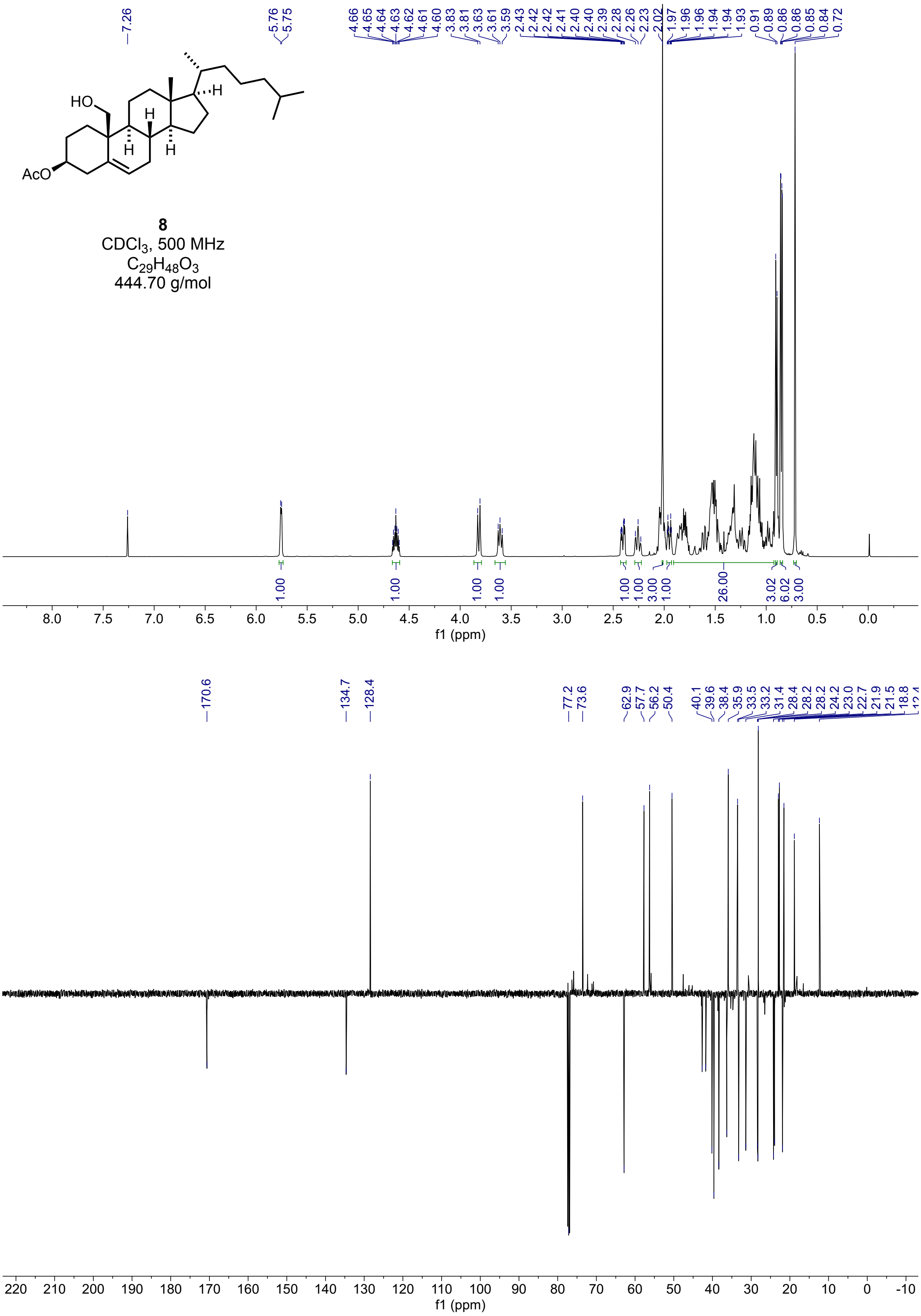


**Figure SI-6.** $^{1}$H and $^{13}$C NMR spectra of 3*β*-Acetoxy-cholest-$\Delta^5$-en-19*β*-ol (**8**).

## 4.4 NMR spectra of 3*β*-Acetoxy-19-*tert*-butyldimethylsilyloxy-cholest-Δ[5]-ene (12)

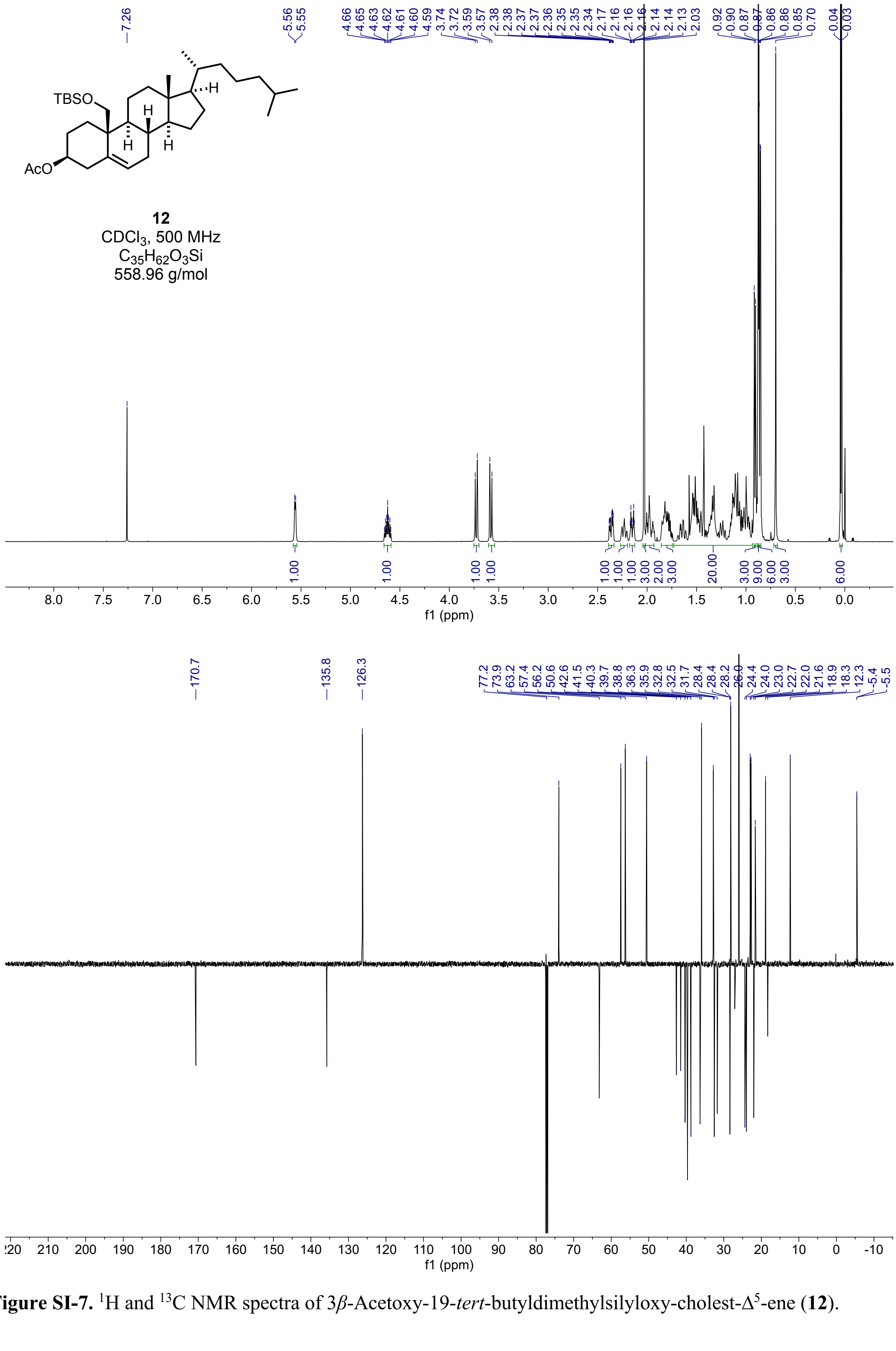


**Figure SI-7.** [1]H and [13]C NMR spectra of 3*β*-Acetoxy-19-*tert*-butyldimethylsilyloxy-cholest-Δ[5]-ene (**12**).

## 4.5 NMR spectra of 3*β*-Acetoxy-19-*tert*-butyldimethylsilyloxy-cholest-$\Delta^5$-en-7-one (SI-1)

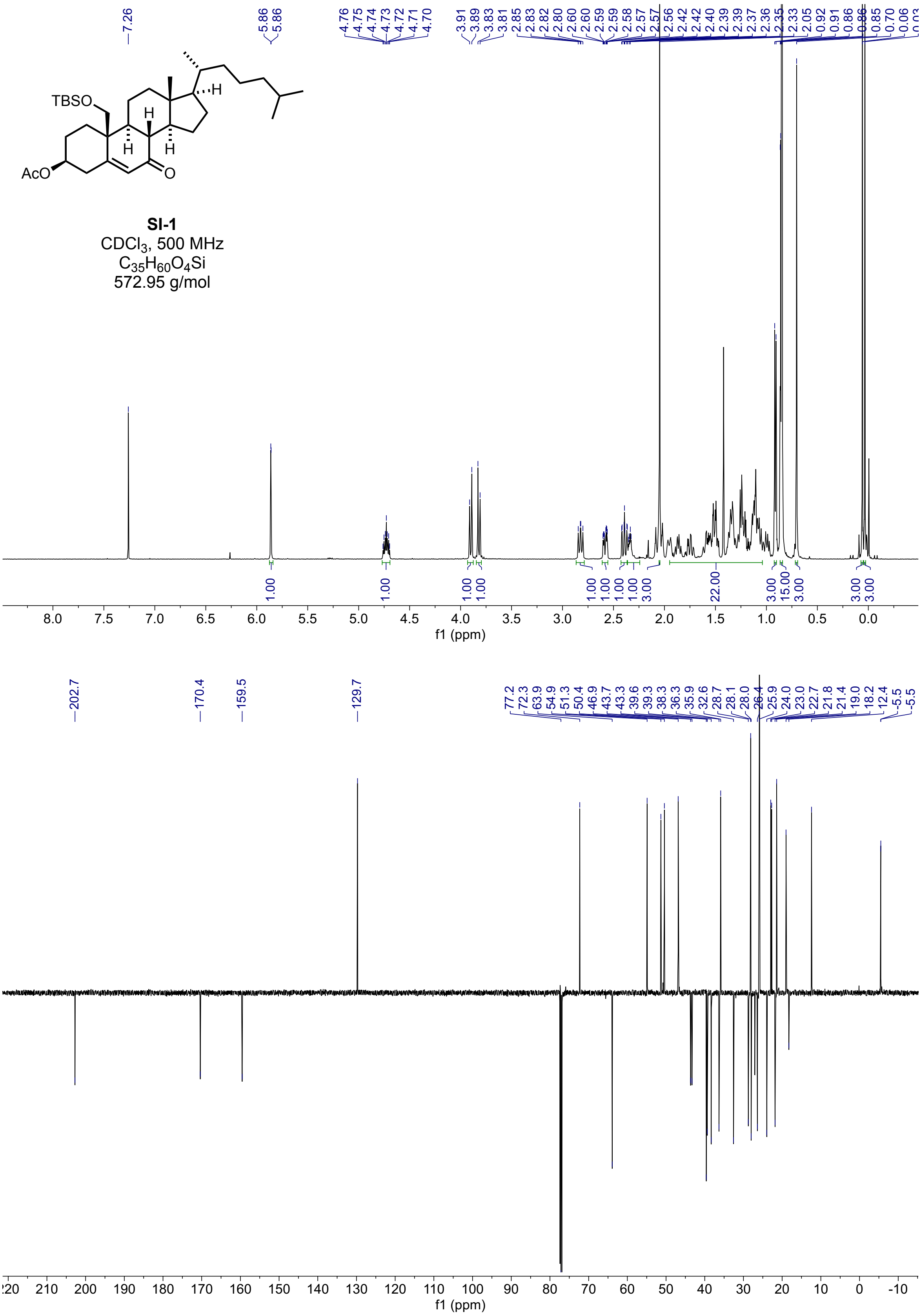


**Figure SI-8.** $^1$H and $^{13}$C NMR spectra of 3*β*-Acetoxy-19-*tert*-butyldimethylsilyloxy-cholest-$\Delta^5$-en-7-one (**SI-1**).

## 4.6 NMR spectra of 19-*tert*-Butyldimethylsilyloxy-cholest-$\Delta^5$-en-3$\beta$-ol (SI-2)

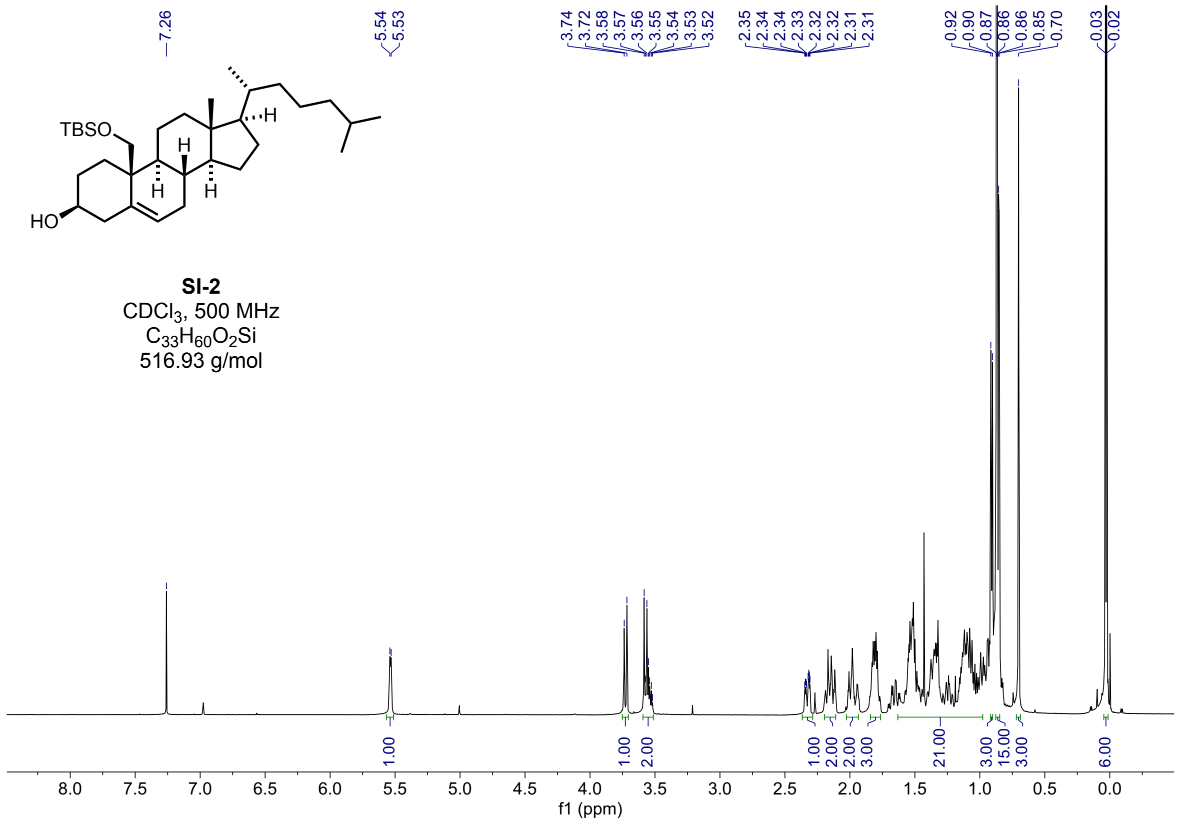


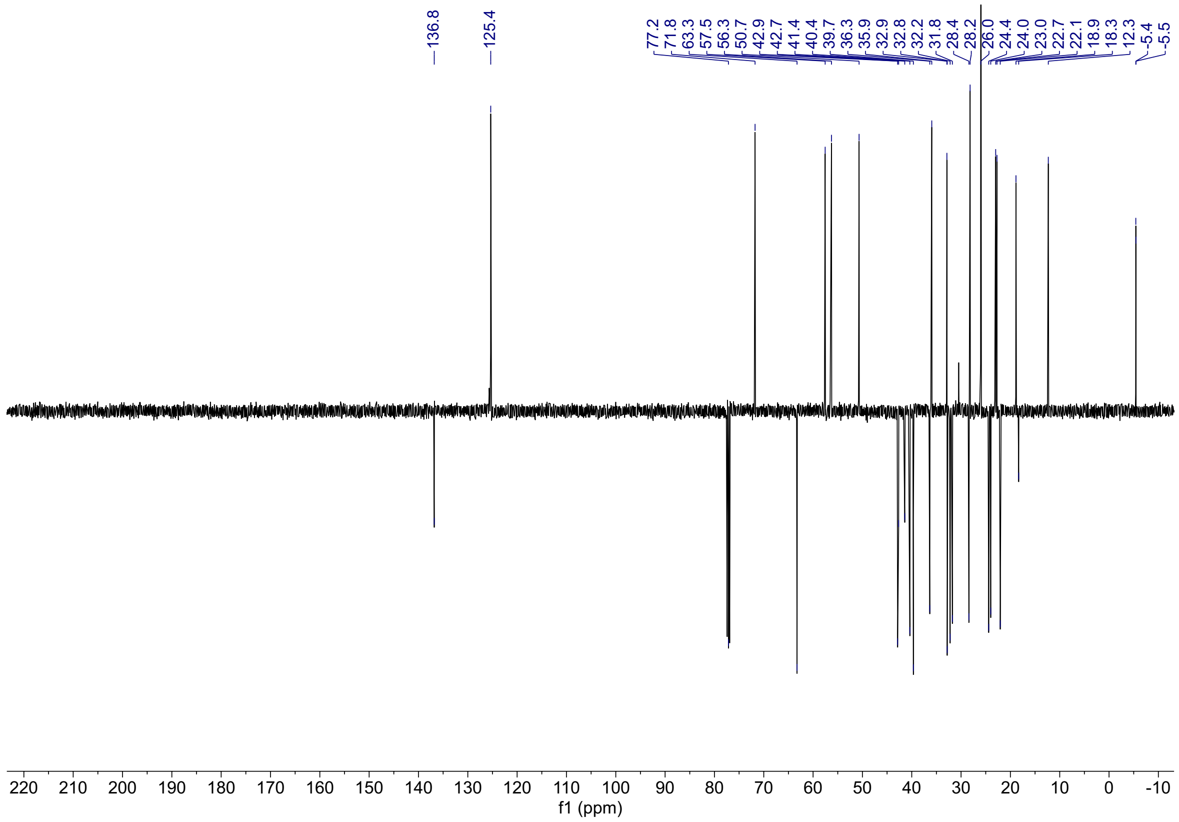


**Figure SI-9.** [1]H and [13]C NMR spectra of 19-*tert*-Butyldimethylsilyloxy-cholest-$\Delta^5$-en-3$\beta$-ol (**SI-2**).

## 4.7 NMR spectra of 3$\beta$-Methoxymethyloxy-19-*tert*-butyldimethylsilyloxy-cholest-$\Delta^5$-ene (13)

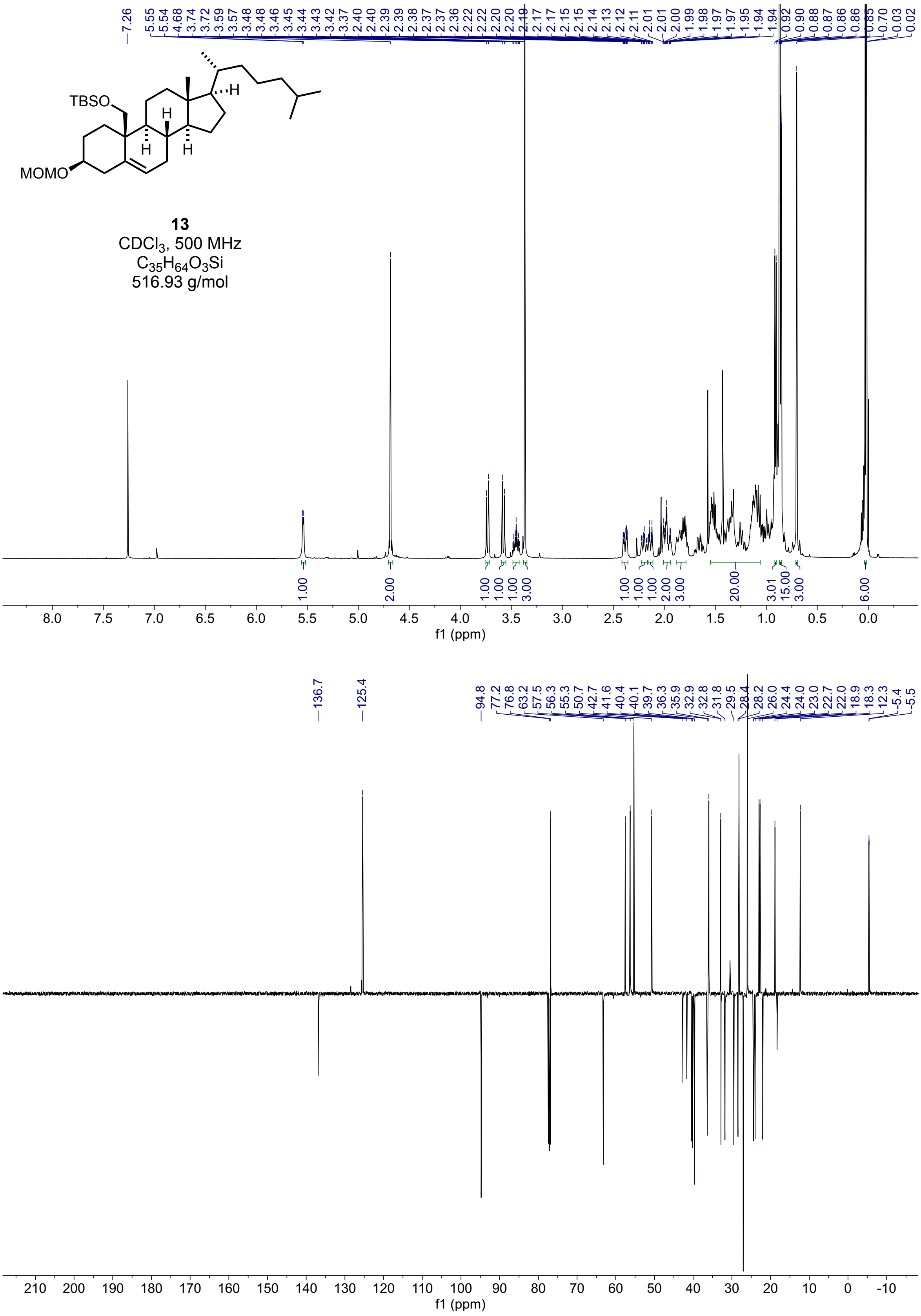


**Figure SI-10.** $^1$H and $^{13}$C NMR spectra of 3$\beta$-Methoxymethyloxy-19-*tert*-butyldimethylsilyloxy-cholest-$\Delta^5$-ene (**13**).

## 4.8 NMR spectra of 3*β*-Methoxymethyloxy-19-*tert*-butyldimethylsilyloxy-cholest-Δ[5]-en-7-one (14)

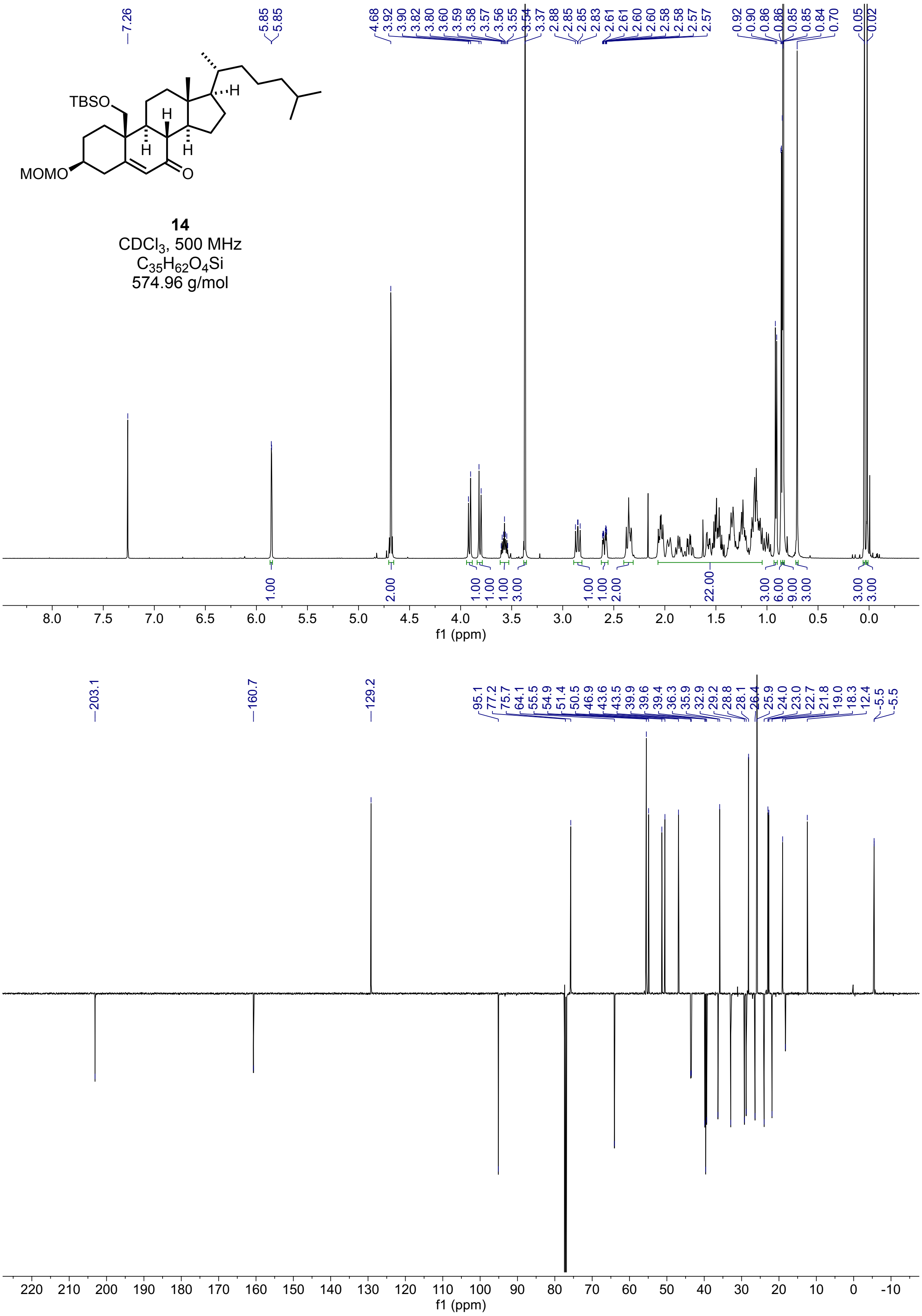


**Figure SI-11.** $^{1}$H and $^{13}$C NMR spectra of 3*β*-Methoxymethyloxy-19-*tert*-butyldimethylsilyloxy-cholest-Δ$^{5}$-en-7-one (**14**).

## 4.9 NMR spectra of 3*β*-Acetoxy-5*α*-bromo-cholestan-6*β*-ol (10)

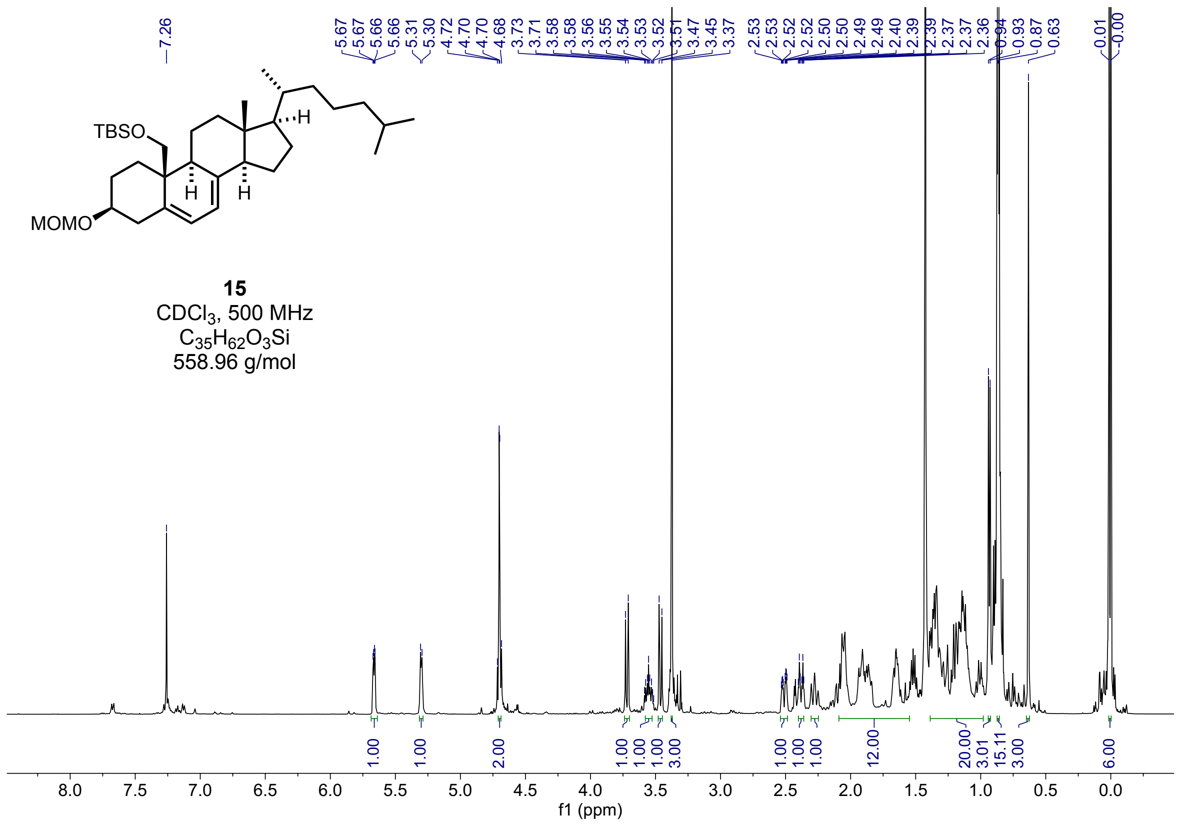


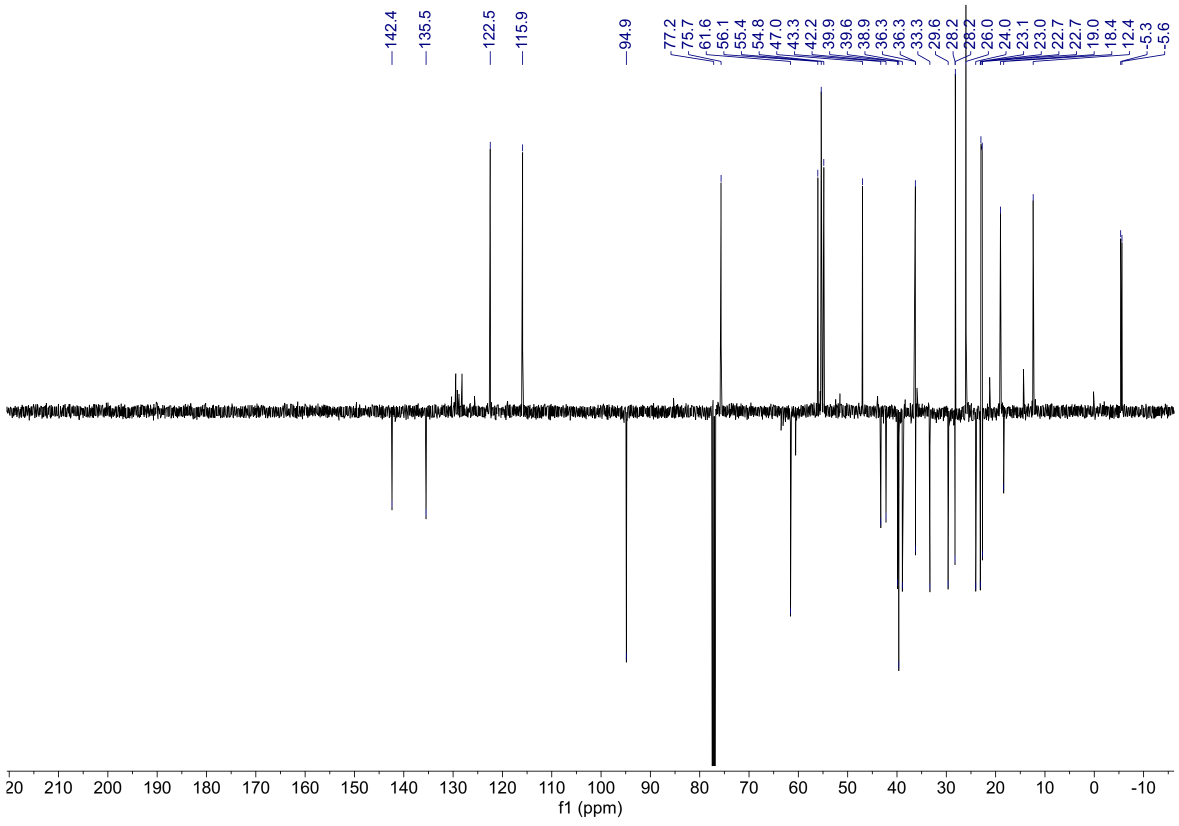


**Figure SI-12.** [1]H and [13]C NMR spectra of 3*β*-Methoxymethyloxy-19-*tert*-butyldimethylsilyloxy-cholest-$\Delta^{5,7}$-diene (**15**).

## 4.10 NMR spectra of 3$\beta$-Methoxymethyloxy-5$\alpha$-hydroxy-19-*tert*-butyldimethylsilyloxy-cholest-$\Delta^7$-en-6-one (SI-4)

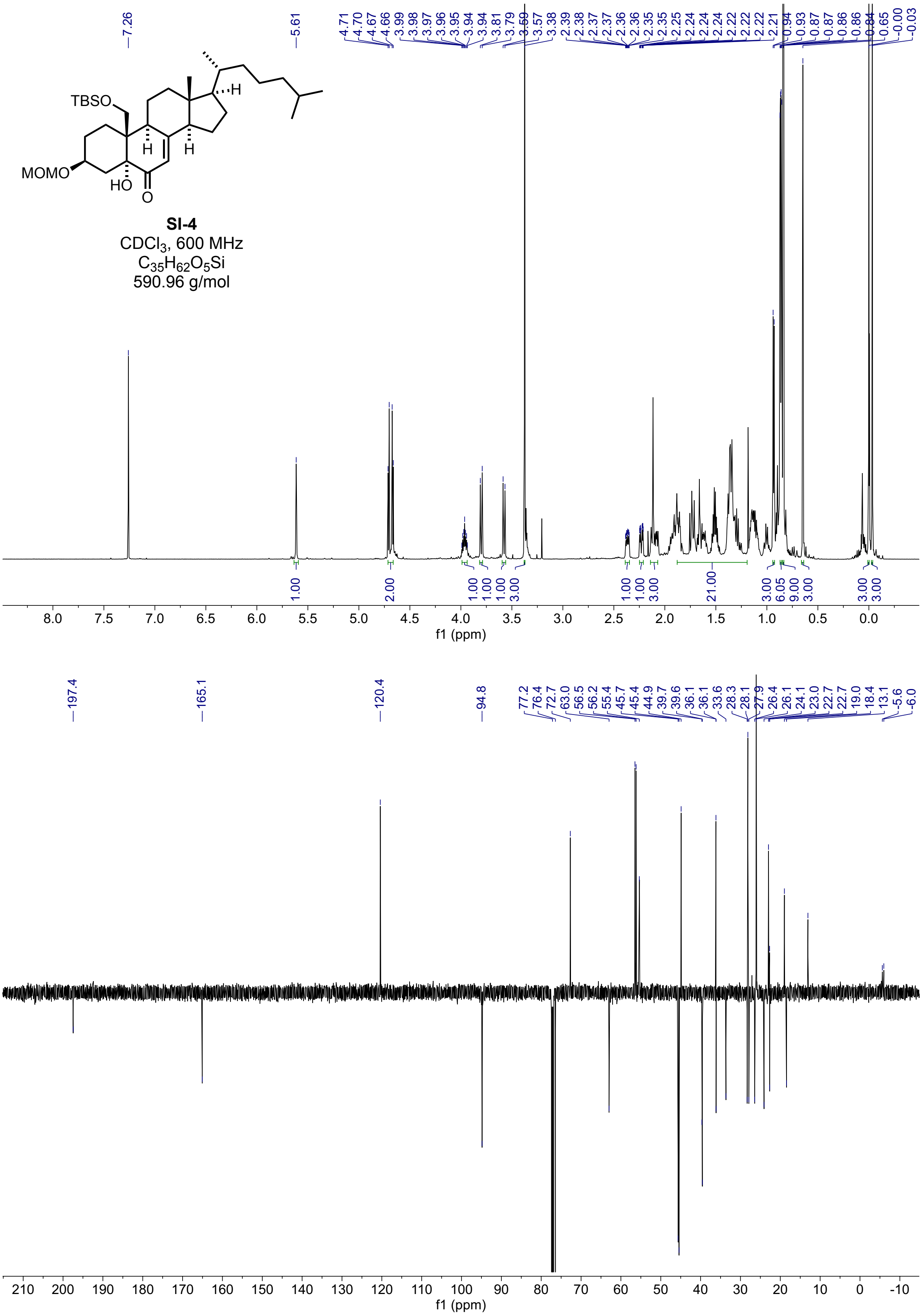


**Figure SI-13.** $^1$H and $^{13}$C NMR spectra of 3$\beta$-Methoxymethyloxy-5$\alpha$-hydroxy-19-*tert*-butyldimethylsilyloxy-cholest-$\Delta^7$-en-6-one (**SI-4**).

## 4.11 NMR spectra of 3*β*-Methoxy-19-hydroxy-cholest-$\Delta^{5,7}$-diene (SI-5)

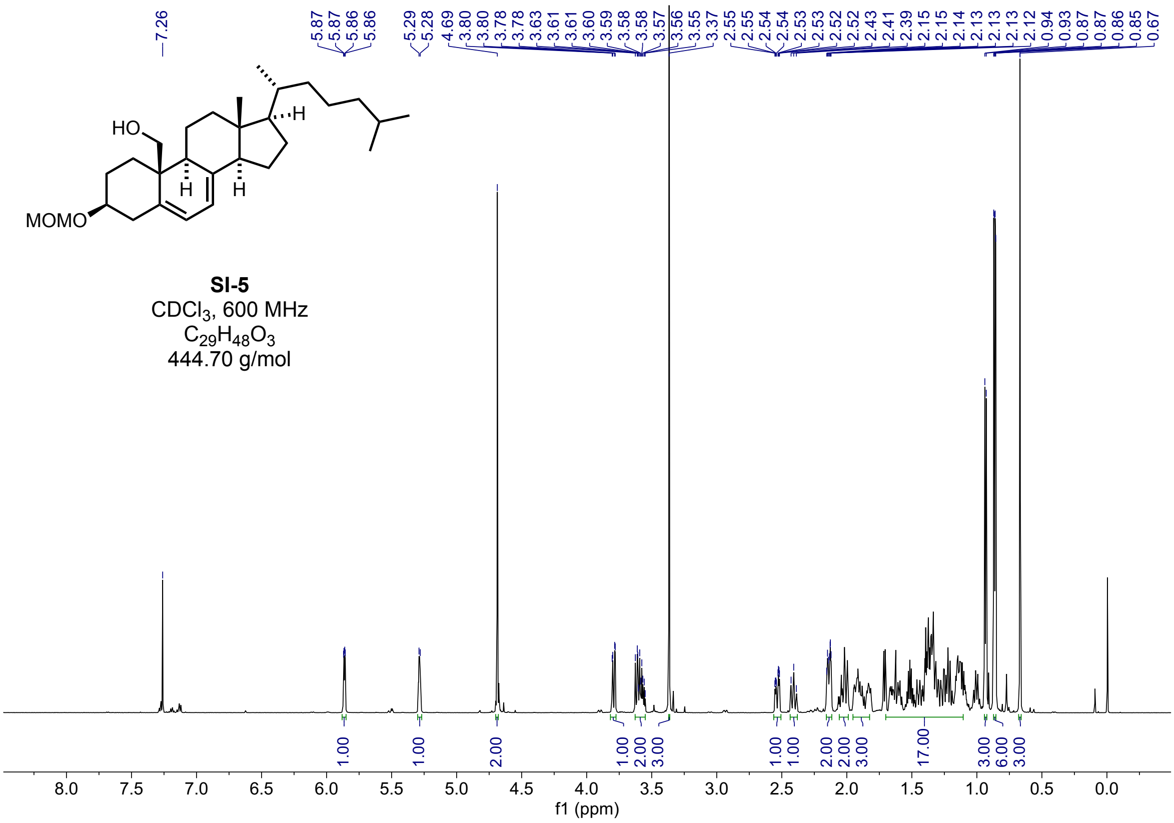


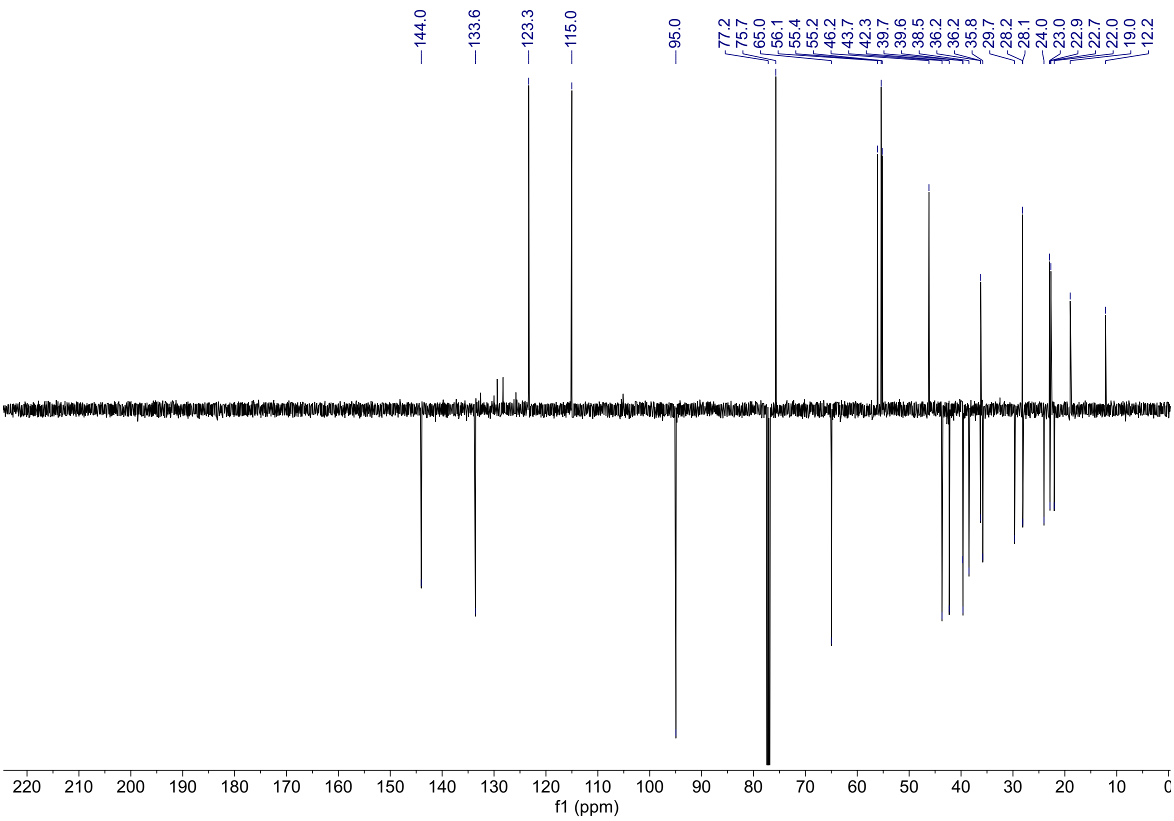


**Figure SI-14.** $^{1}$H and $^{13}$C NMR spectra of 3*β*-Methoxy-19-hydroxy-cholest-$\Delta^{5,7}$-diene (**SI-5**).

## 4.12 NMR spectra of 3*β*-Methoxymethyloxy-19-acetoxy-cholest-$\Delta^{5,7}$-diene (16)

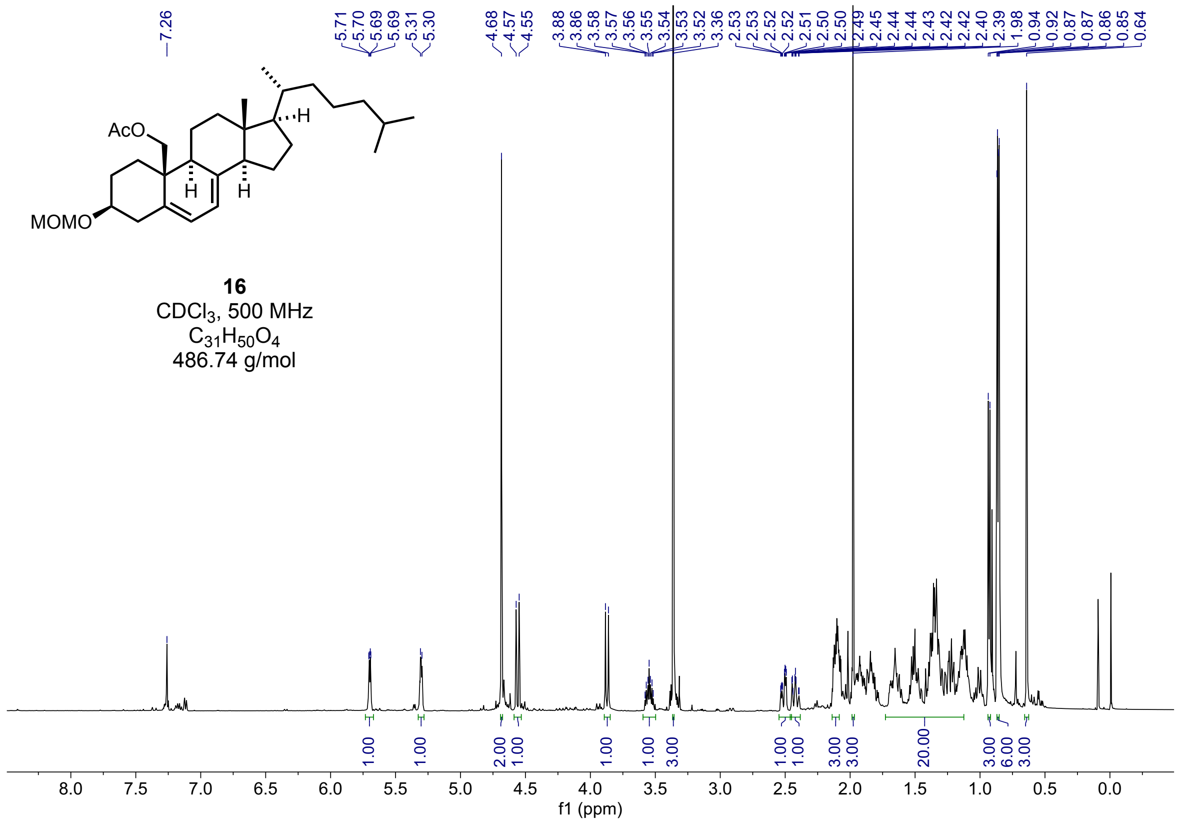


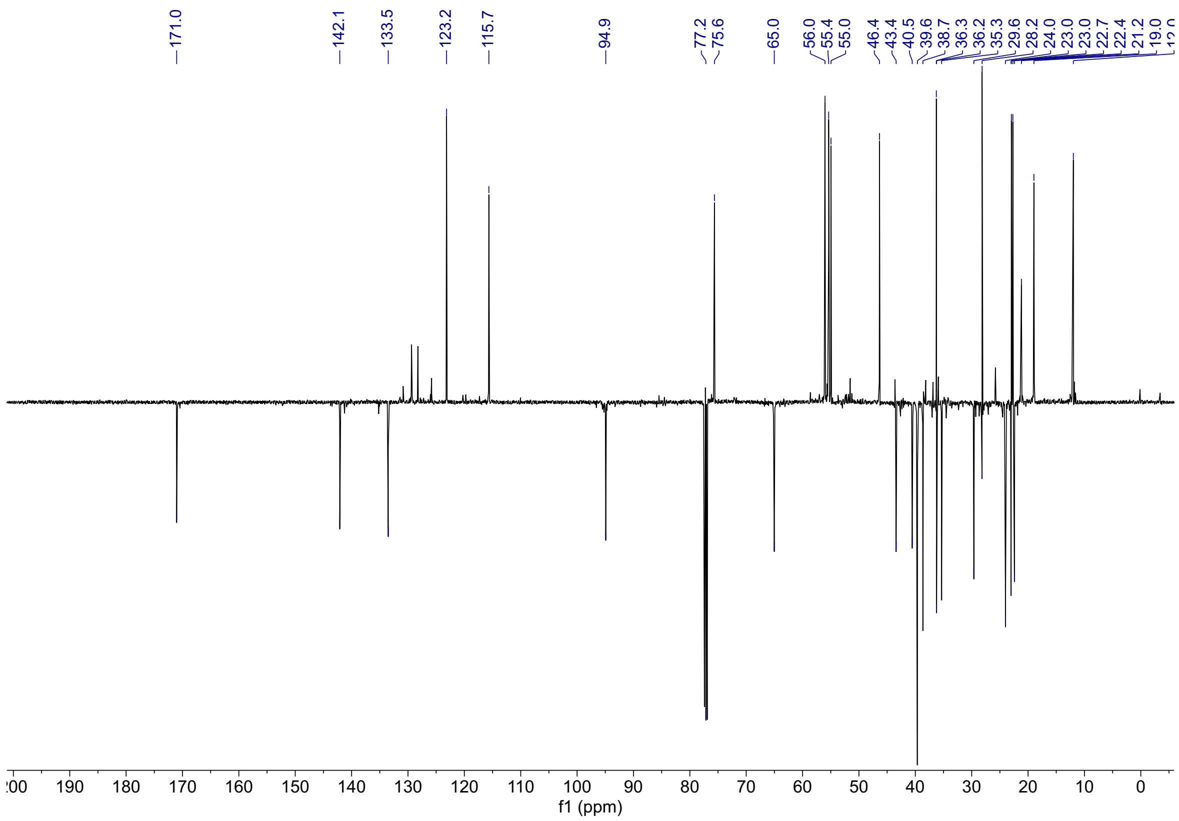


**Figure SI-15.** $^{1}$H and $^{13}$C NMR spectra of 3*β*-Methoxymethyloxy-19-acetoxy-cholest-$\Delta^{5,7}$-diene (**16**).

### 4.13 NMR spectra of 3*β*-Methoxymethyloxy-5*α*-hydroxy-19-acetoxy-cholest-$\Delta^7$-en-6-one (17)

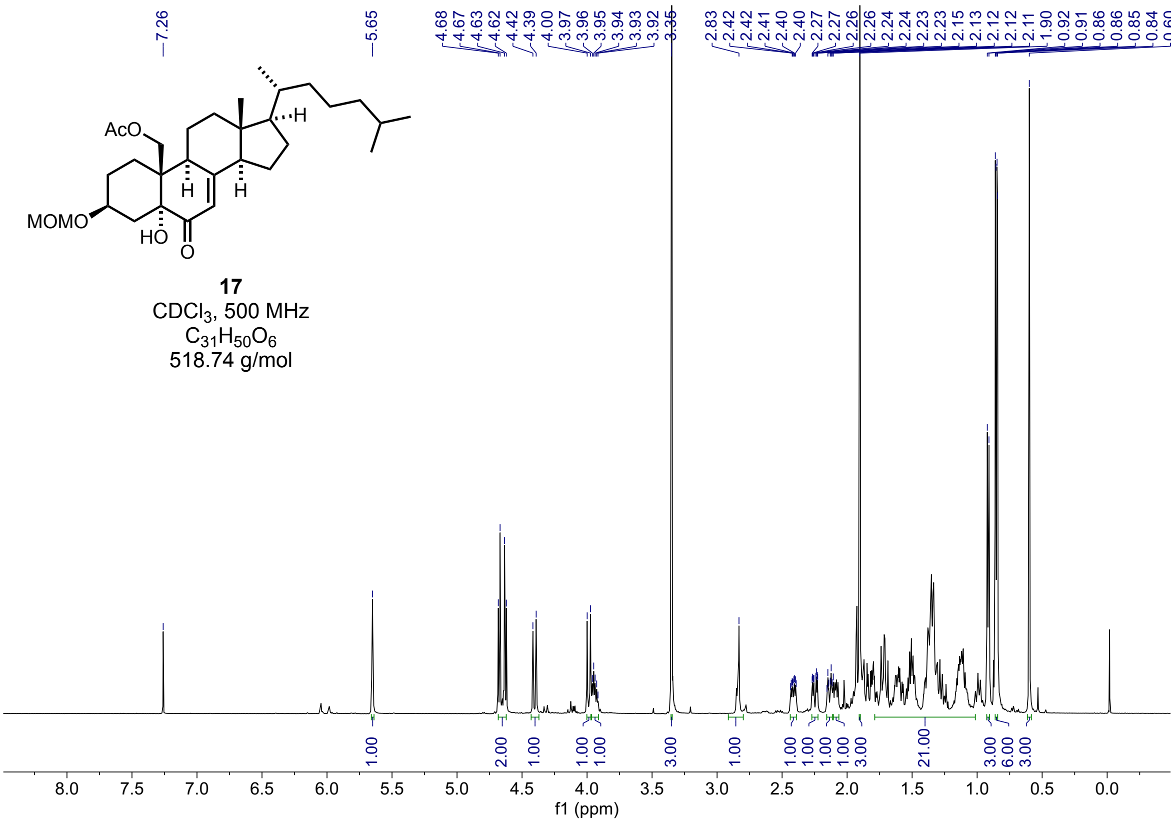


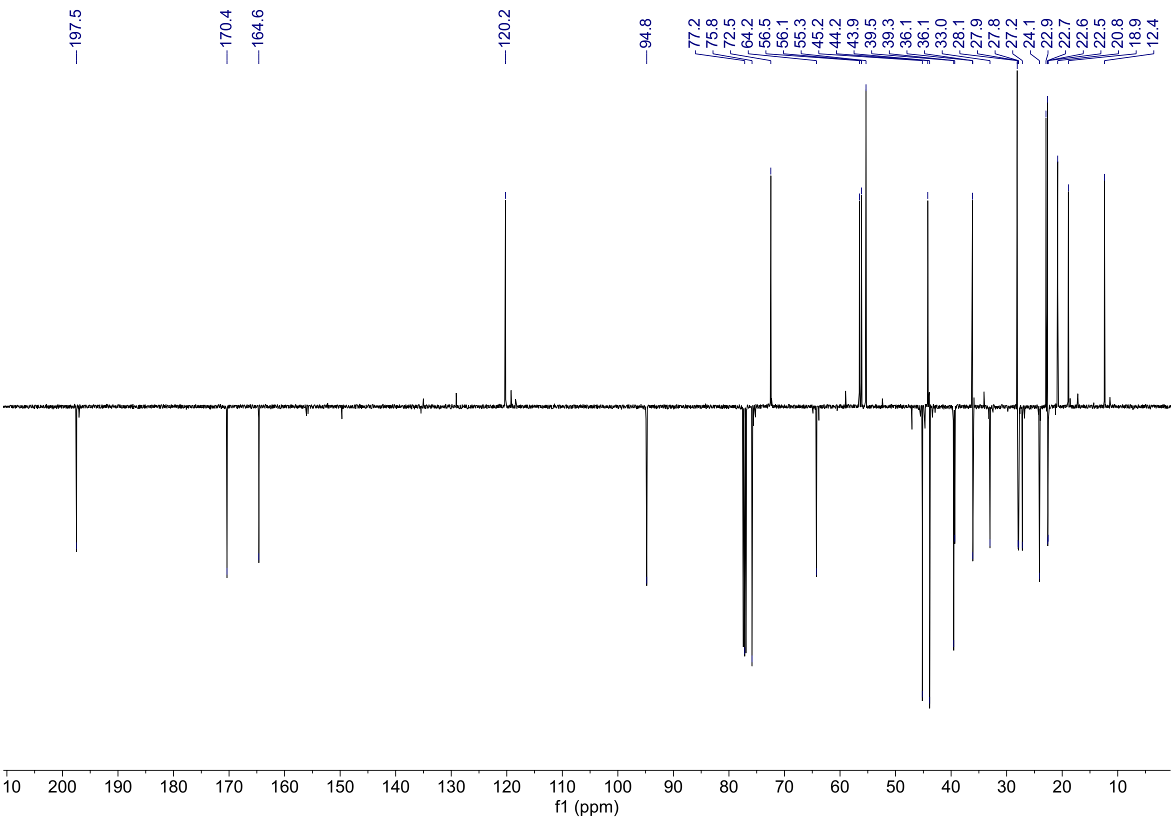


**Figure SI-16.** [1]H and [13]C NMR spectra of 3*β*-Methoxymethyloxy-5*α*-hydroxy-19-acetoxy-cholest-$\Delta^7$-en-6-one (**17**).

## 4.14 NMR spectra of 3*β*-Methoxymethyloxy-5*α*-hydroxy-8*β*,19-epoxy-cholestan-6-one (18)

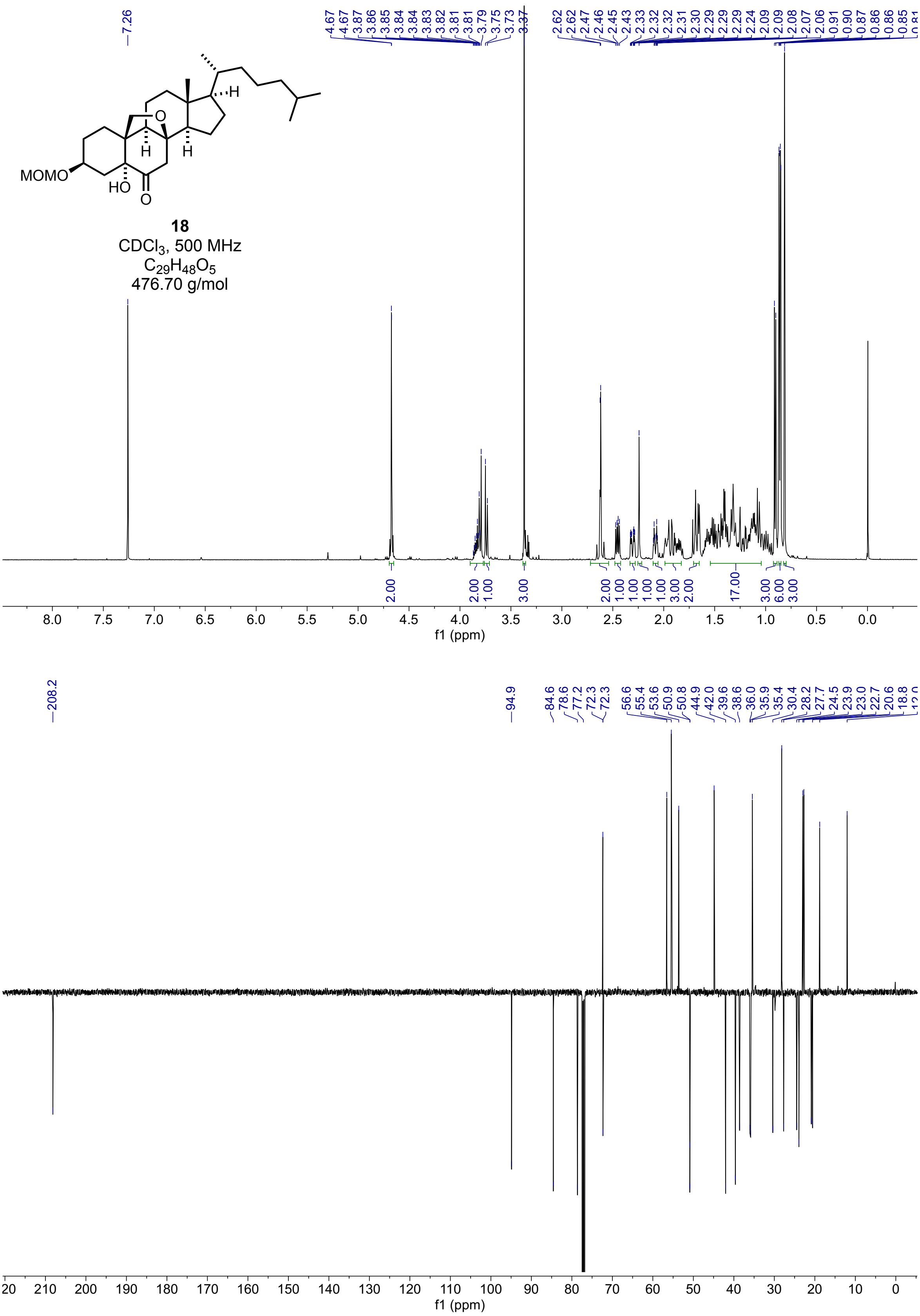


**Figure SI-17.** $^{1}H$ and $^{13}C$ NMR spectra of 3*β*-Methoxymethyloxy-5*α*-hydroxy-8*β*,19-epoxy-cholestan-6-one (**18**).

## 4.15 NMR spectra of 3*β*,19-Diacetoxy-cholest-Δ⁵-ene (19)

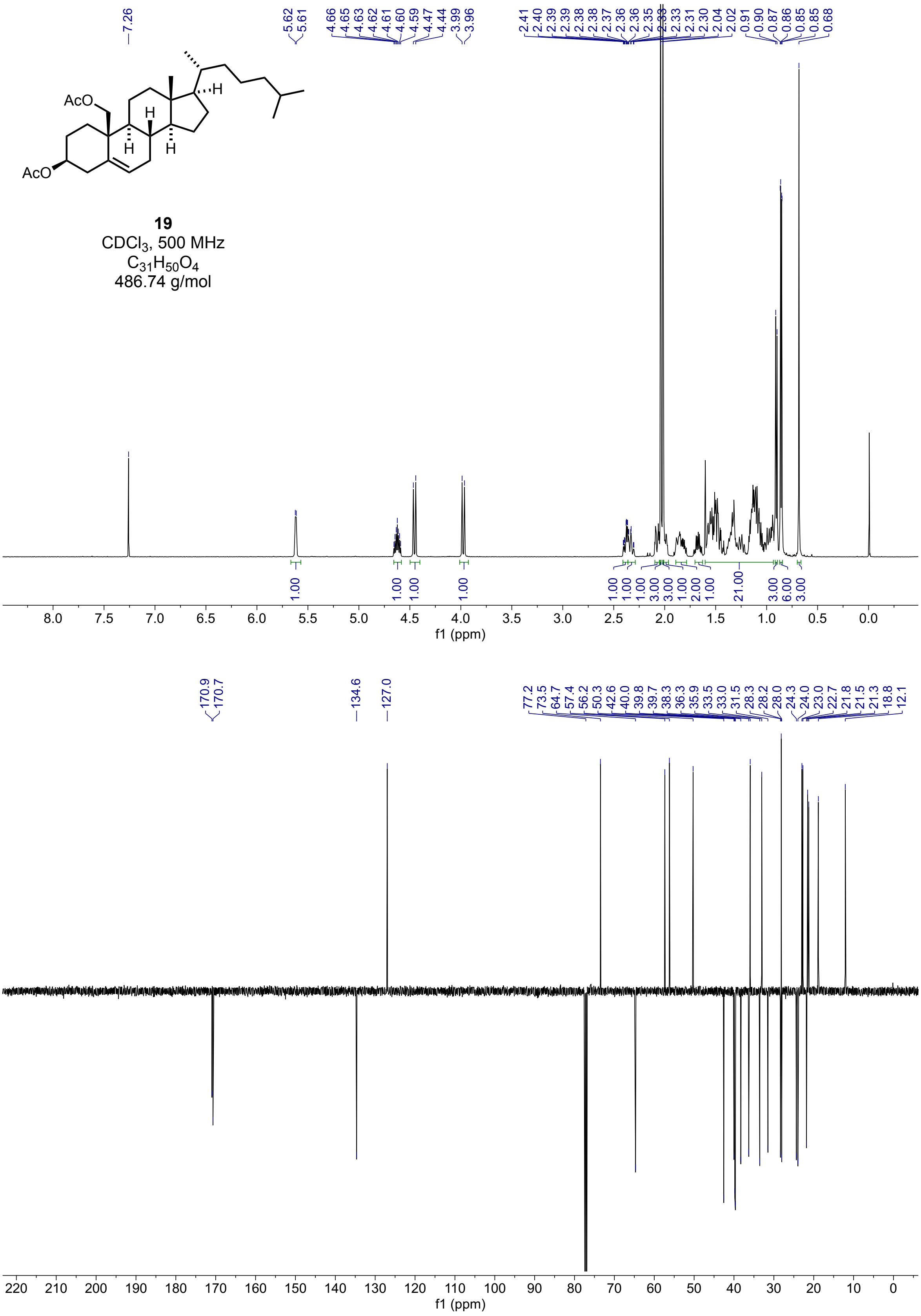


**Figure SI-18.** $^{1}$H and $^{13}$C NMR spectra of 3*β*,19-Diacetoxy-cholest-Δ$^{5}$-ene (**19**).

## 4.16 NMR spectra of 3$\beta$,19-Diacetoxy-cholestane-5$\alpha$,6$\alpha$-diol (SI-7)

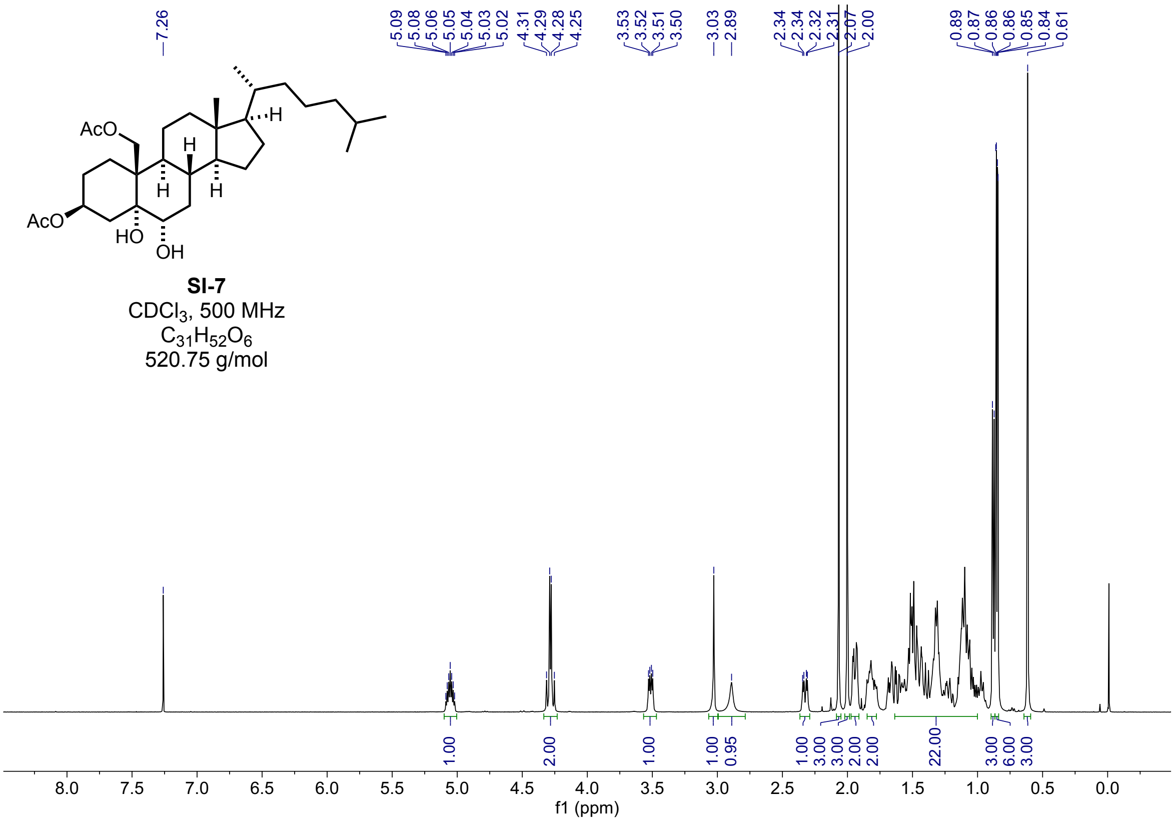


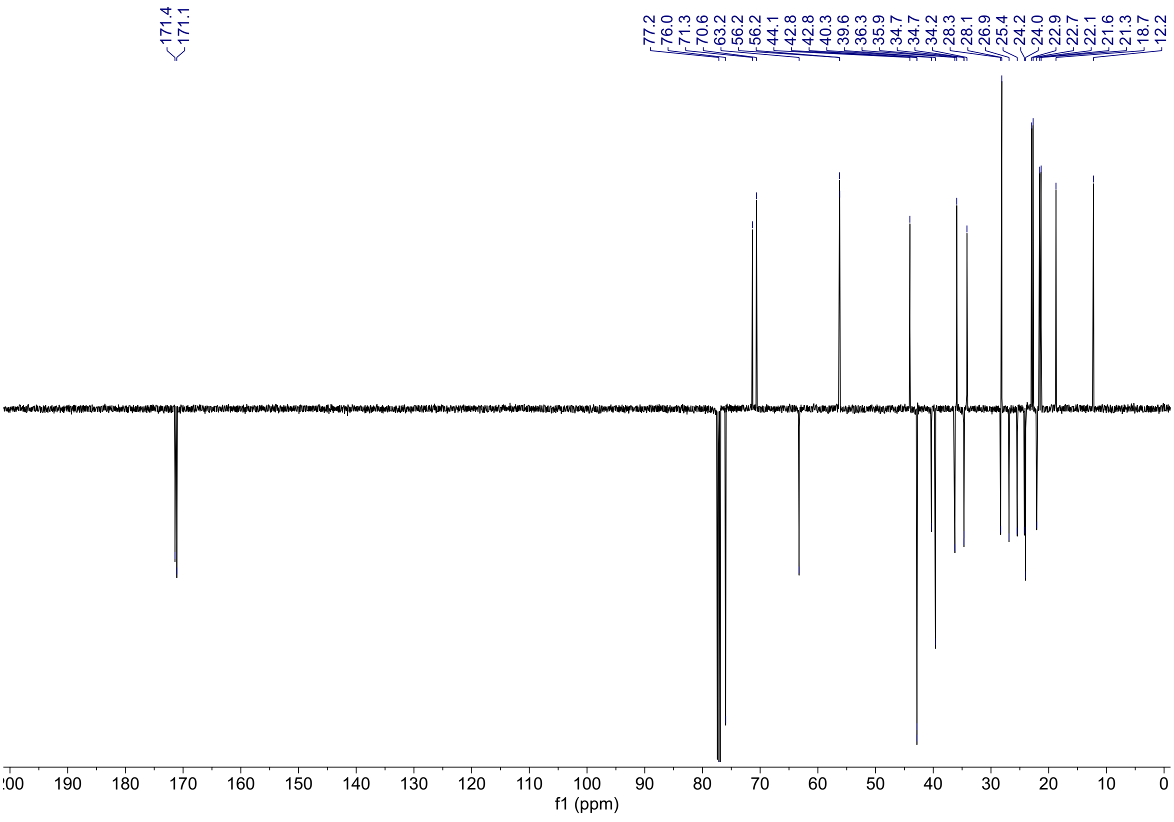


**Figure SI-19.** $^{1}H$ and $^{13}C$ NMR spectra of 3$\beta$,19-Diacetoxy-cholestane-5$\alpha$,6$\alpha$-diol (**SI-7**).

## 4.17 NMR spectra of 3$\beta$,19-Diacetoxy-5$\alpha$-hydroxy-cholestan-6-one (20)

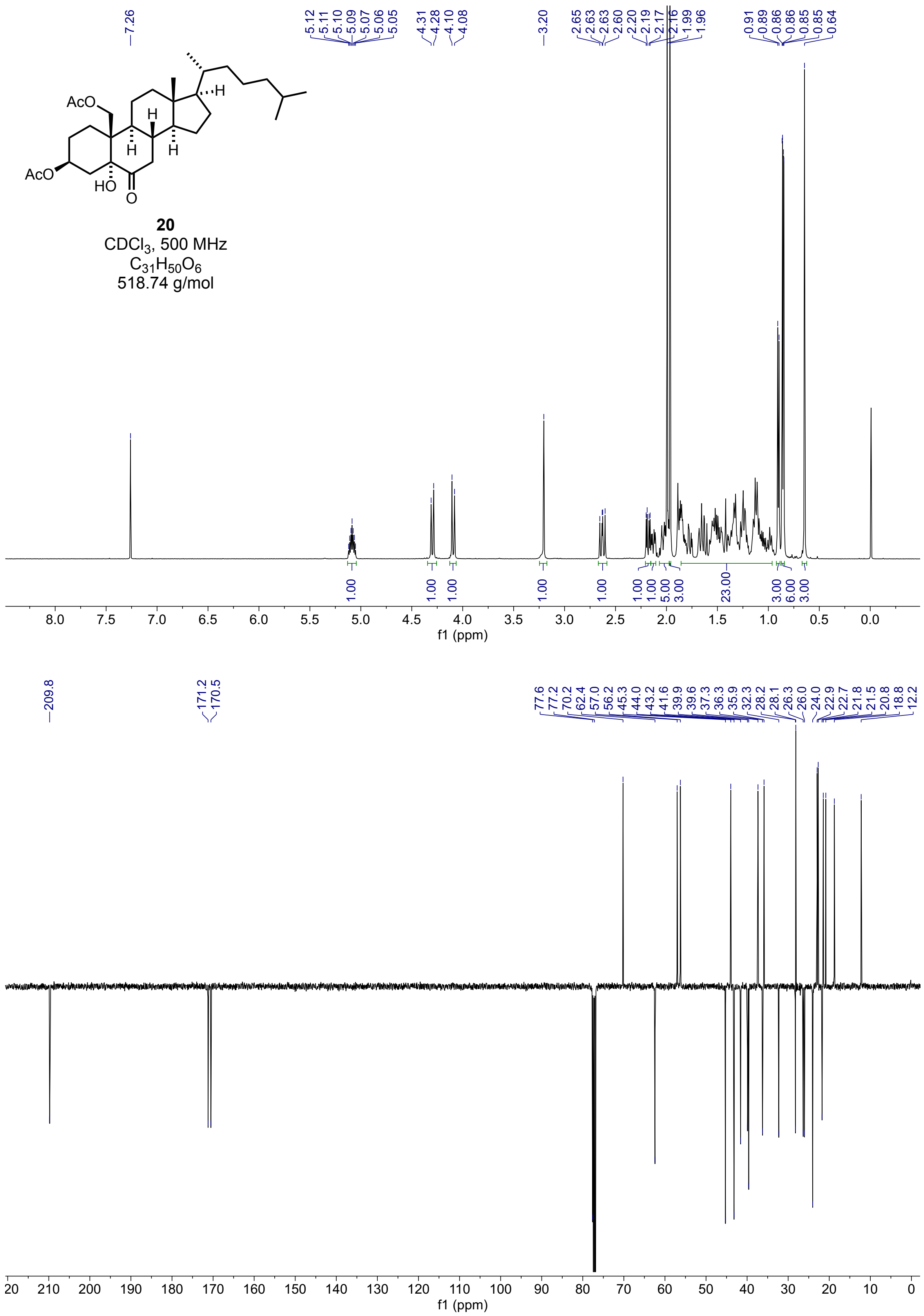


**Figure SI-20.** $^{1}H$ and $^{13}C$ NMR spectra of 3$\beta$,19-Diacetoxy-5$\alpha$-hydroxy-cholestan-6-one (**20**).

## 4.18 NMR spectra of 3$\beta$,19-Diacetoxy-5$\alpha$-hydroxy-7$\alpha$-bromo-cholestan-6-one (SI-8)

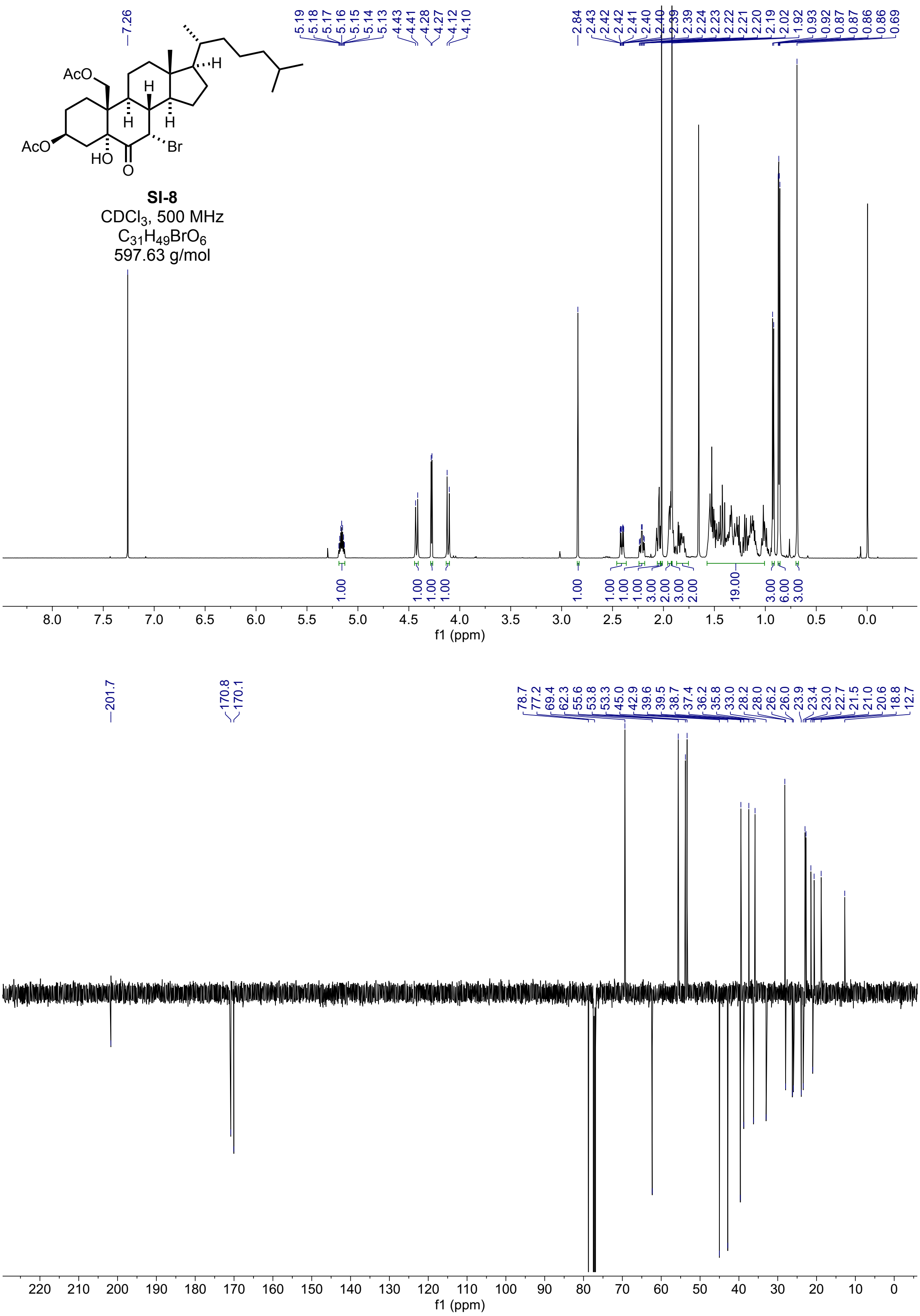


**Figure SI-21.** [1]H and [13]C NMR spectra of 3$\beta$,19-Diacetoxy-5$\alpha$-hydroxy-7$\alpha$-bromo-cholestan-6-one (**SI-8**).

## 4.19 NMR spectra of 3*β*,19-Diacetoxy-5*α*-hydroxy-cholestan-Δ[7]-en-6-one (21)

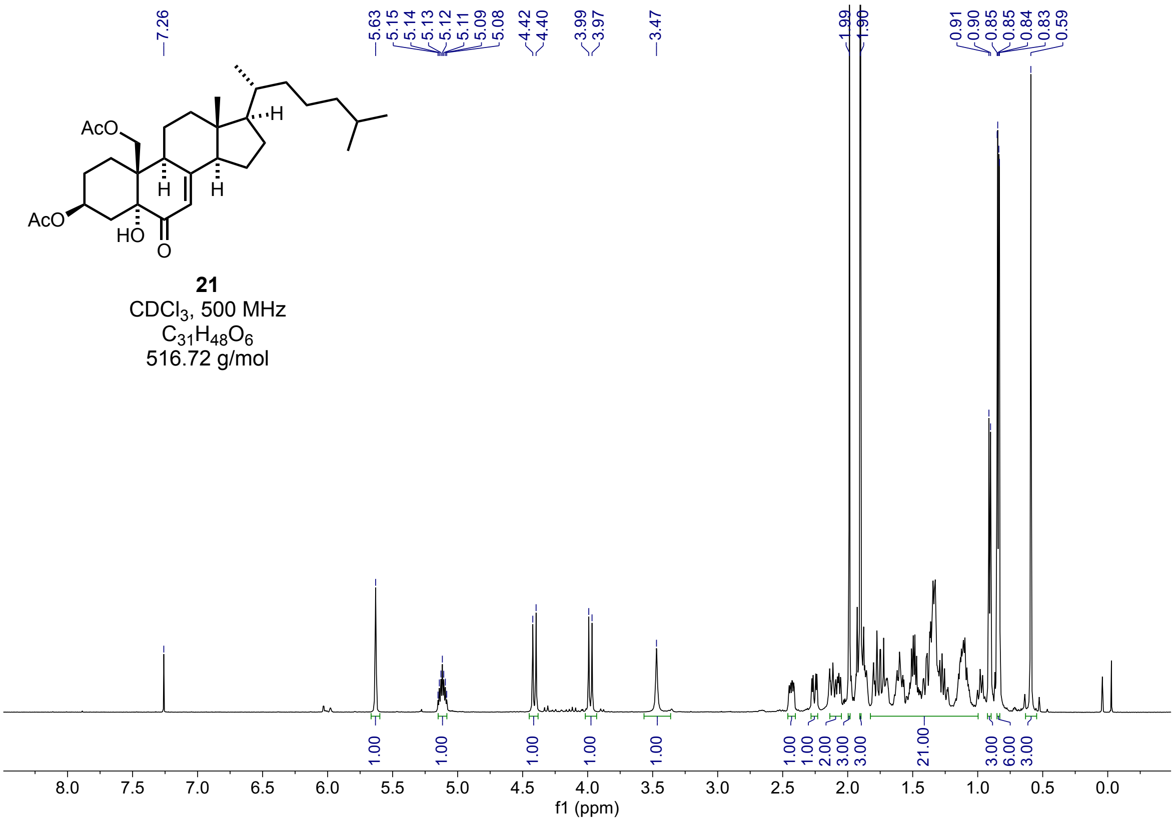


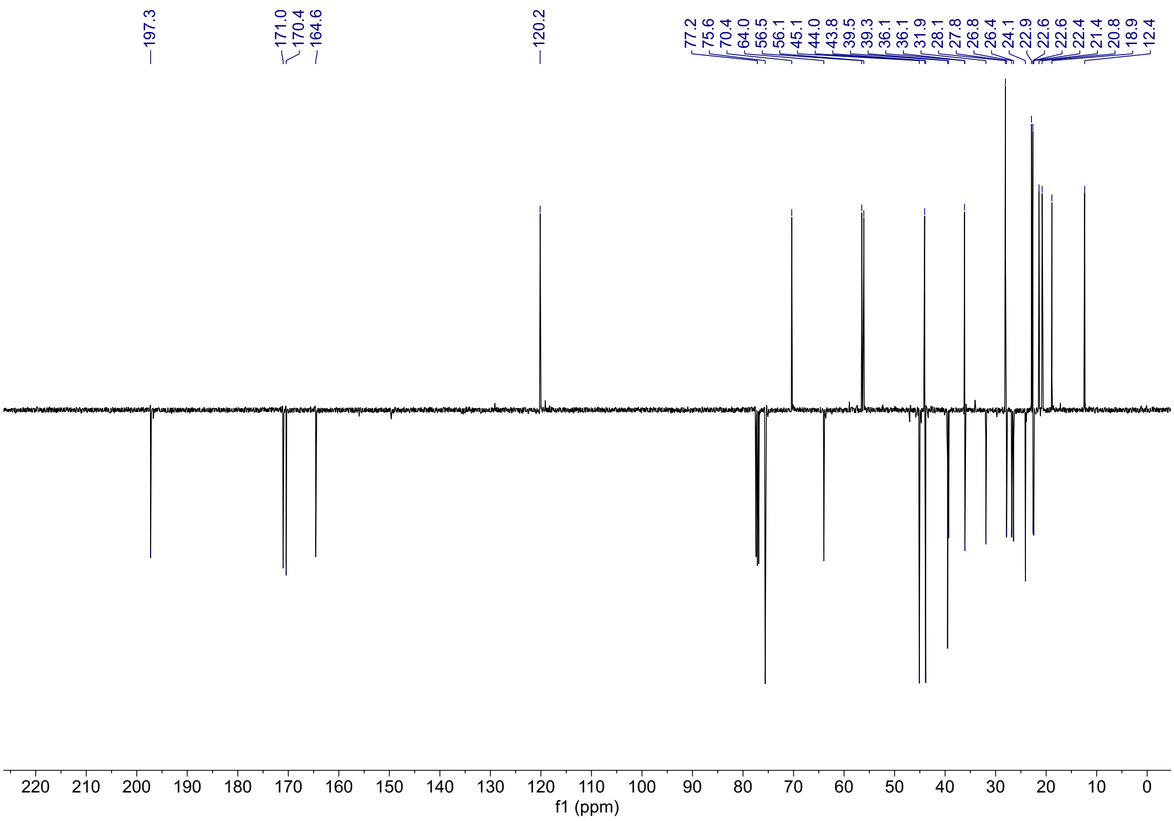


**Figure SI-22.** $^{1}$H and $^{13}$C NMR spectra of 3*β*,19-Diacetoxy-5*α*-hydroxy-cholestan-Δ[7]-en-6-one (**21**).

## 4.20 NMR spectra of 3*β*,5*α*-Dihydroxy-8*β*,19-epoxy-cholestan-6-one (4)

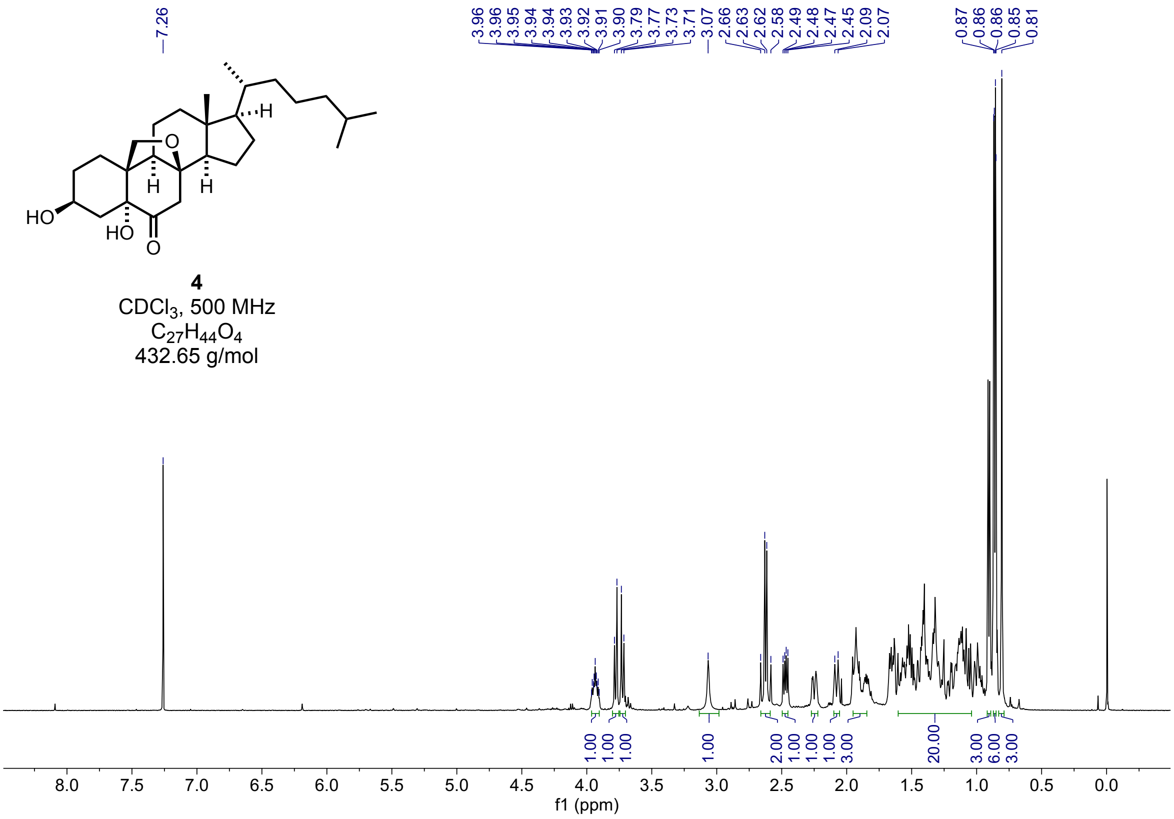


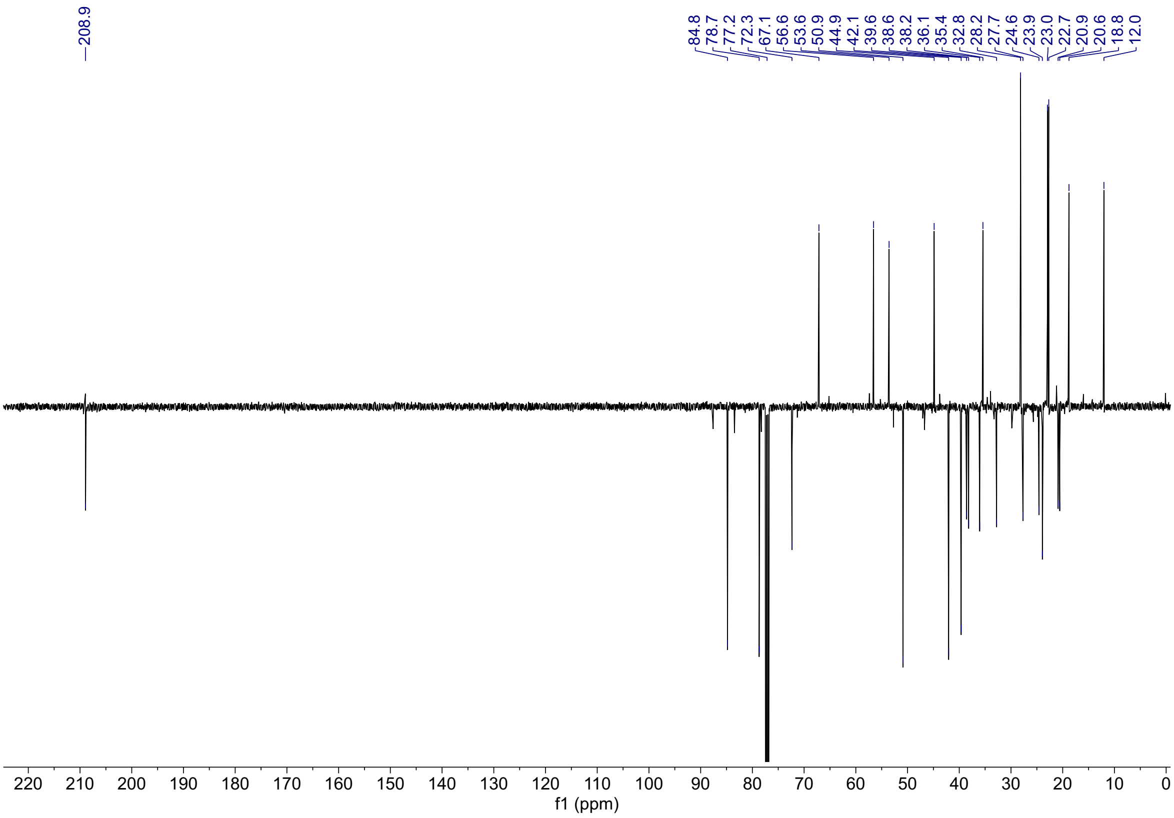


**Figure SI-23.** $^{1}H$ and $^{13}C$ NMR spectra of 3*β*,5*α*-Dihydroxy-8*β*,19-epoxy-cholestan-6-one (**4**).

## 4.21 NMR spectra of 3*β*-Acetoxy-19-methoxymethyloxy-cholestan-Δ[5]-ene (SI-9)

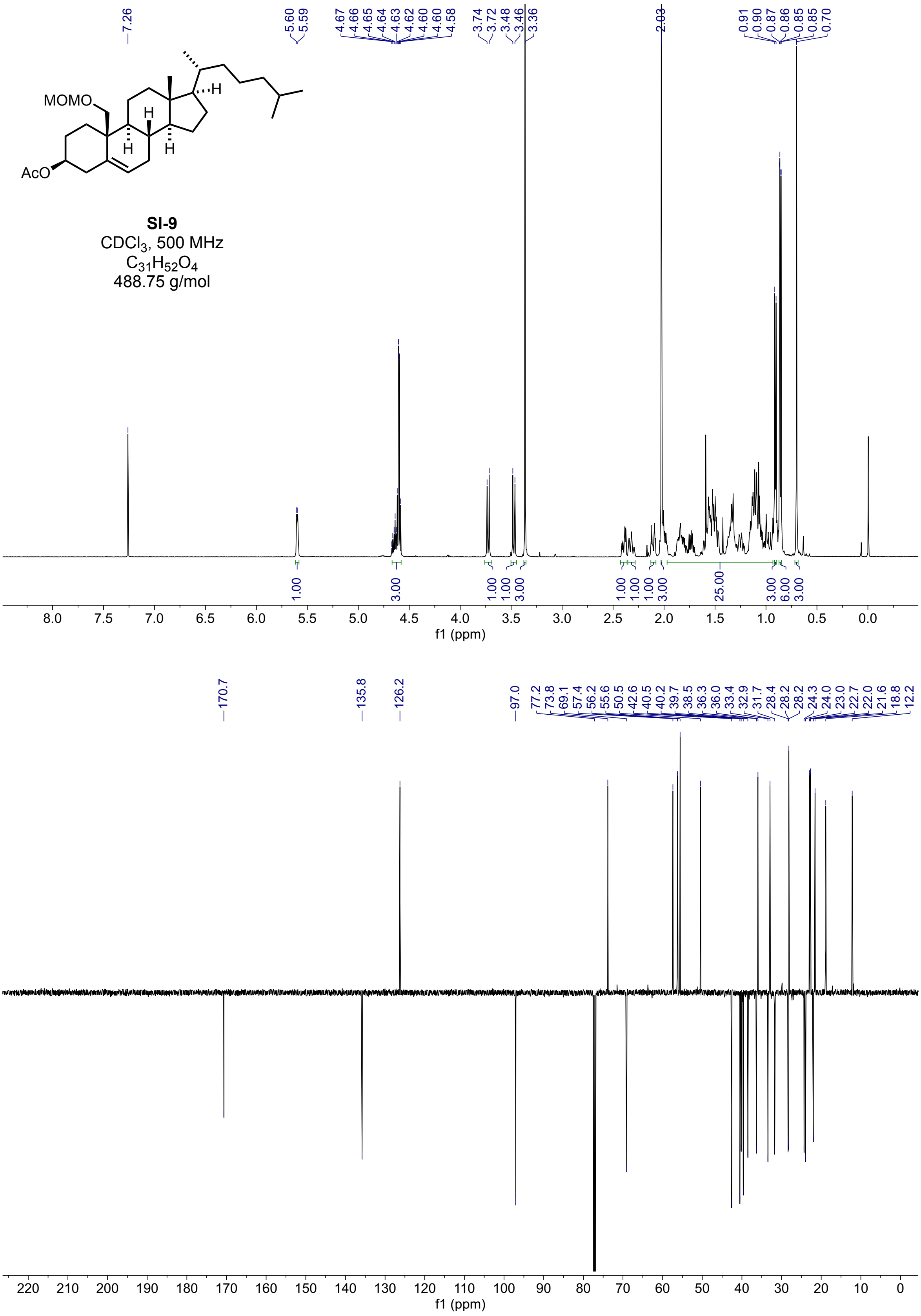


**Figure SI-24.** $^{1}H$ and $^{13}C$ NMR spectra of 3*β*-Acetoxy-19-methoxymethyloxy-cholestan-Δ[5]-ene (**SI-9**).

## 4.22 NMR spectra of 3*β*-Acetoxy-5*α*-hydroxy-19-methoxymethyloxy-cholestan-6-one (SI-10)

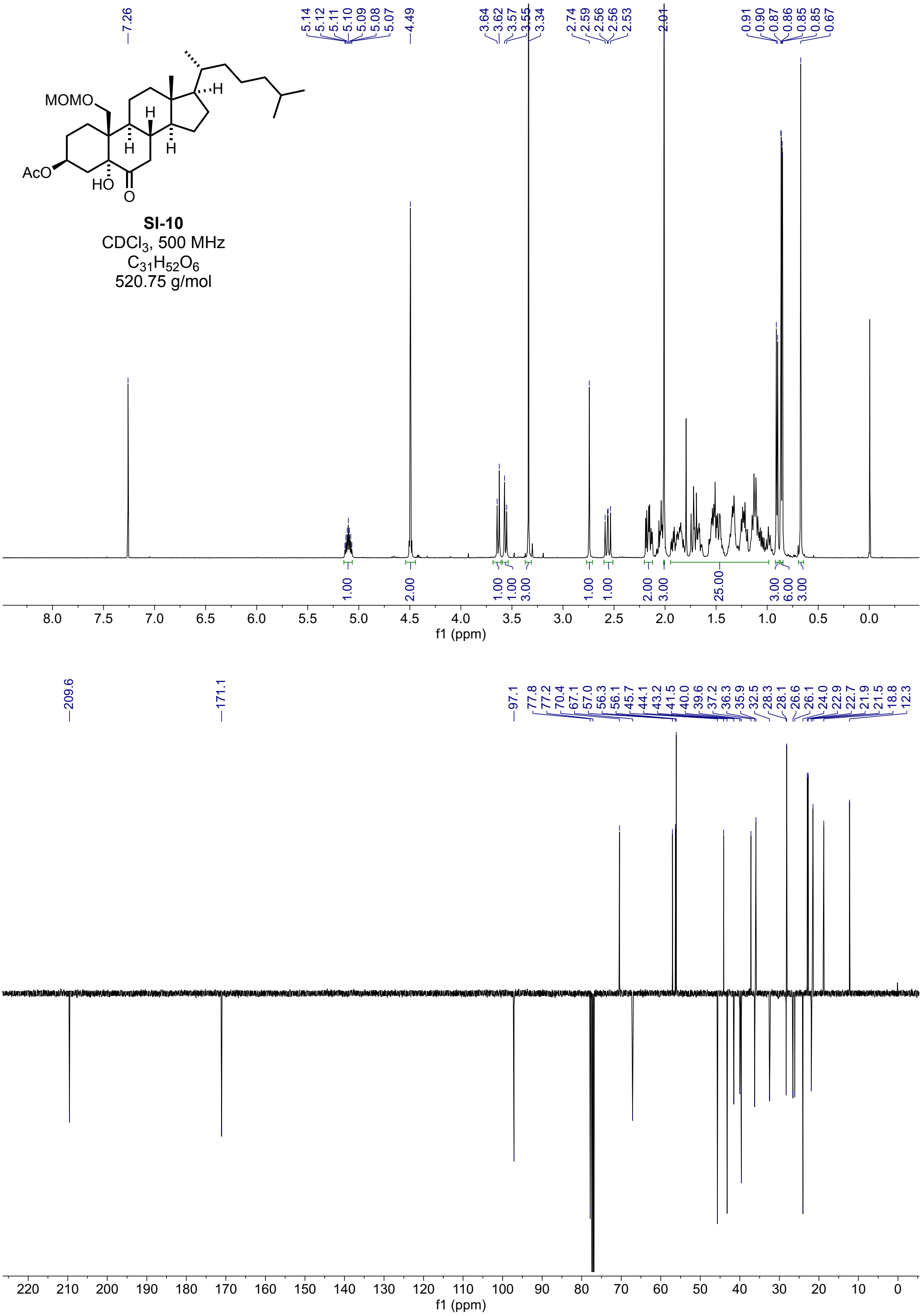


**Figure SI-25.** $^{1}$H and $^{13}$C NMR spectra of 3*β*-Acetoxy-5*α*-hydroxy-19-methoxymethyloxy-cholestan-6-one (**SI-10**).

## 4.23 NMR spectra of 3*β*-Acetoxy-7*α*-bromo-5*α*,6*α*-dihydroxy-6*β*,19-epoxy-cholestane (23)

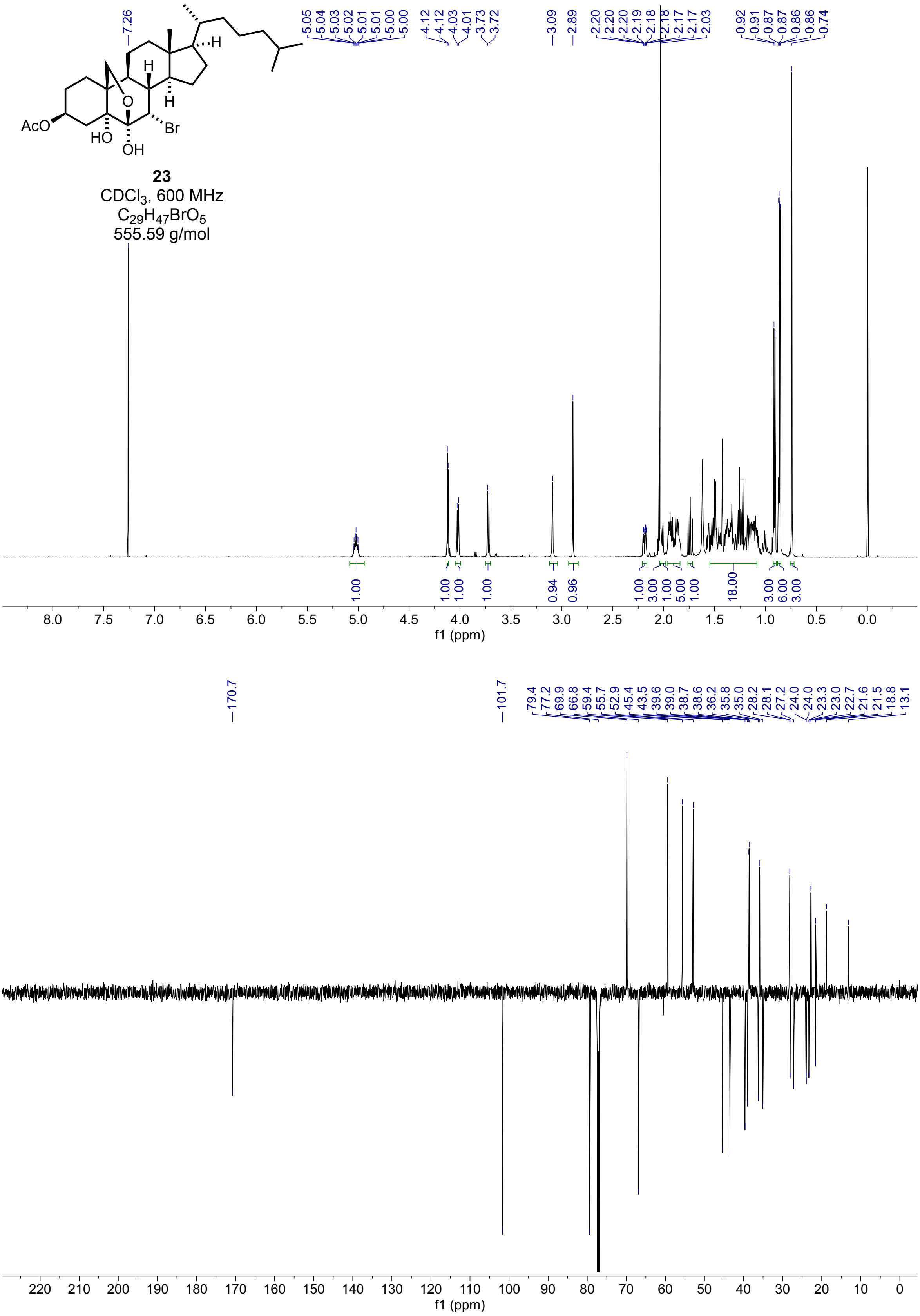


**Figure SI-26.** $^{1}$H and $^{13}$C NMR spectra of 3*β*-Acetoxy-7*α*-bromo-5*α*,6*α*-dihydroxy-6*β*,19-epoxy-cholestane (**23**).

## 4.24 NMR spectra of 3*β*-Acetoxy-5*α*-hydroxy-7*β*,19-epoxy-cholestan-6-one (SI-11)

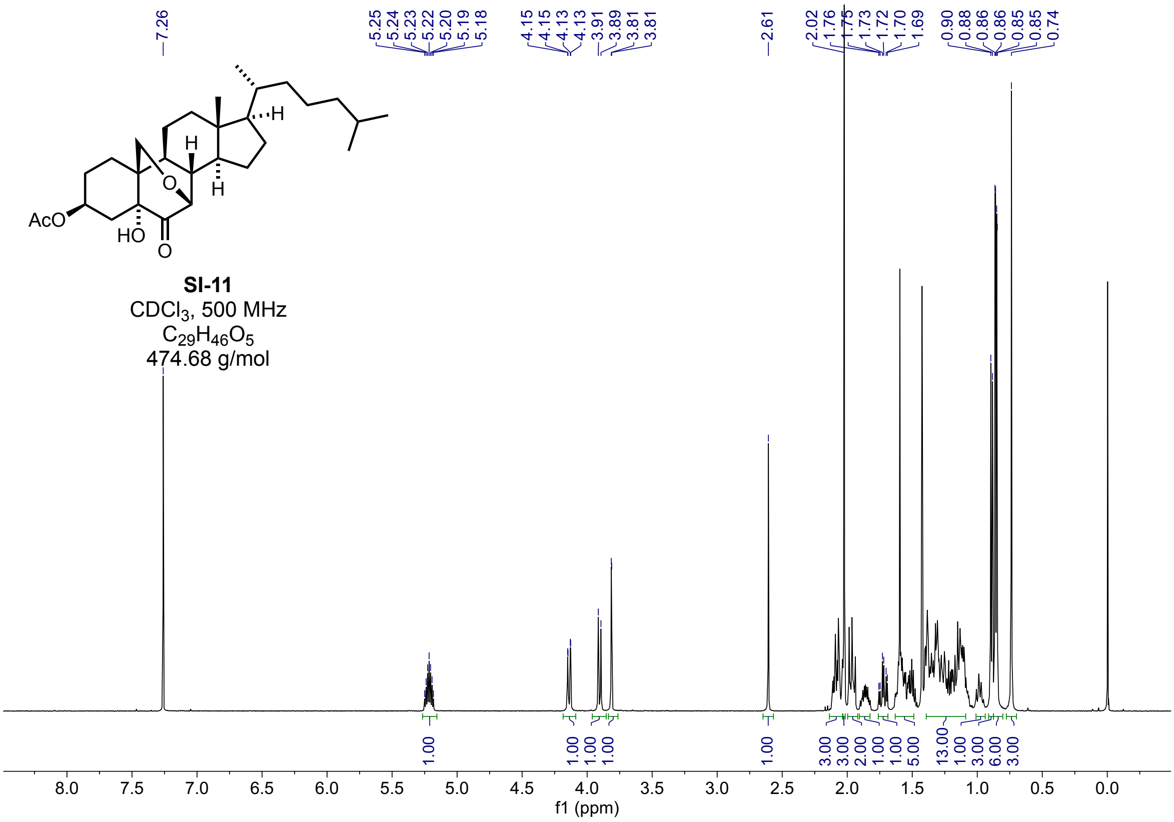


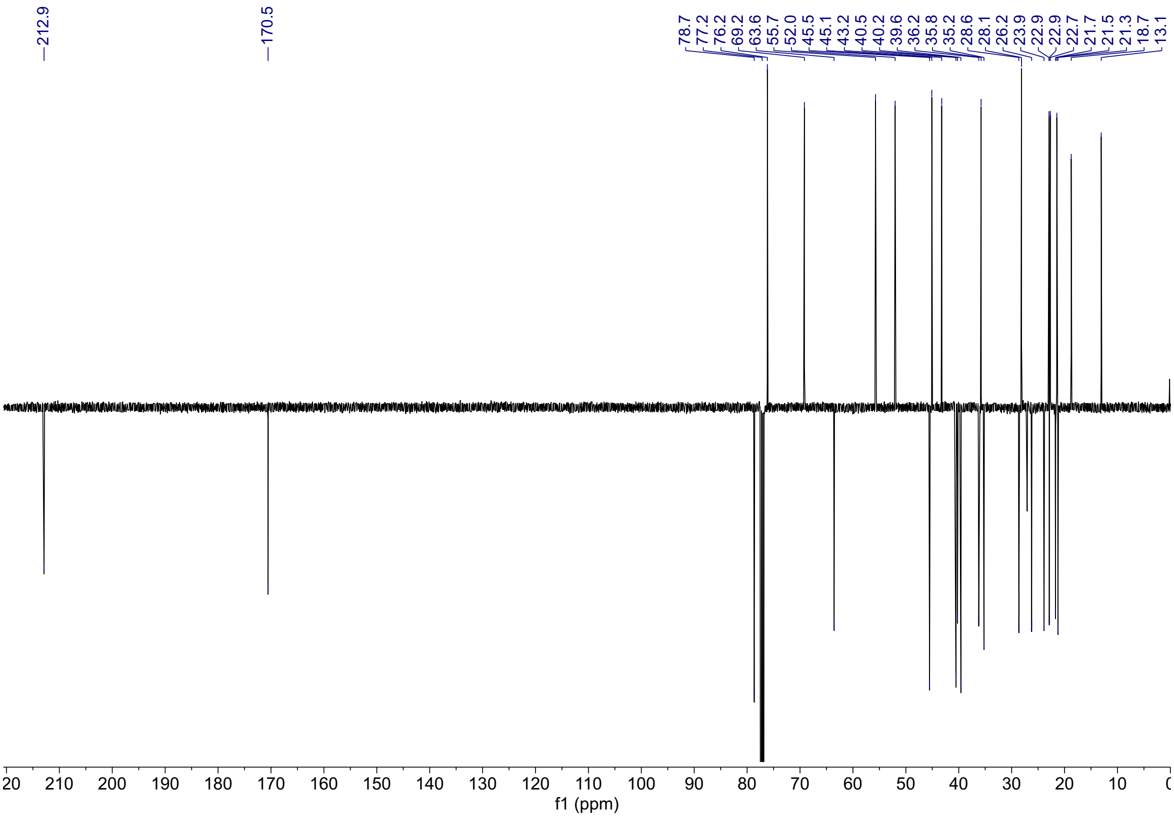


**Figure SI-27.** $^{1}$H- and $^{13}$C-NMR spectra of 3*β*-Acetoxy-5*α*-hydroxy-7*β*,19-epoxy-cholestan-6-one (**SI-11**).

## 4.25 NMR spectra of 3*β*,5*α*-Dihydroxy-7*β*,19-epoxy-cholestan-6-one (*iso*-4)

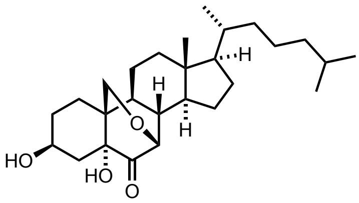


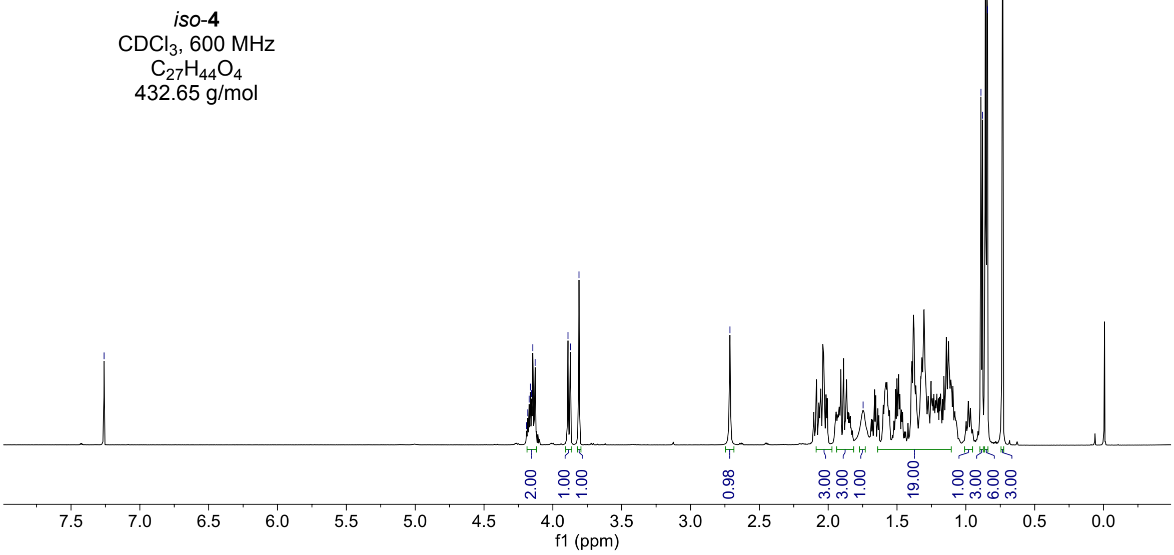


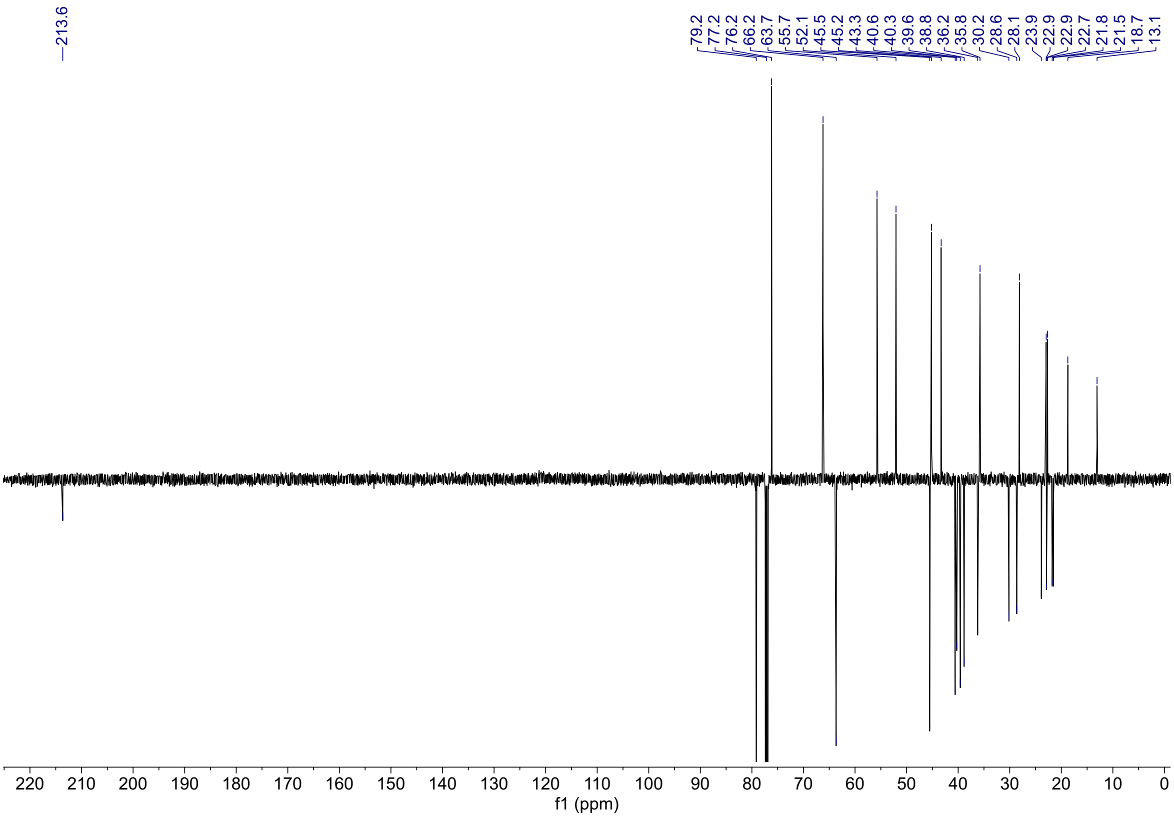


**Figure SI-28.** $^{1}H$ and $^{13}C$ NMR spectra of 3*β*,5*α*-Dihydroxy-7*β*,19-epoxy-cholestan-6-one (***iso*-4**).

## 4.26 NMR spectra of 5$\alpha$-Hydroxy-8$\beta$,19-epoxy-cholestan-6-one 3$\beta$-sulfonic acid (22)

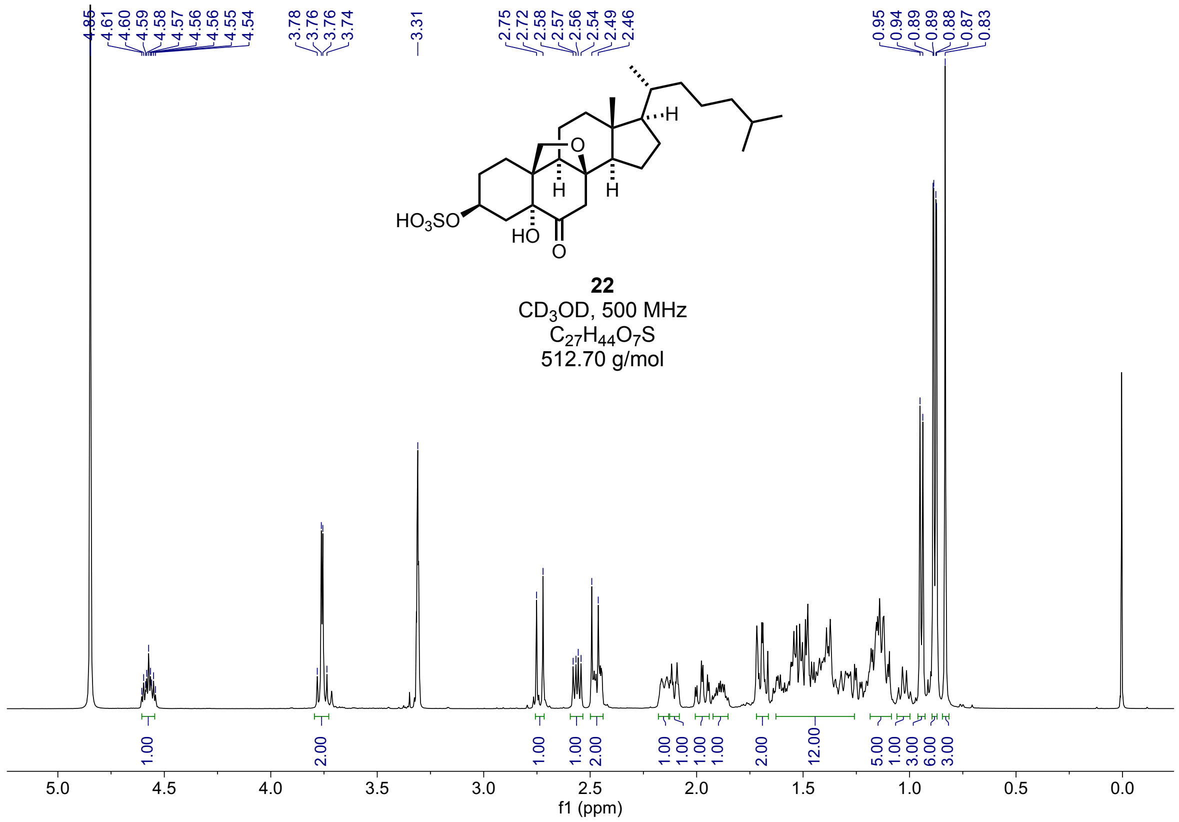


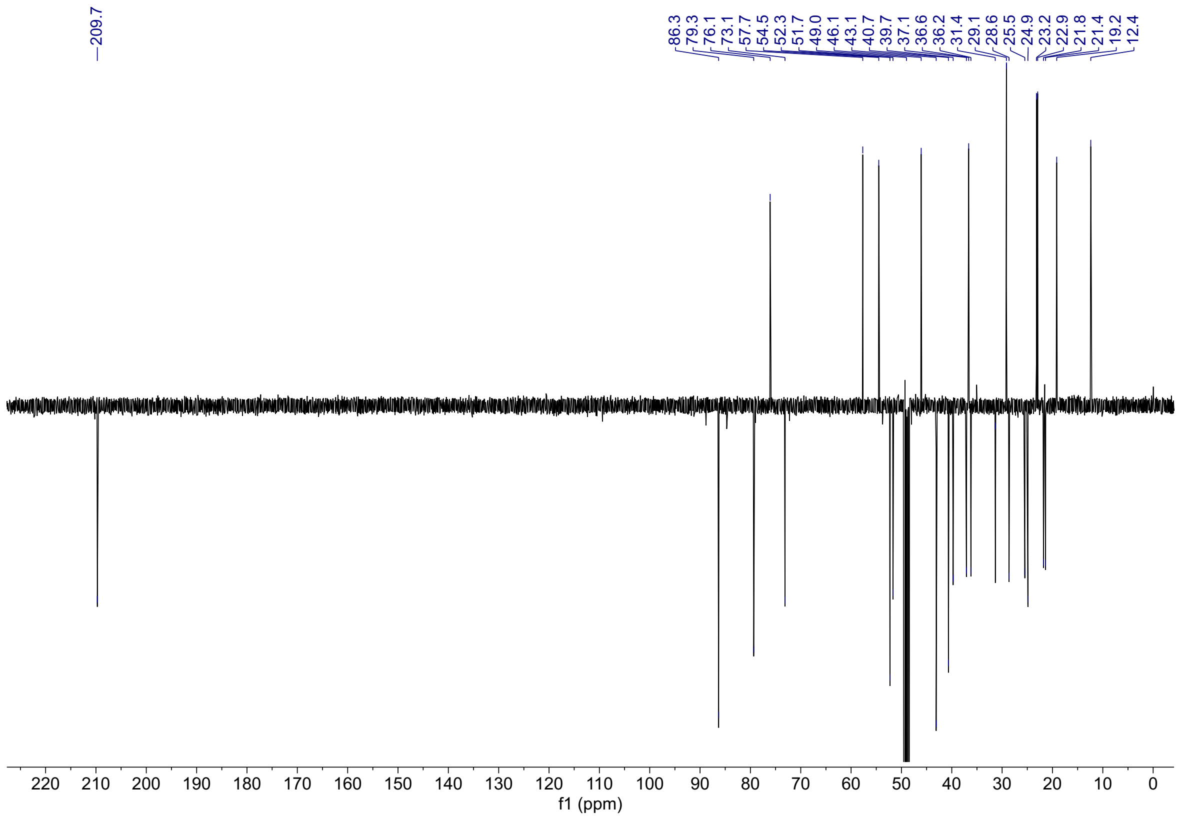


**Figure SI-29.** [1]H and [13]C NMR spectra of 5$\alpha$-Hydroxy-8$\beta$,19-epoxy-cholestan-6-one 3$\beta$-sulfonic acid (**22**).

## 5. Comparison of synthetic eurysterol A with eurysterol A isolated from nature [5]

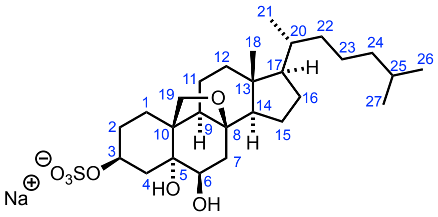


eurysterol A (**1**)

**Table SI-1.** Comparison of $^1$H NMR spectroscopic data.

| Position | Natural eurysterol A[5] (CD$_3$OD, 400 MHz) | Synthetic eurysterol A (CD$_3$OD, 500 MHz) | Δδ |
|---|---|---|---|
| 1*α* | 1.85 m | 1.85 m | 0.00 |
| 1*β* | 1.56 m | 1.55 m | -0.01 |
| 2*α* | 2.13 m | 2.14 m | +0.01 |
| 2*β* | 1.18 m | 1.18 m | 0.00 |
| 3 | 4.63 tt (11.6, 4.4 Hz) | 4.64 tt (11.4, 4.4 Hz) | +0.01 |
| 4*α* | 2.05 m | 2.05 m | 0.00 |
| 4*β* | 2.23 dd (13.6, 11.6 Hz) | 2.24 dd (13.7, 11.7 Hz) | +0.01 |
| 5 | | | |
| 6 | 3.42 dd (4.0 Hz) | 3.43 d (4.3 Hz) | +0.01 |
| 7*α* | 1.87 m | 1.87 m | 0.00 |
| 7*β* | 1.87 m | 1.87 m | 0.00 |
| 8 | | | |
| 9 | 2.03 m | 2.02 m | -0.01 |
| 10 | | | |
| 11*α* | 1.39 m | 1.39 m | 0.00 |
| 11*β* | 1.41 m | 1.41 m | 0.00 |
| 12*α* | 1.14 m | 1.14 m | 0.00 |
| 12*β* | 2.04 m | 2.04 m | 0.00 |
| 13 | | | |
| 14 | 1.32 m | 1.32 m | 0.00 |
| 15*α* | 1.66 m | 1.67 m | +0.01 |
| 15*β* | 1.49 m | 1.49 m | 0.00 |
| 16*α* | 1.87 m | 1.87 m | 0.00 |
| 16*β* | 1.30 m | 1.30 m | 0.00 |
| 17 | 1.09 m | 1.09 m | 0.00 |
| 18 | 0.84 s | 0.85 s | +0.01 |
| 19*α* | 3.63 d (8.4 Hz) | 3.64 d (8.5 Hz) | +0.01 |
| 19*β* | 4.33 d (8.4 Hz) | 4.33 d (8.5 Hz) | 0.00 |
| 20 | 1.39 m | 1.39 m | 0.00 |
| 21 | 0.92 d (6.4 Hz) | 0.93 d (6.5 Hz) | +0.01 |
| 22 | 1.01 m | 1.01 m | 0.00 |
| 23 | 1.37 m | 1.37 m | 0.00 |
| 24 | 1.14 m | 1.14 m | 0.00 |
| 25 | 1.52 m | 1.53 m | +0.01 |
| 26 | 0.87 d (6.4 Hz) | 0.88 dd (6.6, 1.9 Hz) | +0.01 |
| 27 | 0.87 d (6.4 Hz) | 0.88 dd (6.6, 1.9 Hz) | +0.01 |

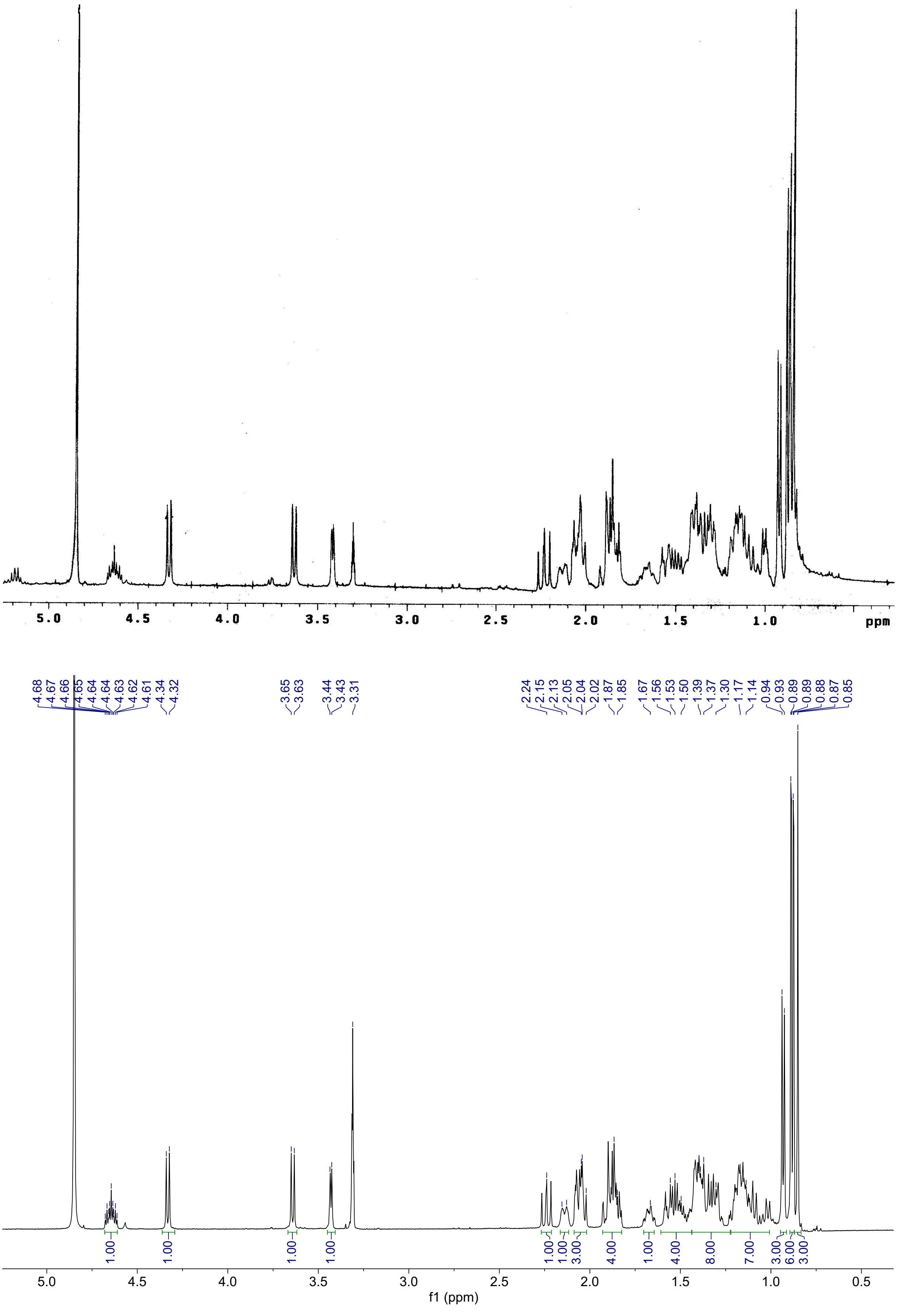

**Figure SI-30.** $^{1}$H NMR spectra comparison of natural and synthetic eurysterol A.[5]

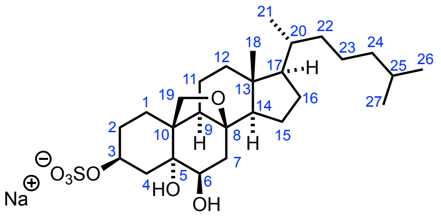


eurysterol A (**1**)

**Table SI-2.** Comparison of $^{13}C$ NMR spectroscopic data.

| Position | Natural eurysterol A[5] (CD3OD, 100 MHz) | Synthetic eurysterol A (CD3OD, 125 MHz) | Δδ |
|---|---|---|---|
| 1 | 26.4 | 26.3 | -0.1 |
| 2 | 31.8 | 31.7 | -0.1 |
| 3 | 76.6 | 76.7 | +0.1 |
| 4 | 39.9 | 39.9 | 0.0 |
| 5 | 77.3 | 77.4 | +0.1 |
| 6 | 75.6 | 75.6 | 0.0 |
| 7 | 42.6 | 42.6 | 0.0 |
| 8 | 85.5 | 85.6 | 0.0 |
| 9 | 46.5 | 46.5 | 0.0 |
| 10 | 50.3 | 50.3 | 0.0 |
| 11 | 21.6 | 21.6 | 0.0 |
| 12 | 40.0 | 40.0 | 0.0 |
| 13 | 42.8 | 42.8 | 0.0 |
| 14 | 54.8 | 54.8 | 0.0 |
| 15 | 21.6 | 21.5 | -0.1 |
| 16 | 28.8 | 28.7 | -0.1 |
| 17 | 57.8 | 57.8 | 0.0 |
| 18 | 12.6 | 12.5 | -0.1 |
| 19 | 72.2 | 72.2 | 0.0 |
| 20 | 36.7 | 36.7 | 0.0 |
| 21 | 19.3 | 19.2 | -0.1 |
| 22 | 37.2 | 37.1 | -0.1 |
| 23 | 25.0 | 24.9 | -0.1 |
| 24 | 40.7 | 40.7 | 0.0 |
| 25 | 29.2 | 29.1 | -0.1 |
| 26 | 23.0 | 22.9 | -0.1 |
| 27 | 23.2 | 23.2 | 0.0 |

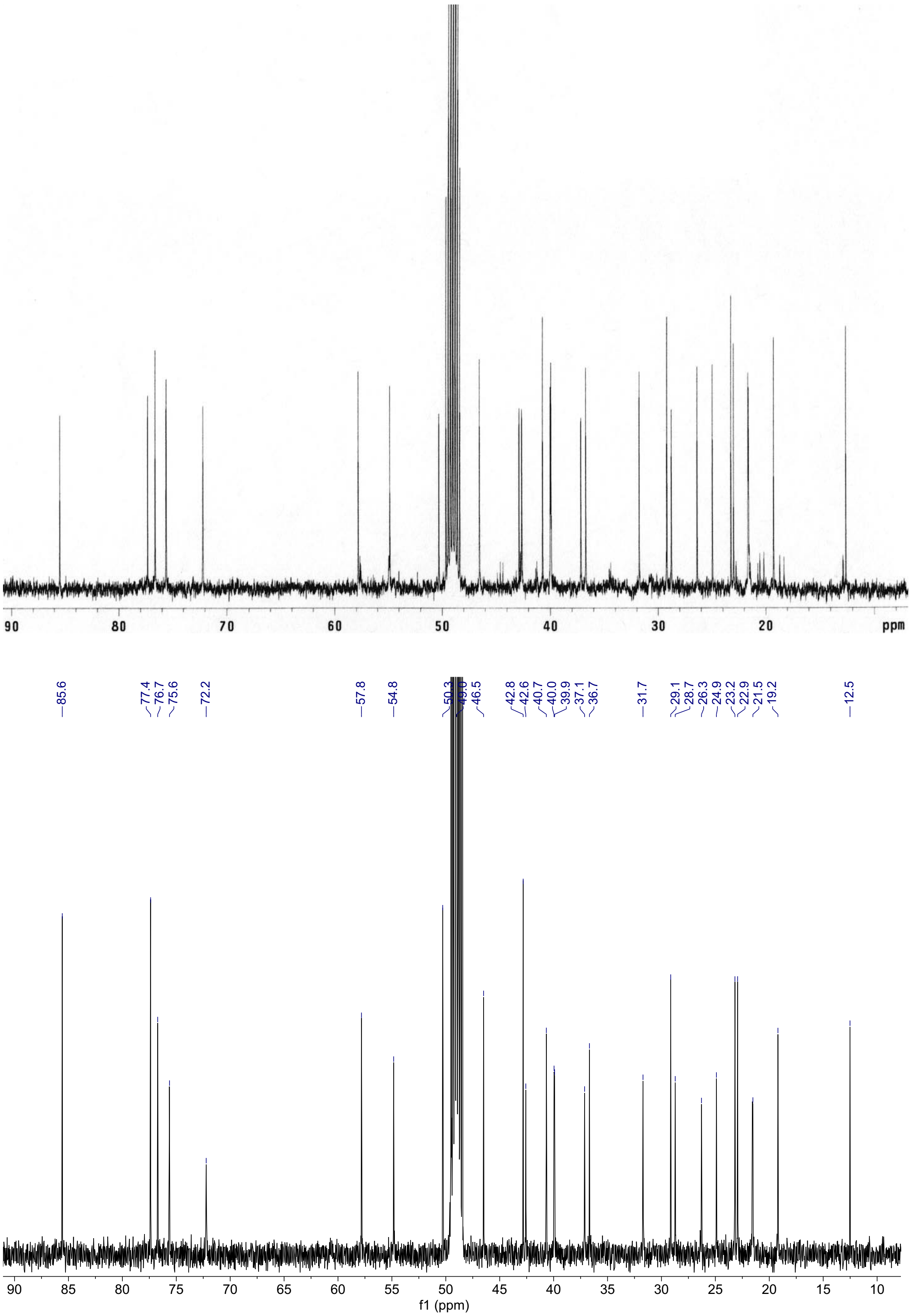


**Figure SI-31.** $^{13}$C NMR spectra comparison of natural (top) and synthetic (bottom) eurysterol A.[5]

## 6. X-ray Crystallographic Data

### 6.1 X-ray Crystallographic Data and Structure Refinement of 8 (CCDC 1978300)

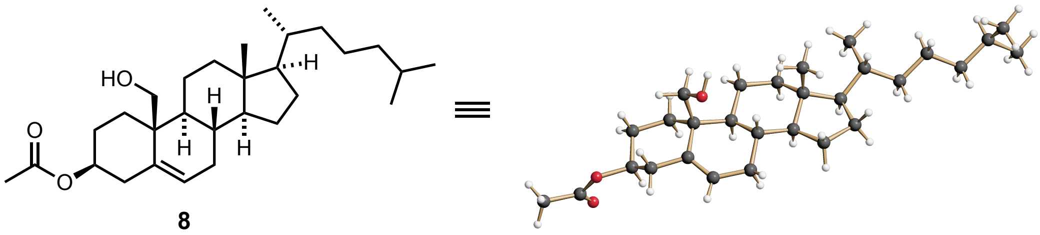


**8**

| | | |
|---|---|---|
| Empirical formula | $C_{29}H_{48}O_3$ | |
| Formula weight | 444.67 g/mol | |
| Temperature | 100(2) K | |
| Wavelength | 1.54178 Å | |
| Crystal system | Monoclinic | |
| Space group | $P2_1$ | |
| Unit cell dimensions | $a$ = 10.1291(3) Å | $\alpha$ = 90° |
| | $b$ = 7.6593(2) Å | $\beta$ = 104.6850(10)° |
| | $c$ = 17.1144(5) Å | $\gamma$ = 90° |
| Volume | 1284.39(6) $Å^3$ | |
| Z | 2 | |
| Density (calculated) | 1.150 $Mg/m^3$ | |
| Absorption coefficient | 0.553 $mm^{-1}$ | |
| F(000) | 492 | |
| Crystal size | 0.450 x 0.250 x 0.150 $mm^3$ | |
| Theta range for data collection | 4.512 to 72.107° | |
| Index ranges | -12<=h<=12, -9<=k<=9, -21<=l<=21 | |
| Reflections collected | 20262 | |
| Independent reflections | 5019 [R(int) = 0.0306] | |
| Completeness to theta = 67.679° | 99.6 % | |
| Absorption correction | Semi-empirical from equivalents | |
| Max. and min. transmission | 0.7536 and 0.5702 | |
| Refinement method | Full-matrix least-squares on $F^2$ | |
| Data / restraints / parameters | 5019 / 1 / 298 | |
| Goodness-of-fit on $F^2$ | 1.065 | |
| Final R indices [I>2sigma(I)] | R1 = 0.0307, wR2 = 0.0815 | |
| R indices (all data) | R1 = 0.0310, wR2 = 0.0820 | |
| Absolute structure parameter | 0.04(6) | |
| Extinction coefficient | n/a | |
| Largest diff. peak and hole | 0.260 and -0.177 e.$Å^{-3}$ | |

## 6.2 X-ray Crystallographic Data and Structure Refinement of 4 (CCDC 1978299)

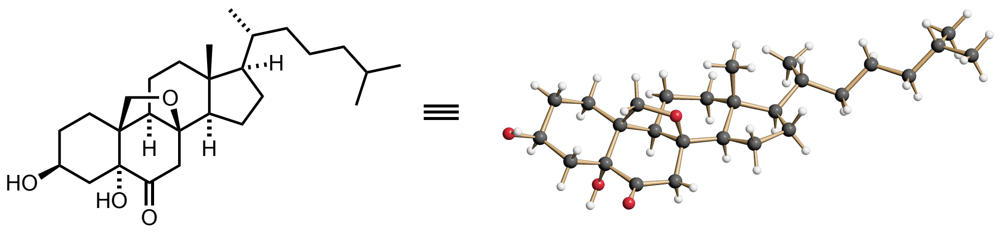

**4**

| | | |
|---|---|---|
| Empirical formula | $C_{27}H_{44}O_4$ | |
| Formula weight | 432.62 g/mol | |
| Temperature | 100(2) K | |
| Wavelength | 1.54178 Å | |
| Crystal system | Orthorhombic | |
| Space group | $P2_12_12$ | |
| Unit cell dimensions | $a$ = 9.3798(6) Å | $\alpha$ = 90° |
| | $b$ = 41.677(3) Å | $\beta$ = 90° |
| | $c$ = 6.1377(4) Å | $\gamma$ = 90° |
| Volume | 2399.4(3) $Å^3$ | |
| Z | 4 | |
| Density (calculated) | 1.198 $Mg/m^3$ | |
| Absorption coefficient | 0.612 $mm^{-1}$ | |
| F(000) | 952 | |
| Crystal size | 0.500 x 0.100 x 0.050 $mm^3$ | |
| Theta range for data collection | 4.243 to 72.179° | |
| Index ranges | -11<=h<=11, -51<=k<=48, -7<=l<=5 | |
| Reflections collected | 12783 | |
| Independent reflections | 4601 [R(int) = 0.0562] | |
| Completeness to theta = 67.679° | 98.8 % | |
| Absorption correction | Semi-empirical from equivalents | |
| Max. and min. transmission | 0.7536 and 0.5152 | |
| Refinement method | Full-matrix least-squares on $F^2$ | |
| Data / restraints / parameters | 4601 / 0 / 292 | |
| Goodness-of-fit on $F^2$ | 1.139 | |
| Final R indices [I>2sigma(I)] | R1 = 0.0640, wR2 = 0.1682 | |
| R indices (all data) | R1 = 0.0663, wR2 = 0.1694 | |
| Absolute structure parameter | 0.10(9) | |
| Extinction coefficient | n/a | |
| Largest diff. peak and hole | 0.566 and -0.315 e.$Å^{-3}$ | |

## 6.3 X-ray Crystallographic Data and Structure Refinement of 1 (CCDC 1982419)

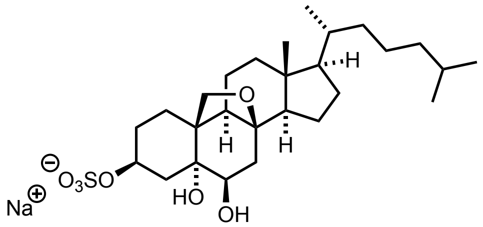


**1**

≡

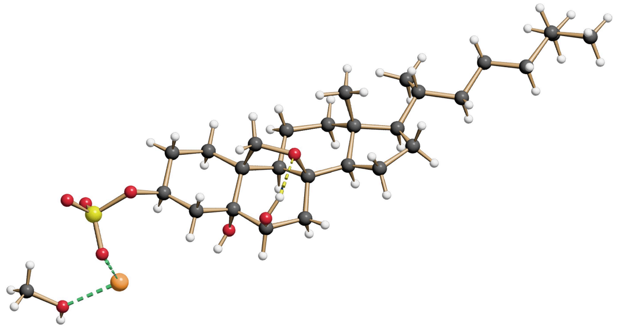

| | | |
|---|---|---|
| Empirical formula | $C_{28}H_{49}NaO_8S$ | |
| Formula weight | 568.72 g/mol | |
| Temperature | 100(2) K | |
| Wavelength | 1.54178 Å | |
| Crystal system | Monoclinic | |
| Space group | $P2_1$ | |
| Unit cell dimensions | $a$ = 7.0350(3) Å | $\alpha$ = 90° |
| | $b$ = 10.2328(5) Å | $\beta$ = 94.905(2)° |
| | $c$ = 20.2521(10) Å | $\gamma$ = 90° |
| Volume | 1452.56(12) $Å^3$ | |
| Z | 2 | |
| Density (calculated) | 1.300 $Mg/m^3$ | |
| Absorption coefficient | 1.527 $mm^{-1}$ | |
| F(000) | 616 | |
| Crystal size | 0.200 x 0.070 x 0.020 $mm^3$ | |
| Theta range for data collection | 2.189 to 72.499° | |
| Index ranges | -7<=h<=8, -12<=k<=12, -24<=l<=24 | |
| Reflections collected | 62687 | |
| Independent reflections | 5732 [R(int) = 0.0566] | |
| Completeness to theta = 67.679° | 100.0 % | |
| Absorption correction | Semi-empirical from equivalents | |
| Max. and min. transmission | 0.7467 and 0.6672 | |
| Refinement method | Full-matrix least-squares on $F^2$ | |
| Data / restraints / parameters | 5732 / 1 / 358 | |
| Goodness-of-fit on $F^2$ | 1.092 | |
| Final R indices [I>2sigma(I)] | R1 = 0.0413, wR2 = 0.1106 | |
| R indices (all data) | R1 = 0.0427, wR2 = 0.1154 | |
| Absolute structure parameter | 0.054(4) | |
| Extinction coefficient | n/a | |
| Largest diff. peak and hole | 0.986 and -0.382 e.$Å^{-3}$ | |

## 7. Computational Investigation of the Key Step

### 7.1 Computational Details

The conformational space for each structure was explored with the OPLS3 force field[6] and a modified Monte Carlo search algorithm implemented in Macromodel.[7] An energy cut-off of 84 kJ $mol^{-1}$ was employed for the conformational analysis, and structures with heavy-atom root-mean-square deviations (RMSD) up to 0.5 Å after the force field optimizations where considered to be the same conformer. All remaining structures were subsequently optimized with the TPSS functional[8] with Grimme's dispersion correction D3 (Becke-Johnson damping),[9] the double-ζ basis set 6-31+G(d,p), and the integral equation formalism polarizable continuum model (IEFPCM)[10] for methanol. A grid with 99 radial shells per atom and 974 angular points per shell was used for the numerical integration of the density. Density fitting was furthermore used to accelerate the calculations. Vibrational analysis verified that each structure was a minimum. Thermal corrections were obtained from unscaled harmonic vibrational frequencies at the same level of theory for a standard state of 1 mol $L^{-1}$ and 298.15 K. Entropic contributions to free energies were obtained from partition functions evaluated with Grimme's quasi-harmonic approximation.[11] This method employs the free-rotor approximation for all frequencies below 100 $cm^{-1}$, the rigid-rotor-harmonic-oscillator (RRHO) approximation for all frequencies above 100 $cm^{-1}$, and a damping function to interpolate between the two expressions. Similar results were obtained from partition functions evaluated with Cramer's and Truhlar's quasiharmonic approximation.[12] This method uses the same approximations as the usual harmonic oscillator approximation, except that all vibrational frequencies lower than 100 $cm^{-1}$ are set equal to 100 $cm^{-1}$. Electronic energies were subsequently obtained from single point calculations of the TPSS-D3BJ geometries employing Neese's domain-based local pair-natural orbital (DLPNO) approach to the CCSD(T) method [DLPNO-CCSD(T)] with the default normalPNO settings,[13] the triple-ζ def2-TZVPPD[14] in combination with the corresponding auxiliary basis set[15] and the SMD

continuum model for methanol.[16] All density functional theory calculations were performed with Gaussian 16,[17] while the DLPNO-CCSD(T) calculations were performed with ORCA 4.[18]

### 7.2 Calculated Energies and Cartesian Coordinates

## Structure A

| | |
|---|---|
| SCF energy: | –1157.832862 hartree |
| Zero-point correction: | +0.523690 hartree |
| Enthalpy correction: | +0.551045 hartree |
| Free energy correction: | +0.470697 hartree |
| Truhlar's Delta G correction: | +0.473243 hartree |
| Grimme's Delta G correction: | +0.473233 hartree |

**Cartesian Coordinates**

| | | | |
|---|---|---|---|
| C | 2.63975 | -1.61717 | -0.81721 |
| C | 4.13220 | -1.69023 | -0.44119 |
| C | 4.81425 | -0.34438 | -0.67461 |
| H | 4.62937 | -2.46697 | -1.03622 |
| H | 4.26576 | -1.96192 | 0.61855 |
| C | 4.11039 | 0.76329 | 0.12114 |
| H | 4.78243 | -0.10557 | -1.74851 |
| O | 6.19556 | -0.48398 | -0.25780 |
| C | 2.61260 | 0.83020 | -0.21777 |
| H | 4.25634 | 0.58052 | 1.19365 |
| H | 4.56111 | 1.74359 | -0.09118 |
| C | 1.86924 | -0.52675 | -0.03322 |
| H | 2.16575 | -2.59155 | -0.64922 |
| H | 2.54875 | -1.39599 | -1.88935 |
| C | 0.41770 | -0.37048 | -0.59612 |
| C | 1.83354 | -0.85144 | 1.49954 |
| C | -0.25796 | 0.91190 | -0.12967 |
| C | 0.42595 | 1.93625 | 0.45299 |
| C | 1.87770 | 1.93648 | 0.57423 |
| O | 2.45946 | 1.17553 | -1.62924 |
| C | -0.46227 | -1.62848 | -0.33811 |
| H | 0.50780 | -0.26106 | -1.69024 |
| C | -1.94928 | -1.44909 | -0.69813 |
| H | -0.38035 | -1.92279 | 0.71355 |
| H | -0.04837 | -2.46170 | -0.91956 |
| C | -2.55268 | -0.22535 | 0.01676 |
| H | -2.48004 | -2.37179 | -0.43378 |
| H | -2.06421 | -1.30984 | -1.78525 |
| C | -1.72038 | 1.00984 | -0.45030 |
| C | -2.53536 | 2.22804 | 0.00714 |
| H | -1.76830 | 0.97532 | -1.55610 |
| C | -4.00984 | 1.75286 | -0.14388 |
| H | -2.31528 | 3.12141 | -0.58946 |
| H | -2.31257 | 2.46962 | 1.05495 |
| C | -3.97455 | 0.21656 | -0.45138 |
| H | -4.52673 | 2.29763 | -0.94242 |
| H | -4.57012 | 1.93800 | 0.78326 |
| C | -2.49547 | -0.37953 | 1.55267 |

| | | | |
|---|---|---|---|
| H | -3.99703 | 0.08104 | -1.54743 |
| C | -5.20426 | -0.51916 | 0.13106 |
| H | 6.65332 | 0.35337 | -0.45697 |
| H | 2.95626 | 2.00105 | -1.78347 |
| O | 1.64387 | -2.24492 | 1.81298 |
| H | 2.48185 | -2.71321 | 1.64868 |
| H | 0.98410 | -0.33818 | 1.96285 |
| H | 2.74447 | -0.49101 | 1.99401 |
| O | 2.50727 | 2.82285 | 1.18259 |
| H | -0.09032 | 2.83167 | 0.79603 |
| H | -2.98681 | -1.30937 | 1.86639 |
| H | -2.99963 | 0.45310 | 2.05872 |
| H | -1.46082 | -0.40623 | 1.91713 |
| H | -5.21840 | -0.34266 | 1.21935 |
| C | -5.18451 | -2.04082 | -0.10973 |
| C | -6.50300 | 0.07242 | -0.45780 |
| H | -5.05792 | -2.26496 | -1.17876 |
| H | -6.13194 | -2.48939 | 0.21913 |
| H | -4.37556 | -2.53608 | 0.43946 |
| H | -6.59794 | 1.14483 | -0.24752 |
| H | -7.38244 | -0.43393 | -0.03760 |
| H | -6.52438 | -0.06261 | -1.54945 |

**Structure B**

| | |
|---|---|
| SCF energy: | –1157.846611 hartree |
| Zero-point correction: | +0.525374 hartree |
| Enthalpy correction: | +0.551501 hartree |
| Free energy correction: | +0.474061 hartree |
| Truhlar's Delta G correction: | +0.476144 hartree |
| Grimme's Delta G correction: | +0.476296 hartree |

**Cartesian Coordinates**

| | | | |
|---|---|---|---|
| C | 2.55397 | -1.93030 | -0.16553 |
| C | 3.93606 | -1.95351 | 0.51367 |
| C | 4.82787 | -0.85279 | -0.06881 |
| H | 4.40494 | -2.93689 | 0.36699 |
| H | 3.83723 | -1.79334 | 1.59809 |
| C | 4.17757 | 0.51759 | 0.11786 |
| H | 4.98762 | -1.04445 | -1.14186 |
| O | 6.11355 | -0.77970 | 0.59709 |
| C | 2.77454 | 0.57709 | -0.50255 |
| H | 4.80367 | 1.30473 | -0.32277 |
| H | 4.11146 | 0.73439 | 1.19288 |
| C | 1.84249 | -0.57234 | -0.00452 |
| H | 1.92189 | -2.72217 | 0.25451 |
| H | 2.67008 | -2.14041 | -1.23795 |
| C | 0.47139 | -0.46958 | -0.73355 |
| C | 1.39763 | -0.27674 | 1.44819 |
| C | -0.10234 | 0.78960 | -0.05055 |
| C | 0.63146 | 2.04675 | -0.58635 |
| C | 2.10755 | 1.95394 | -0.25701 |
| O | 2.84744 | 0.43600 | -1.95259 |
| C | -0.41126 | -1.72554 | -0.56735 |
| H | 0.61883 | -0.27749 | -1.80275 |
| C | -1.90566 | -1.46415 | -0.85069 |
| H | -0.31260 | -2.12881 | 0.45014 |
| H | -0.03960 | -2.50550 | -1.24500 |
| C | -2.45460 | -0.35470 | 0.06971 |

| | | | |
|---|---|---|---|
| H | -2.45454 | -2.40489 | -0.72224 |
| H | -2.03812 | -1.15254 | -1.89966 |
| C | -1.61345 | 0.92212 | -0.21757 |
| C | -2.36238 | 2.04878 | 0.51322 |
| H | -1.74393 | 1.10443 | -1.30023 |
| C | -3.85585 | 1.64725 | 0.32995 |
| H | -2.14562 | 3.03907 | 0.09502 |
| H | -2.08188 | 2.06774 | 1.57394 |
| C | -3.87390 | 0.20328 | -0.28152 |
| H | -4.38240 | 2.35357 | -0.32280 |
| H | -4.38172 | 1.65195 | 1.29529 |
| C | -2.41845 | -0.79775 | 1.55042 |
| H | -3.91423 | 0.29108 | -1.38214 |
| C | -5.12341 | -0.59454 | 0.15979 |
| H | 6.57222 | -1.62657 | 0.44680 |
| H | 3.54762 | 1.03546 | -2.27372 |
| O | 0.27932 | 0.64630 | 1.35936 |
| H | 1.06390 | -1.20117 | 1.94441 |
| H | 2.17310 | 0.18761 | 2.06861 |
| O | 2.75383 | 2.91390 | 0.16718 |
| H | -1.39727 | -0.97523 | 1.89849 |
| H | -2.99754 | -1.72224 | 1.67792 |
| H | -2.85793 | -0.03610 | 2.20664 |
| H | -5.12375 | -0.64228 | 1.26121 |
| C | -5.16056 | -2.03518 | -0.38514 |
| C | -6.40552 | 0.14667 | -0.27707 |
| H | -5.04481 | -2.04138 | -1.47867 |
| H | -6.12281 | -2.50812 | -0.14488 |
| H | -4.36833 | -2.65962 | 0.04335 |
| H | -6.43910 | 0.24079 | -1.37279 |
| H | -6.46263 | 1.15505 | 0.15095 |
| H | -7.29901 | -0.40718 | 0.04162 |
| H | 0.23532 | 2.96386 | -0.13782 |
| H | 0.50777 | 2.10887 | -1.67761 |

## Structure A'

| | |
|---|---|
| SCF energy: | –1157.330890 hartree |
| Zero-point correction: | +0.508844 hartree |
| Enthalpy correction: | +0.534838 hartree |
| Free energy correction: | +0.457255 hartree |
| Truhlar's Delta G correction: | +0.459657 hartree |
| Grimme's Delta G correction: | +0.459668 hartree |

## Cartesian Coordinates

| | | | |
|---|---|---|---|
| C | 2.62244 | -1.74227 | -0.83958 |
| C | 4.03623 | -1.43130 | -1.37594 |
| C | 4.85124 | -0.59840 | -0.37382 |
| H | 3.96375 | -0.87786 | -2.32338 |
| H | 4.56281 | -2.37533 | -1.57873 |
| C | 4.15264 | 0.76948 | -0.13899 |
| H | 5.85045 | -0.40546 | -0.79790 |
| O | 5.04035 | -1.30893 | 0.85228 |
| C | 2.63439 | 0.77097 | -0.39495 |
| H | 4.33988 | 1.09162 | 0.88913 |
| H | 4.59723 | 1.52895 | -0.80249 |
| C | 1.90923 | -0.56476 | -0.08783 |
| H | 2.68211 | -2.59527 | -0.15003 |
| H | 1.99811 | -2.06913 | -1.68358 |

| | | | |
|---|---|---|---|
| C | 0.43035 | -0.44349 | -0.59170 |
| C | 1.88298 | -0.83352 | 1.47486 |
| C | -0.23810 | 0.86564 | -0.20475 |
| C | 0.46476 | 1.93657 | 0.25398 |
| C | 1.92268 | 1.94570 | 0.31256 |
| O | 2.39015 | 1.02446 | -1.82974 |
| C | -0.43544 | -1.67841 | -0.20486 |
| H | 0.46643 | -0.42202 | -1.69531 |
| C | -1.92937 | -1.53802 | -0.55358 |
| H | -0.34939 | -1.86809 | 0.87070 |
| H | -0.02013 | -2.56098 | -0.70880 |
| C | -2.52559 | -0.26658 | 0.08241 |
| H | -2.45243 | -2.44064 | -0.21399 |
| H | -2.06243 | -1.47790 | -1.64623 |
| C | -1.71129 | 0.93444 | -0.48985 |
| C | -2.52235 | 2.17803 | -0.09846 |
| H | -1.78938 | 0.82397 | -1.58919 |
| C | -3.99742 | 1.68818 | -0.18393 |
| H | -2.32096 | 3.02976 | -0.75943 |
| H | -2.27610 | 2.49173 | 0.92487 |
| C | -3.96015 | 0.13379 | -0.38371 |
| H | -4.53439 | 2.17360 | -1.00738 |
| H | -4.53972 | 1.93538 | 0.73964 |
| C | -2.43269 | -0.30769 | 1.62418 |
| H | -4.00627 | -0.07859 | -1.46692 |
| C | -5.17325 | -0.56324 | 0.27581 |
| H | 4.17229 | -1.05809 | 1.46317 |
| H | 2.97539 | 1.76180 | -2.08617 |
| O | 3.06634 | -0.59587 | 2.16763 |
| H | 1.05528 | -0.22041 | 1.89283 |
| H | 1.55459 | -1.89256 | 1.57343 |
| O | 2.55758 | 2.91019 | 0.78637 |
| H | -0.03846 | 2.86326 | 0.52911 |
| H | -2.94789 | 0.54750 | 2.07933 |
| H | -1.38919 | -0.28142 | 1.96326 |
| H | -2.89418 | -1.22349 | 2.01600 |
| H | -5.16545 | -0.30911 | 1.34875 |
| C | -5.15001 | -2.09818 | 0.14515 |
| C | -6.48762 | -0.02126 | -0.32604 |
| H | -6.08752 | -2.52614 | 0.52614 |
| H | -4.32617 | -2.54834 | 0.71071 |
| H | -5.04488 | -2.39858 | -0.90742 |
| H | -6.53077 | -0.23410 | -1.40464 |
| H | -6.58435 | 1.06311 | -0.19120 |
| H | -7.35548 | -0.49990 | 0.14754 |

**Structure B'**

| | |
|---|---|
| SCF energy: | –1157.346409 hartree |
| Zero-point correction: | +0.511502 hartree |
| Enthalpy correction: | +0.537189 hartree |
| Free energy correction: | +0.461094 hartree |
| Truhlar's Delta G correction: | +0.462767 hartree |
| Grimme's Delta G correction: | +0.462978 hartree |

**Cartesian Coordinates**

| | | | |
|---|---|---|---|
| C | 2.60404 | -1.89678 | -0.30976 |
| C | 3.97806 | -1.92427 | 0.39240 |
| C | 4.83653 | -0.73653 | -0.04307 |

| | | | |
|---|---|---|---|
| H | 4.50455 | -2.86241 | 0.16837 |
| H | 3.84291 | -1.87819 | 1.48466 |
| C | 4.11748 | 0.59616 | 0.20111 |
| H | 5.06235 | -0.83139 | -1.11573 |
| O | 6.08194 | -0.81231 | 0.70927 |
| C | 2.74060 | 0.61508 | -0.50014 |
| H | 4.70680 | 1.43791 | -0.18777 |
| H | 4.00408 | 0.74660 | 1.28443 |
| C | 1.86263 | -0.57453 | -0.02914 |
| H | 1.99861 | -2.74449 | 0.03877 |
| H | 2.74137 | -2.01369 | -1.39388 |
| C | 0.46381 | -0.41187 | -0.66926 |
| C | 1.50965 | -0.43262 | 1.49140 |
| C | -0.07750 | 0.72632 | 0.23296 |
| C | 0.70236 | 1.99436 | 0.00324 |
| C | 2.02947 | 1.98288 | -0.38056 |
| O | 3.00279 | 0.44857 | -1.94256 |
| C | -0.41547 | -1.67314 | -0.64454 |
| H | 0.56048 | -0.05676 | -1.70306 |
| C | -1.89924 | -1.35947 | -0.93935 |
| H | -0.33844 | -2.16708 | 0.33505 |
| H | -0.03861 | -2.39100 | -1.38758 |
| C | -2.47340 | -0.36482 | 0.09236 |
| H | -2.46896 | -2.29763 | -0.95159 |
| H | -1.98659 | -0.91555 | -1.94556 |
| C | -1.58279 | 0.91470 | 0.04710 |
| C | -2.34924 | 1.93159 | 0.90747 |
| H | -1.66439 | 1.26980 | -0.99711 |
| C | -3.83720 | 1.62297 | 0.56616 |
| H | -2.07625 | 2.96858 | 0.67739 |
| H | -2.14264 | 1.76450 | 1.97262 |
| C | -3.85215 | 0.28840 | -0.25933 |
| H | -4.29494 | 2.43994 | -0.00520 |
| H | -4.43361 | 1.50279 | 1.48237 |
| C | -2.55630 | -1.02432 | 1.48877 |
| H | -3.81616 | 0.54250 | -1.33426 |
| C | -5.15131 | -0.51801 | -0.02605 |
| H | 6.63210 | -0.05424 | 0.43874 |
| H | 3.20691 | 1.37102 | -2.21188 |
| O | 0.19850 | 0.15876 | 1.58780 |
| H | 2.23607 | 0.18660 | 2.03524 |
| H | 1.46629 | -1.41940 | 1.97679 |
| O | 2.76039 | 2.99965 | -0.75908 |
| H | -3.17584 | -1.93066 | 1.43678 |
| H | -3.01542 | -0.35141 | 2.22488 |
| H | -1.56259 | -1.28923 | 1.85919 |
| H | -5.22833 | -0.73327 | 1.05258 |
| C | -5.19241 | -1.85634 | -0.78826 |
| C | -6.38010 | 0.32866 | -0.42310 |
| H | -5.00222 | -1.69975 | -1.86008 |
| H | -6.18171 | -2.32397 | -0.68572 |
| H | -4.44772 | -2.56742 | -0.41315 |
| H | -6.43815 | 1.26173 | 0.15088 |
| H | -6.33591 | 0.59017 | -1.49109 |
| H | -7.30888 | -0.23270 | -0.25156 |
| H | 0.18084 | 2.95027 | 0.09060 |

## 8. References


[1] a) J. I. Concepción, C. G. Francisco, R. Hernández, J. A. Salazar, E. Suárez, *Tetrahedron Lett.* **1984**, *25*, 1953–1956; b) P. de Armas, J. I. Concepción, C. G. Francisco, R. Hernández, J. A. Salazar, E. Suárez, *J. Chem. Soc. Perkin Trans. 1* **1989**, 405–411.

[2] H. Mastalerz, P. Morand, *J. Chem. Soc. Perkin Trans 1* **1981**, 154–160.

[3] a) P. Morand, A. Van Tongerloo, *Steroids* **1973**, *21*, 65–85; b) G. Snatzke, B. Wßling, *Liebigs Ann. Chem.* **1979**, 1028–1035.

[4] J. Joska, J. Fajkoš, *Collect. Czech. Chem. Commun.* **1978**, *43*, 3433–3443.

[5] C. Boonlarppradab, D. J. Faulkner, *J. Nat. Prod.* **2007**, *70*, 846–848.

[6] E. Harder, W. Damm, J. Maple, C. Wu, M. Reboul, J. Y. Xiang, L. Wang, D. Lupyan, M. K. Dahlgren, J. L. Knight, J. W. Kaus, D. S. Cerutti, G. Krilov, W. L. Jorgensen, R. Abel, R. A. Friesner, *J. Chem. Theory Comput.* **2016**, *12*, 281–296.

[7] Schrödinger Release 2019−2; MacroModel, Schrödinger, LLC: New York, NY, 2017.

[8] J. Tao, J. P. Perdew, V. N. Staroverov, G. E. Scuseria, *Phys. Rev. Lett.* **2003**, *91*, 146401.

[9] S. Grimme, S. Ehrlich, L. Goerigk, *J. Comput. Chem.* **2011**, *32*, 1456–1465.

[10] E. Cancès, B. Mennucci, J. Tomasi, *J. Chem. Phys.* **1997**, *107*, 3032–3041.

[11] S. Grimme, *Chem. Eur. J.* **2012**, *18*, 9955–9964.

[12] R. F. Ribeiro, A. V. Marenich, C. J. Cramer, D. G. Truhlar, *J. Phys. Chem. B* **2011**, *115*, 14556–14562.

[13] a) C. Riplinger, B. Sandhoefer, A. Hansen, F. Neese, *J. Chem. Phys.* **2013**, *139*, 134101; b) C. Riplinger, F. Neese, *J. Chem. Phys.* **2013**, *138*, 034106; c) D. G. Liakos, F. Neese, *J. Chem. Theory Comput.* **2015**, *11*, 4054–4063.

[14] a) F. Weigend, R. Ahlrichs, *Phys. Chem. Chem. Phys.* **2005**, *7*, 3297–3305; b) D. Rappoport, F. Furche, *J. Chem. Phys.* **2010**, *133*, 134105.

[15] A. Hellweg, C. Hättig, S. Höfener, W. Klopper, *Theor. Chem. Acc.* **2007**, *117*, 587–597.

[16] A. V. Marenich, C. J. Cramer, D. G. Truhlar, *J. Phys. Chem. B* **2009**, *113*, 6378–6396.

[17] Gaussian16, Revision B.01; M. J. Frisch, G. W. Trucks, H. B. Schlegel, G. E. Scuseria, M. A. Robb, J. R. Cheeseman, G. Scalmani, V. Barone, G. A. Petersson, H. Nakatsuji, X. Li, M. Caricato, A. V. Marenich, J. Bloino, B. G. Janesko, R. Gomperts, B. Mennucci, H. P. Hratchian, J. V. Ortiz, A. F. Izmaylov, J. L. Sonnenberg, Williams, F. Ding, F. Lipparini, F. Egidi, J. Goings, B. Peng, A. Petrone, T. Henderson, D. Ranasinghe, V. G. Zakrzewski, J. Gao, N. Rega, G. Zheng, W. Liang, M. Hada, M. Ehara, K. Toyota, R. Fukuda, J. Hasegawa, M. Ishida, T. Nakajima, Y. Honda, O. Kitao, H. Nakai, T. Vreven, K. Throssell, J. A. Montgomery Jr., J. E. Peralta, F. Ogliaro, M. J. Bearpark, J. J. Heyd, E. N. Brothers, K. N. Kudin, V. N. Staroverov, T. A. Keith, R. Kobayashi, J. Normand, K. Raghavachari, A. P. Rendell, J. C. Burant, S. S. Iyengar, J. Tomasi, M. Cossi, J. M. Millam, M. Klene, C. Adamo, R. Cammi, J. W. Ochterski, R. L. Martin, K. Morokuma, O. Farkas, J. B. Foresman, D. J. Fox, Wallingford, CT, **2016**.

[18] F. Neese, *Wiley Interdiscip. Rev. Comput. Mol. Sci.* **2012**, *2*, 73–78.
